# Supplementary material for: Bi-allelic WDHD1 variants cause microcephalic primordial dwarfism
Source: Am J Hum Genet. 2026 Apr 9;113(5):1067–89. doi: 10.1016/j.ajhg.2026.03.010 (PMC13277691; doi:10.1016/j.ajhg.2026.03.010)
Supplement: Document S1. Figures S1–S14, Tables S1–S3, and supplemental material and methods [file mmc1.pdf]

The American Journal of Human Genetics, Volume *113*

Supplemental information

Bi-allelic *WDHD1* variants

cause microcephalic primordial dwarfism

Debora Tibbe, Marie Ronja Vogt, Tess Holling, Lea Dewi Schlieben, Fanny Kortüm, Moneef Shoukier, Christoph Bagowski, Felix Distelmaier, Luisa Averdunk, Alexej Knaus, Peter Krawitz, Alma Kuechler, Elke Lainka, Amelie Stalke, Sandra von Hardenberg, Bernd Auber, Eva-Doreen Pfister, Bruno Reversade, Anthony Sabbagh, Aida M. Bertoli-Avella, Salem Alawbathani, Elizabeth E. Palmer, Manisha Chauhan, Rocio Rius, Yoonji Kim, Australian Undiagnosed Diseases Network (UDN-Aus), Dzhoy Papingi, Deborah Bartholdi, Dominique Braun, Oliver Maier, April Dinwiddie, Elisabeth Steichen-Gersdorf, Andreas R. Janecke, Anatoly Tiulpakov, Nikolay Zernov, Maria Izabel Arismendi, Alexander A.L. Jorge, Himanshu Goel, Lauren Dreyer, Lily Loughman, Holger Prokisch, Kerstin Borgmann, and Kerstin Kutsche

Supplemental Information

Supplemental figures and legends

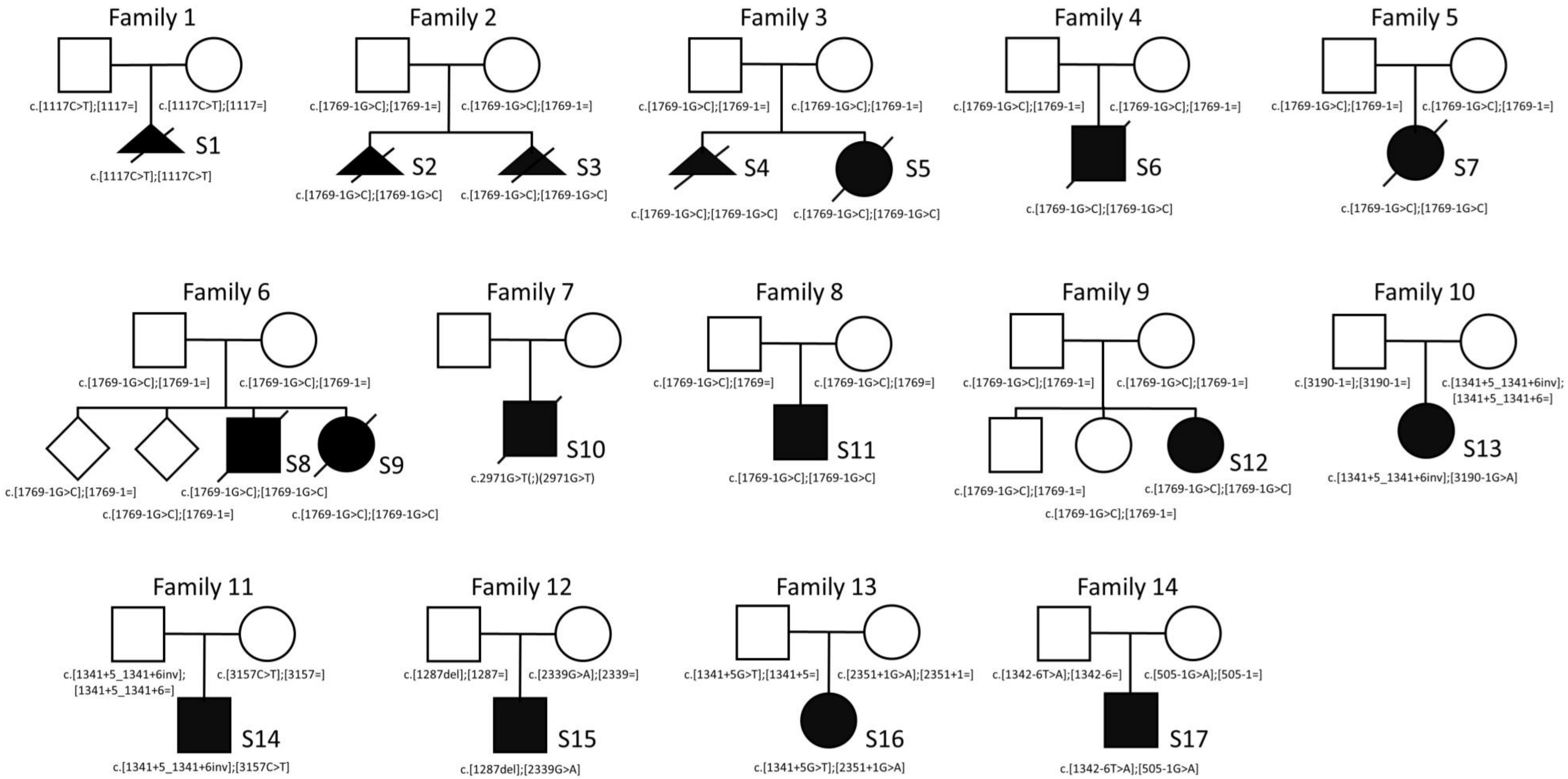

Figure S1. Pedigrees of 17 subjects with biallelic *WDHD1* variants.

Males are represented by squares and females by circles. Triangles indicate pregnancies not carried to term. Filled symbols denote individuals affected by the disease, and a diagonal slash indicates a deceased individual. The genotype, if known, is shown below each symbol.

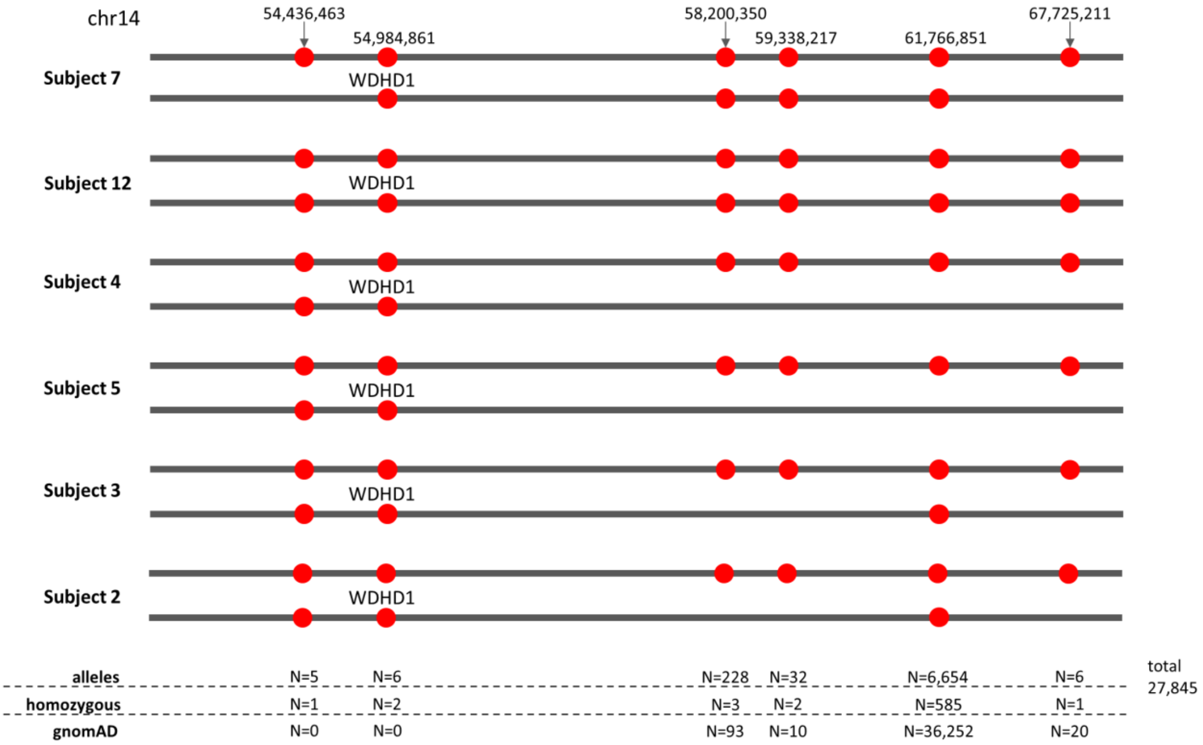

Figure S2. A specific haplotype is shared among subjects with the *WDHD1* variant c.1769-1G>C*.*

Analysis of the haplotype based on genotypes of rare single nucleotide variants on chromosome 14 in an in-house database with 27,845 WES cases and in gnomAD for subjects 2-5, 7, and 12 carrying the homozygous splice site variant c.1769-1G>C in *WDHD1*. Schematic visualization of selected rare variants (red dots) on chromosome 14 (grey line) in proximity to *WDHD1* and the shared homozygous *WDHD1* variant c.1769-1G>C. Chromosome positions are indicated according to GRCh38. Allele count and number of homozygous individuals in the in-house database as well as the allele frequency in the gnomAD database (v2.1.1) are given below the haplotypes.

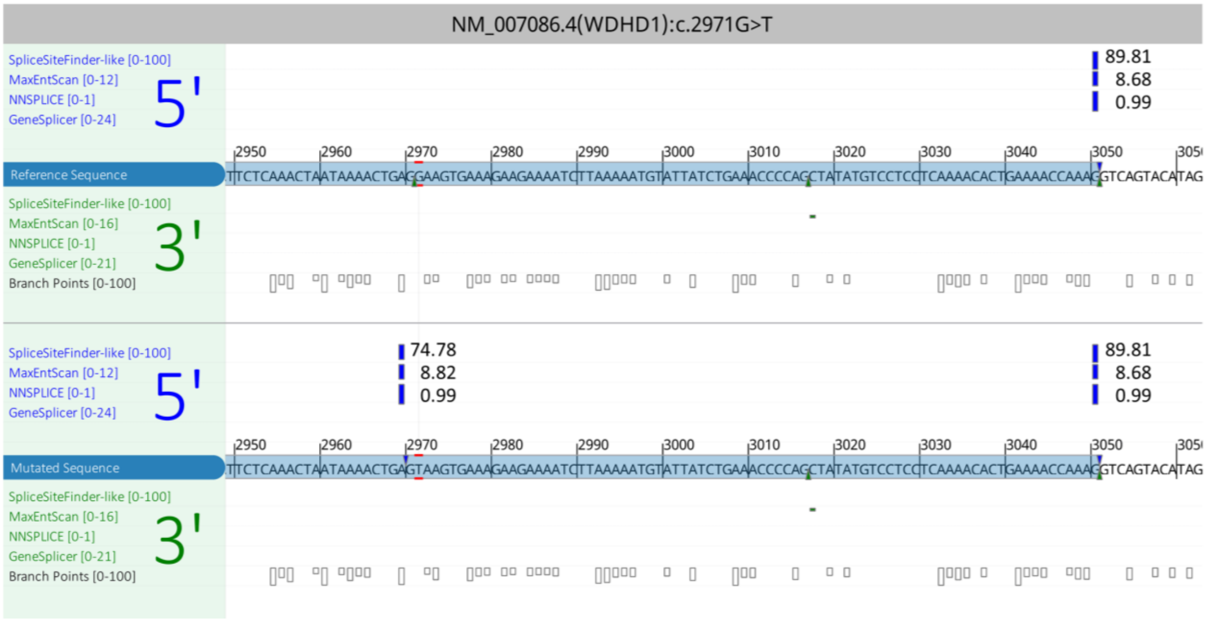

Figure S3. Splice site predictions for the homozygous *WDHD1* nonsense variant c.2971G>T identified in subject 10.

Screenshots from the splicing module of Alamut Visual Plus (v1.4; SOPHIA GENETICS) show partial sequences of the exon 24-intron 24 boundary of *WDHD1* (NM_007086.4). The reference sequence is displayed in the upper panel and the sequence with the variant c.2971G>T in the lower panel. The exonic sequence is highlighted in blue. Splice sites were predicted using the programs SpliceSiteFinder-like, NNSPLICE 0.9 version, MaxEntScan, and GeneSplicer. Predicted splice donor sites are marked by blue rectangles within the sequence. Corresponding scores are provided (see Table 2) and are displayed on the right of the blue boxes shown above the sequence.

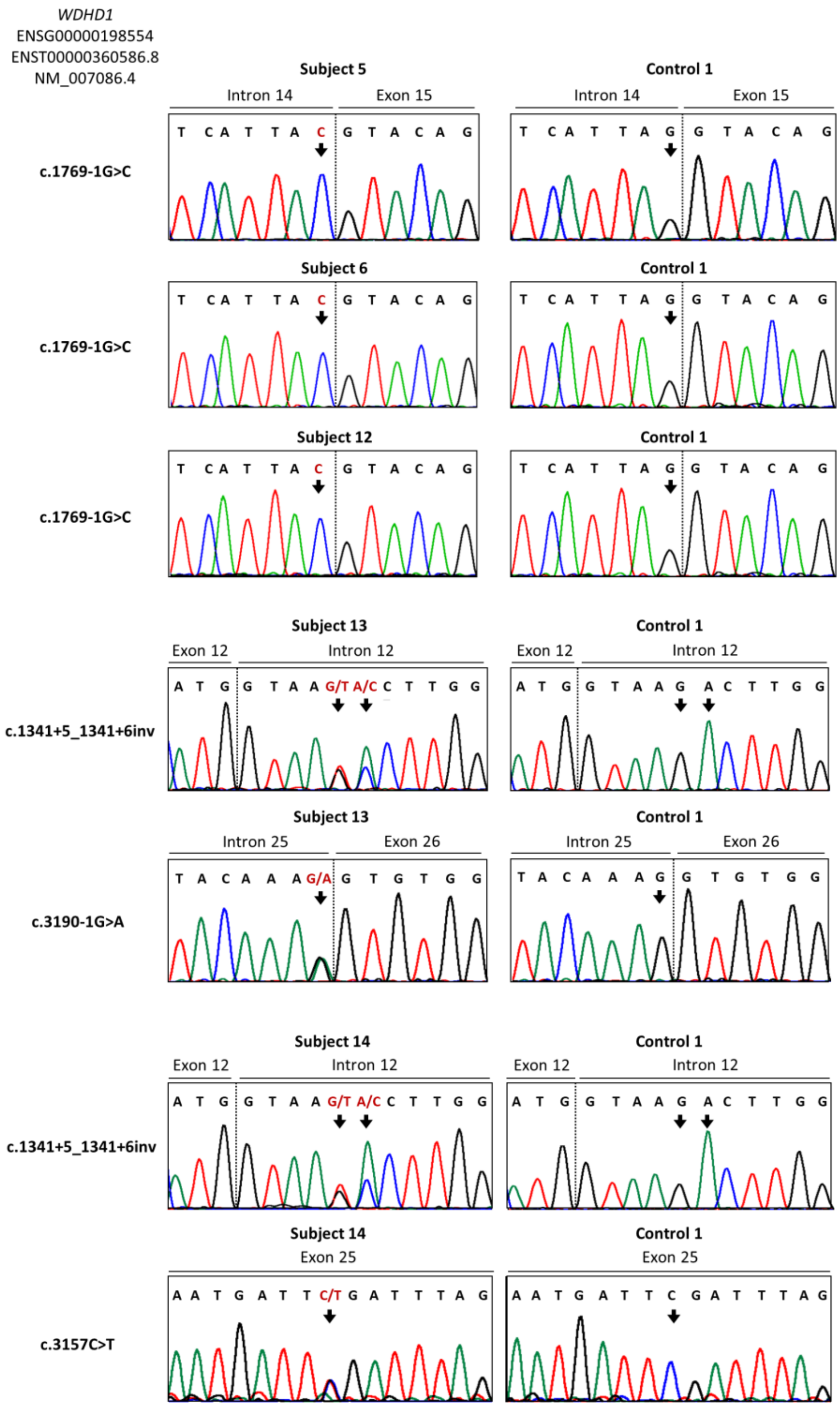

Figure S4. Validation of biallelic *WDHD1* variants in subject-derived fibroblasts.

Partial sequence electropherograms show the *WDHD1* splice site variant c.1769-1G>C in the homozygous state in DNA of fibroblasts derived from subjects 5, 6, and 12, compared to the reference sequence (control 1). Sanger sequencing confirmed the presence of the heterozygous variant c.1341+5_1241+6inv in fibroblasts of subjects 13 and 14, the heterozygous variant c.3190-1G>A in subject 13 cells, and the heterozygous variant c.3157C>T in subject 14 cells. The respective reference sequence is shown on the right (control 1). Arrows indicate the positions of the variants (highlighted in red) in the sequence derived from subject fibroblasts, as well as the corresponding positions in the reference sequence (control 1). Exon-intron boundaries are marked with dotted lines.

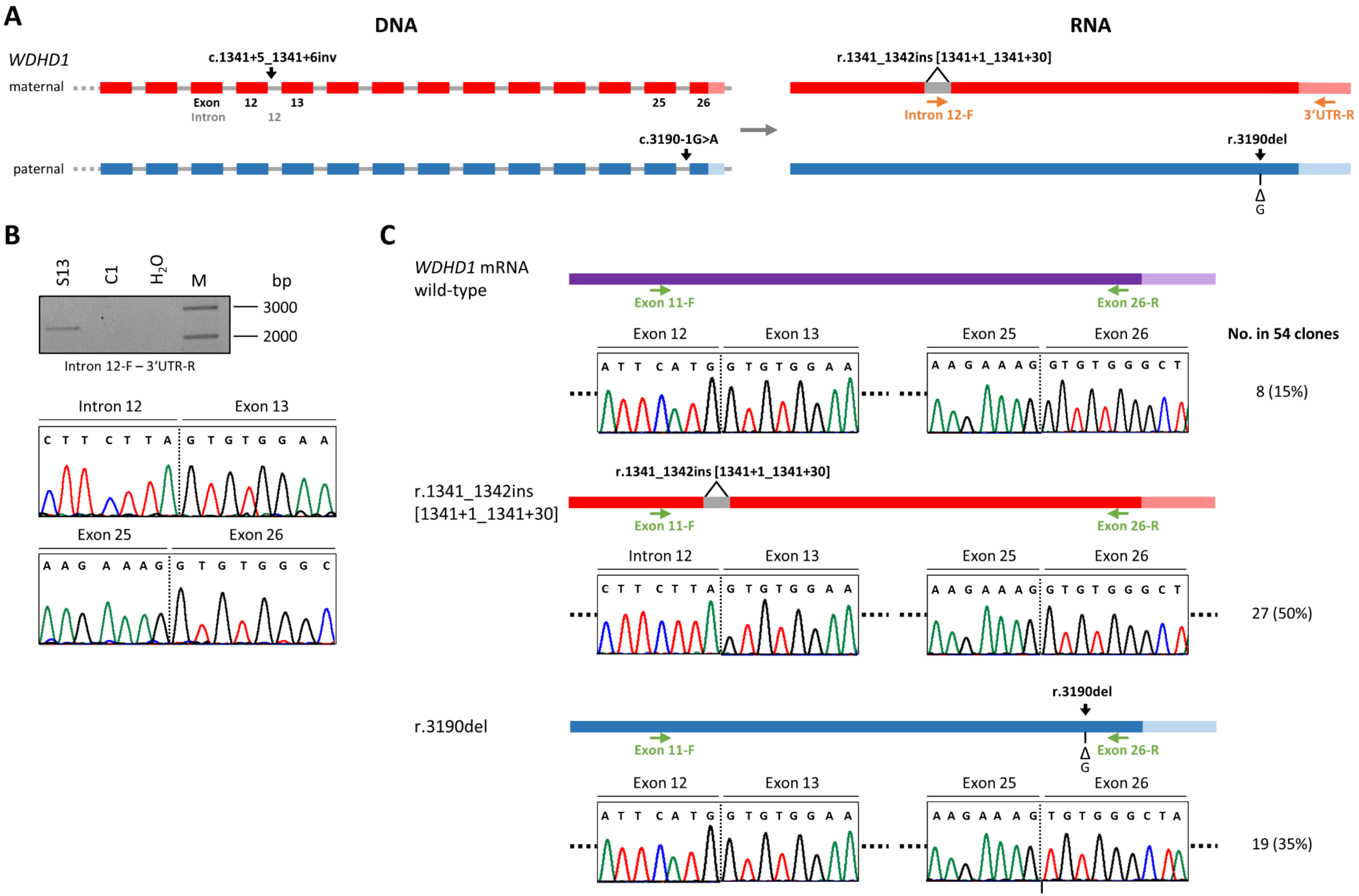
A
DNA
RNA
WDHD1
maternal
c.1341+5_1341+6inv
Exon 12 13 25 26
Intron 12
paternal
c.3190-1G>A
r.1341_1342ins [1341+1_1341+30]
Intron 12-F
3'UTR-R
r.3190del
ΔG
B
S13 C1 H2O M bp
3000
2000
Intron 12-F – 3'UTR-R
Intron 12
Exon 13
Exon 25
Exon 26
C
WDHD1 mRNA wild-type
Exon 11-F
Exon 26-R
Exon 12
Exon 13
Exon 25
Exon 26
No. in 54 clones
8 (15%)
r.1341_1342ins [1341+1_1341+30]
Intron 12
27 (50%)
r.3190del
19 (35%)

Figure S5. Determination of the allelic phase of the two *WDHD1* variants in subject 13.

(A) Schematic representation of the partial *WDHD1* gene (DNA, left) and *WDHD1* mRNA (right) derived from the maternal (red) and paternal (blue) alleles. Coding regions are shown as red or blue boxes and intronic regions as grey lines. 3' untranslated regions (3'UTRs) are shown in light red or light blue. Arrows indicate the positions of the variants. Black arrows and lines indicate the results of aberrant splicing events on the *WDHD1* transcripts (right). The localization of the primers used for specific amplification of a *WDHD1* transcript with the 30 bp intronic insertion is shown in orange. ΔG, deletion of a guanine. (B) Agarose gel showing an RT-PCR amplicon (2286 bp) specifically generated from leukocyte-derived RNA (cDNA) of subject 13 (S13). The used primers are shown in (A, right), spanning the first 30 bp of intron 12 to the 3'UTR. Sanger sequencing of this RT-PCR product revealed the intron 12-exon 13 junction together with canonically spliced exons 25 and 26. (C) Schematic representation of *WDHD1* transcript structure and RT-PCR strategy used to assess the allelic phase of the c.1341+5_1341+6inv and the c.3190-1G>A variants. The positions of aberrant splicing effects (black) and the primer pair used (green) are indicated. RT-PCR products amplified with primers spanning exon 11 to exon 26 were cloned, and inserts from individual clones were subsequently Sanger sequenced. Representative Sanger traces show the exon 12-exon 13 and exon 25-exon 26 junctions within the same representative cloned insert. The proportions of the different *WDHD1* transcripts, including the wild-type (purple), transcripts with r.1341_1342ins[1341+1_1341+30] (red), and transcripts with r.3190del (blue), among the 54 analyzed clones are shown as percentages to the right of the Sanger sequencing traces.

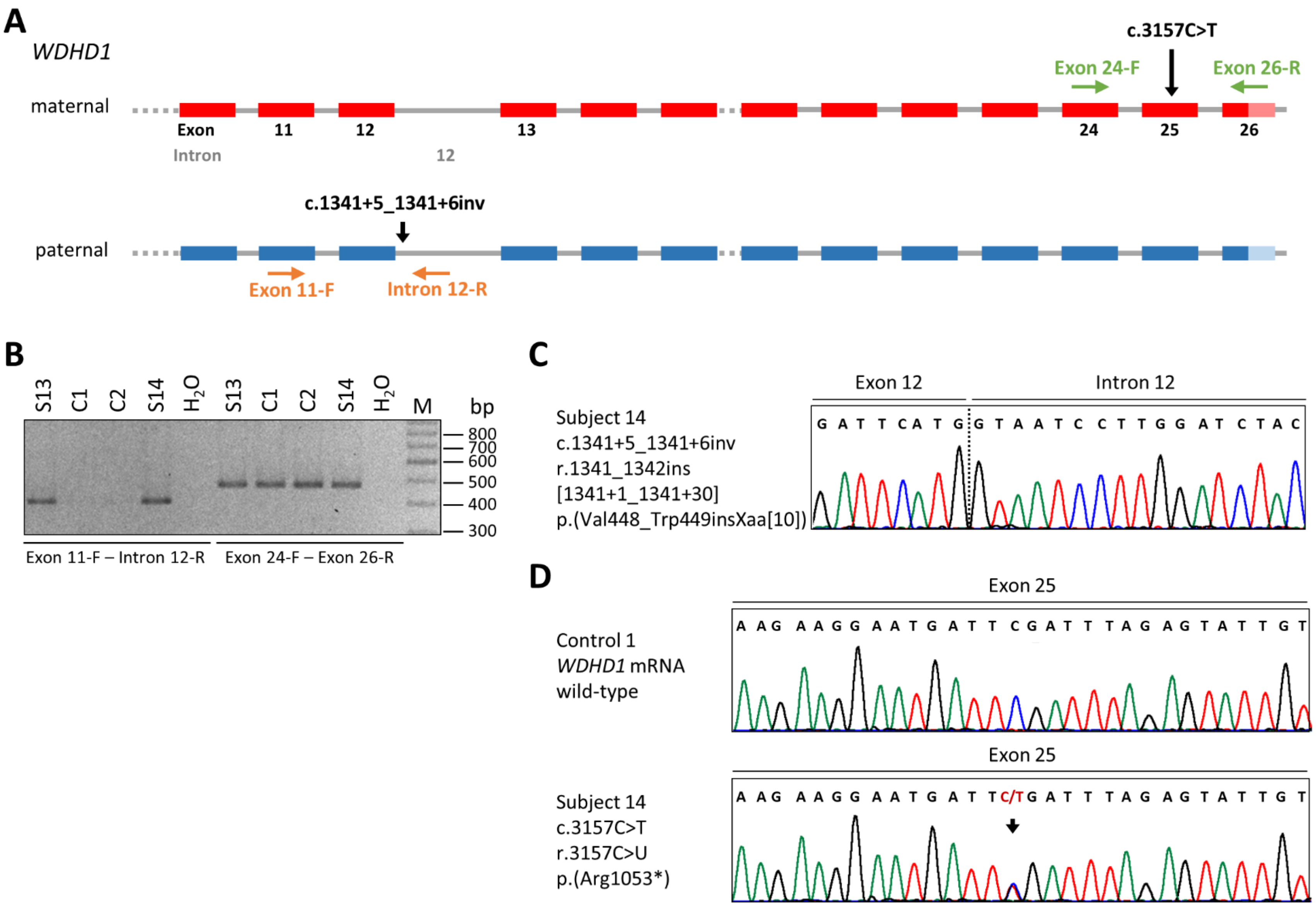
A
WDHD1
maternal
c.3157C>T
Exon 24-F
Exon 26-R
Exon
11
12
13
24
25
26
Intron
12
c.1341+5_1341+6inv
paternal
Exon 11-F
Intron 12-R
B
S13
C1
C2
S14
H2O
S13
C1
C2
S14
H2O
M
bp
800
700
600
500
400
300
Exon 11-F – Intron 12-R
Exon 24-F – Exon 26-R
C
Exon 12
Intron 12
Subject 14
c.1341+5_1341+6inv
r.1341_1342ins
[1341+1_1341+30]
p.(Val448_Trp449insXaa[10])
G A T T C A T G G T A A T C C T T G G A T C T A C
D
Exon 25
Control 1
WDHD1 mRNA
wild-type
A AG A A G G A A T G A T T C G A T T T A G A G T A T T G T
Exon 25
Subject 14
c.3157C>T
r.3157C>U
p.(Arg1053*)
A AG A A G G A A T G A T T C/T G A T T T A G A G T A T T G T

Figure S6. Analysis of *WDHD1* transcripts in fibroblasts of subject 14 harboring the variants c.1341+5_1341+6inv and c.3157C>T.

(A) Schematic representation of partial *WDHD1* gene and the RT-PCR primers used to detect the insertion of the first 30 bp of intron 12 and to assess the effect of the nonsense variant c.3157C>T. Coding regions are shown as red or blue boxes, intronic regions as grey lines, and 3' untranslated regions as light red and light blue boxes. The positions of primers used for amplification of the exon 24-exon 26 RT-PCR product (green) and the PCR product containing the intron 12 insertion (orange) are indicated. Black arrows mark the positions of the variants. (B) Agarose gel showing RT-PCR amplicons generated from fibroblast-derived RNA (cDNA) of fibroblasts from subjects 13 and 14 (S13, S14) and two controls (C1, C2). Primers spanning exon 11 to intron 12 produced the expected 406-bp product in subject 13 and 14 cells but not in control cells. The primer combination spanning exon 24 to exon 26 yielded a PCR product of 470 bp in subject and control cells. (C) Partial Sanger trace of the exon 11-intron 12 RT-PCR product obtained from subject 14 cells (shown in B). Sequencing confirmed splicing of exon 12 to the first 30 bp of intron 12 in subject 14 cells. (D) Partial Sanger traces of the exon 24-exon 26 RT-PCR product obtained from control 1 and subject 14 cells (shown in B). Sequencing of the 470-bp amplicons revealed wild-type *WDHD1* transcripts in control 1 cells, whereas fibroblasts from subject 14 expressed both the wild-type *WDHD1* transcript (r.3157C) and the variant transcript (r.3157U; indicated by a black arrow) in approximately equal amounts.

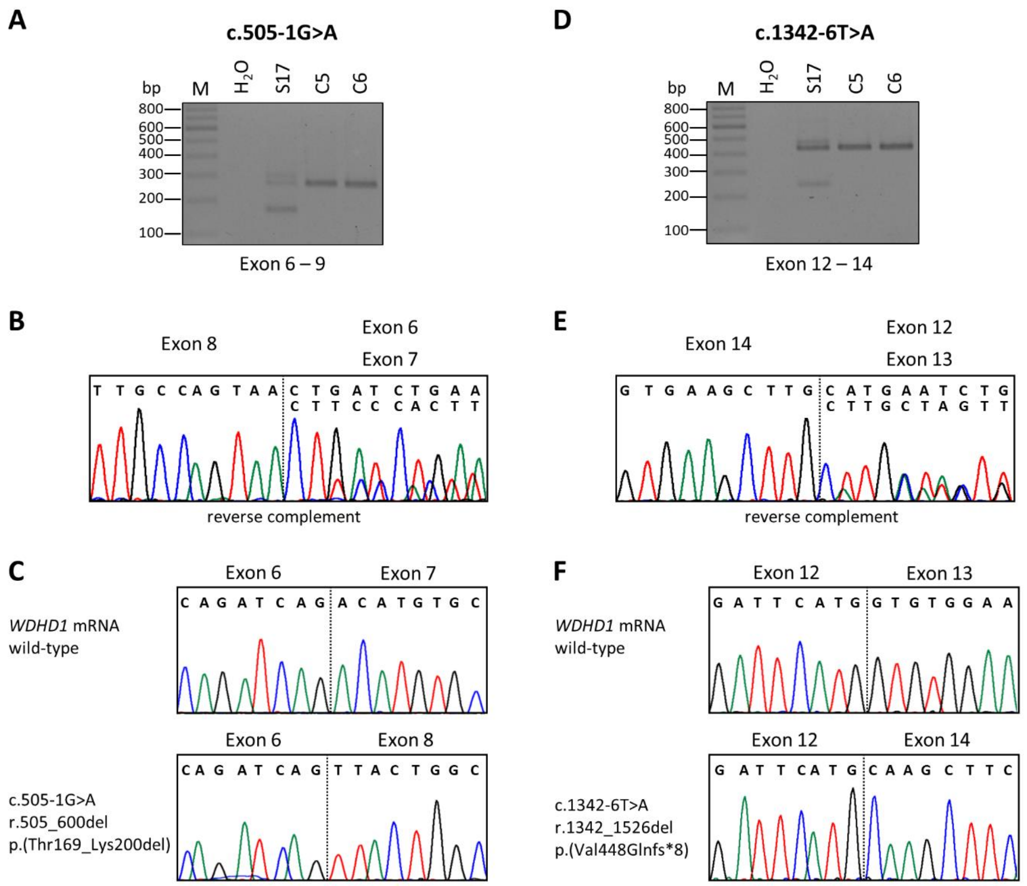

Figure S7: Analysis of *WDHD1* transcripts in leukocytes of S17 harboring the variants c.505-1G>A and c.1342-6T>A.

(A) Agarose gel showing RT-PCR amplicons generated from leukocyte-derived RNA (cDNA) of S17 with the heterozygous c.505-1G>A variant and two controls (C5, C6) using a primer pair targeting exons 6-9 (expected size: 264 bp). (B) Partial sequence electropherogram (reverse complement sequence shown) of directly sequenced RT-PCR products from S17 amplified with exon 6-9 primer pair. The reverse complement sequence spans the exon 8-7 junction and shows two overlapping sequences corresponding to exon 6 (upper sequence) and exon 7 (lower sequence). (C) Partial sequence electropherograms of cloned RT-PCR products. The upper panel shows the exon 6-7 junction of the *WDHD1* wild-type transcript, whereas the lower panel demonstrates skipping of exon 7 in *WDHD1* mRNAs of S17's leukocytes [r.505_600del; p.(Thr169_Lys200del)]. (D) Agarose gel showing RT-PCR amplicons generated from leukocyte-derived RNA (cDNA) of S17 with the heterozygous c.1342-6T>A variant and two controls (C5, C6) using a primer pair targeting exons 12-14 (expected size: 427 bp). The amplicons that appear slightly larger than the wild-type bands in S17 (see A and D) most likely represent heteroduplexes formed by two non-complementary strands, such as one strand of the wild-type (larger) amplicon and one strand of the smaller amplicon. (E) Partial sequence electropherogram (reverse complement sequence shown) of directly sequenced RT-PCR products from S17 amplified with exon 12-14 primer pair. The reverse complement sequence spans the exon 14-13 junction and shows two overlapping sequences corresponding to exon 12 (upper sequence) and exon 13 (lower sequence). (F) Partial sequence electropherograms of cloned RT-PCR products. The upper panel shows the exon 12-13 junction of the *WDHD1* wild-type transcript, whereas the lower panel demonstrates skipping of exon 13 in *WDHD1* mRNAs of S17's leukocytes [r.1342_1526del; p.(Val448Glnfs*8)].

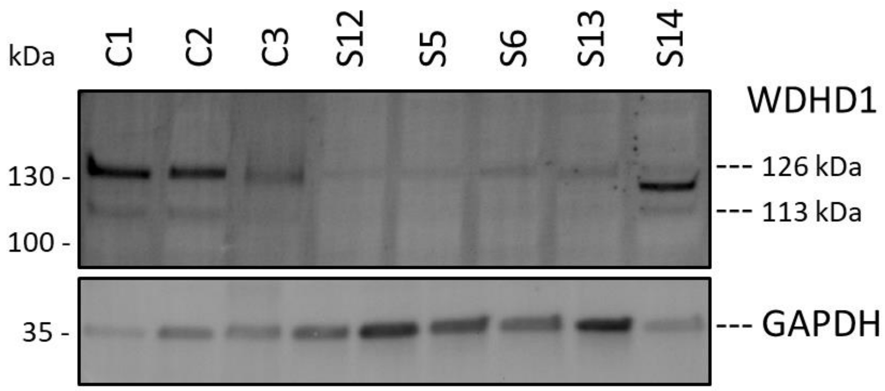

Figure S8. Long-exposure immunoblot showing WDHD1 protein levels in control- and subject-derived fibroblasts.

Representative immunoblot of fibroblast lysates from S12, S5, S6, S13, and S14 and three controls is shown. The blot was exposed for a longer duration to enhance visualization of faint bands. Levels of endogenous WDHD1 isoforms of 126 kDa and 113 kDa, as well as the C-terminally truncated proteoform in S14 cells, were assessed using the indicated antibodies, with GAPDH serving as a loading control.

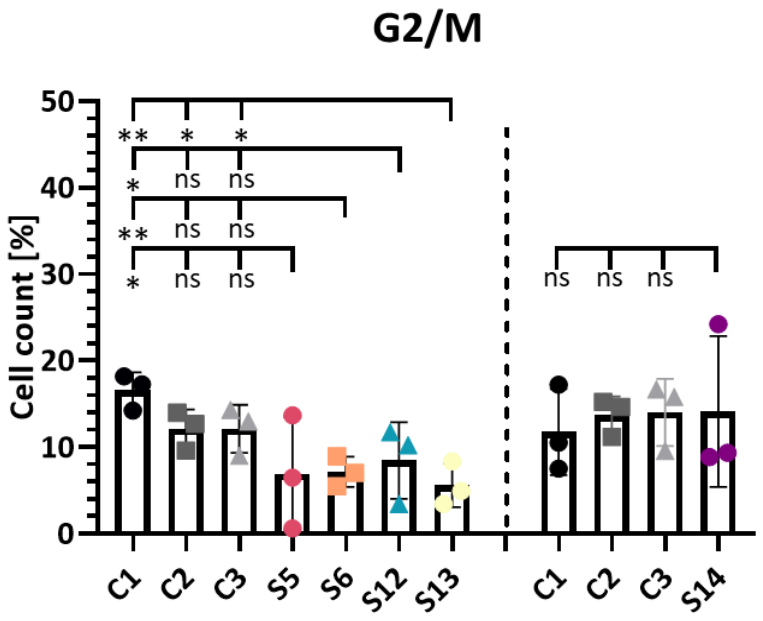

Figure S9. Quantification of control and subject cells in the G2/M phase.

Quantification of control and subject cells in the G2/M phase, derived from data shown in figure 3C. The percentage of cells in the G2/M phase is shown as the mean ± SD of three independent experiments. S14 fibroblasts were analyzed in a separate experiment with their own set of control cells. Statistical significance was determined by one-way ANOVA followed by Dunnett's multiple comparisons test. C1–C3, control fibroblasts; ns, not significant; S5, S6, S12-S14, subject fibroblasts. *$P \leq 0.05$; **$P \leq 0.01$.

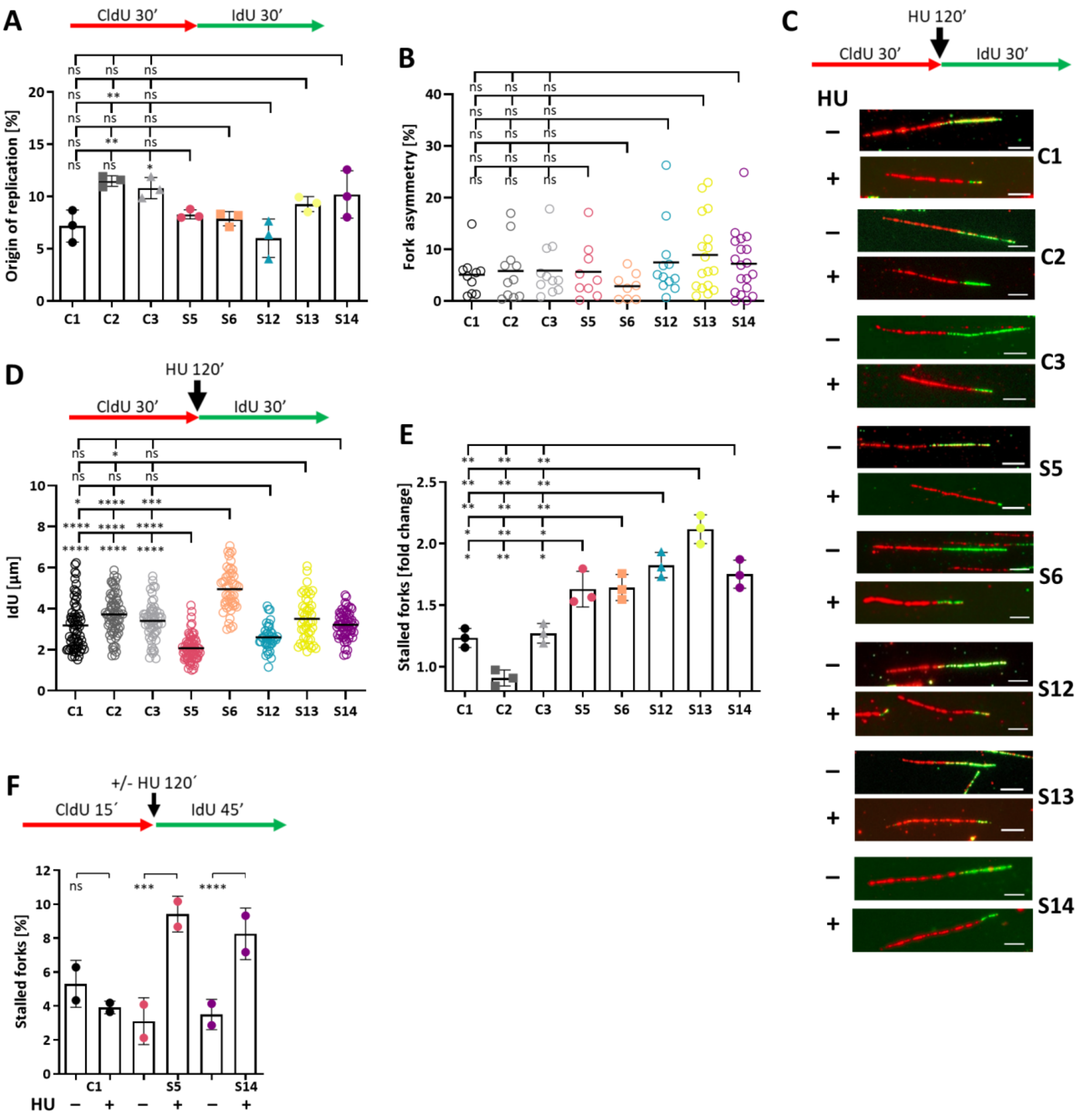

Figure S10. Analysis of origin of replication (ORI) and fork asymmetry under untreated conditions and of CldU length and stalled forks under hydroxyurea (HU)-treated conditions in subject and control fibroblasts.

(A) Treatment scheme of the DNA fiber assay with CldU and IdU labelling of cells for 30 minutes each, followed by immunofluorescence detection (CldU – red, IdU – green, top). Quantification of ORI in fibroblasts from S5, S6, and S12-S14, and three controls (bottom). The proportions of ORI were quantified relative to actively replicating forks and are presented as percentages. A minimum of 50 structures per experiment were analyzed for each cell line. The mean ± SD of three independent experiments is shown. (B) Analysis of replication fork symmetry was performed in fibroblasts from S5, S6, S12-S14, and three controls. Lengths of the bidirectional second tracks originating from an ORI were measured. The difference in length between each pair of second tracks was normalized to the total fiber length. In fibroblasts from C1-C3, the mean difference was 5.5% and determined as threshold for fork symmetry. Each data point represents the percentage of asymmetric replication forks in a single

image with >20 replication structures from three independent experiments. The black line indicates the mean. (C) Representative DNA fiber images of fibroblasts from C1-C3, S5, S6, and S12-S14 with (+) and without (-) HU treatment. Scale bar, 5 µm. (D) Scheme illustrates CldU and IdU labelling of cells for 30 minutes each, with a 2-hour incubation with HU in between to halt DNA replication (top). Effect of HU treatment on the length of IdU tracks in fibroblasts from S5, S6, S12-S14, and three controls (bottom). Individual data points obtained from three independent experiments are shown, with the mean indicated by the black line. (E) Effect of HU treatment on fork stalling in fibroblasts from S5, S6, S12-S14, and three controls after normalization to the percentage of stalled forks in untreated cells. Quantification of individual data sets, expressed as fold change relative to the respective untreated cell line. (F) Scheme illustrates CldU and IdU labelling of cells for 15 and 45 minutes, respectively, with a 2-hour incubation with (+) or without (-) HU in between to halt DNA replication (top). The proportions of stalled replication forks were quantified in fibroblasts from C1, S5, and S14 relative to actively replicating forks and are presented as percentages (bottom). A minimum of 50 structures per experiment was analyzed for each cell line in two independent experiments. Each data point represents the mean of one experiment. Statistical significance was determined by one-way ANOVA followed by Dunnett's multiple comparisons test (A-D) or student's t-test (F). C1-C3, control fibroblasts; ns, not significant; S5, S6, S12-S14, subject fibroblasts. *$P \leq 0.05$; **$P \leq 0.01$; ***$P \leq 0.001$; ****$P \leq 0.0001$.

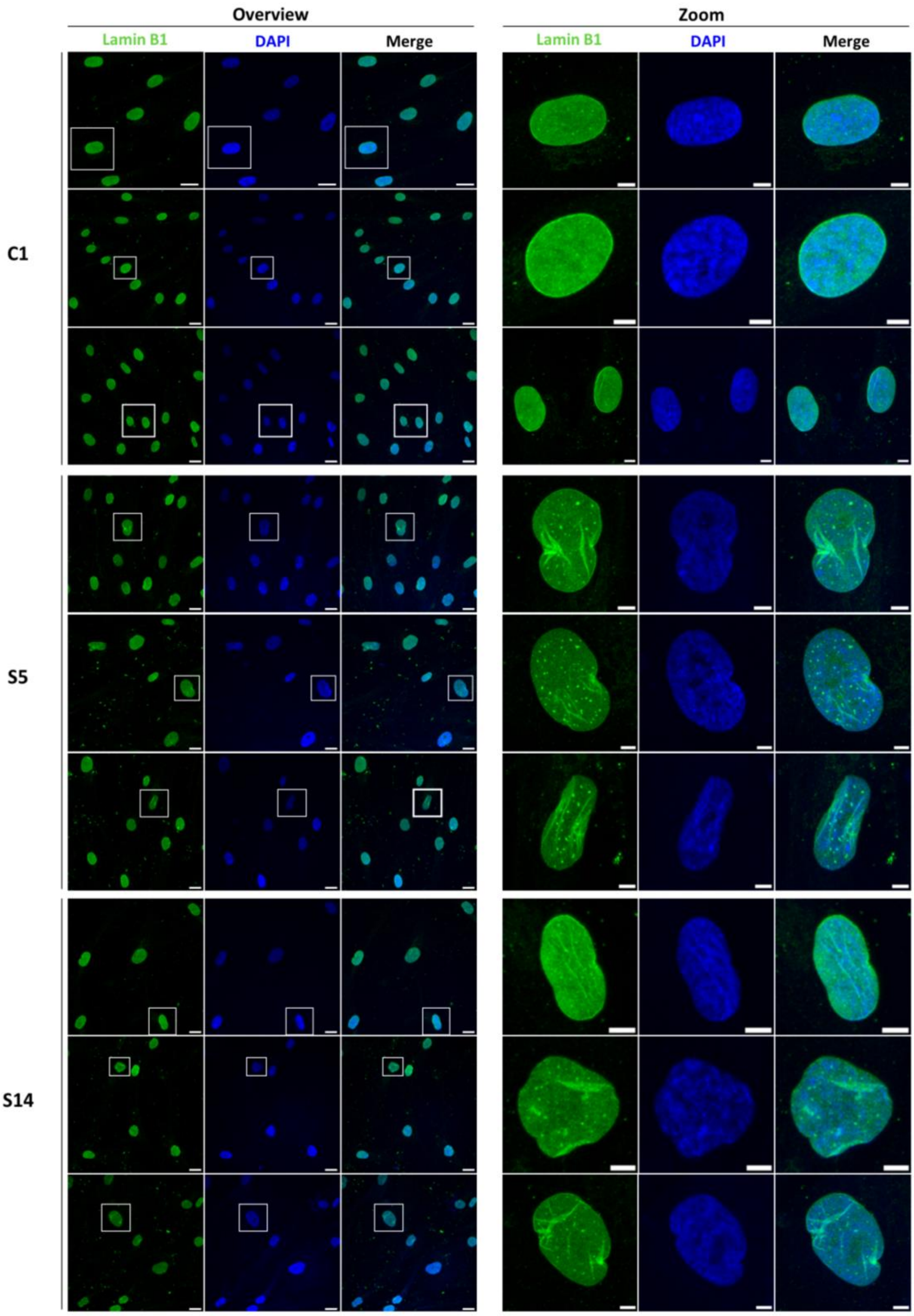

Figure S11. Altered lamina morphology in fibroblasts with biallelic *WDHD1* variants.

Immunofluorescence analysis of fibroblasts from control 1 (C1), S5, and S14. Cells were stained with an anti-Lamin B1 antibody, followed by an Alexa Fluor 488-conjugated secondary antibody (green) and DAPI (blue). Representative overview images are shown on the left. Scale bar, 20 µm. Exemplary zoom images on the right show aberrant lamina structures and distinct lamin B1-positive foci in cells from S5 and S14. Scale bar, 5 µm. S5, S14, subject fibroblasts.

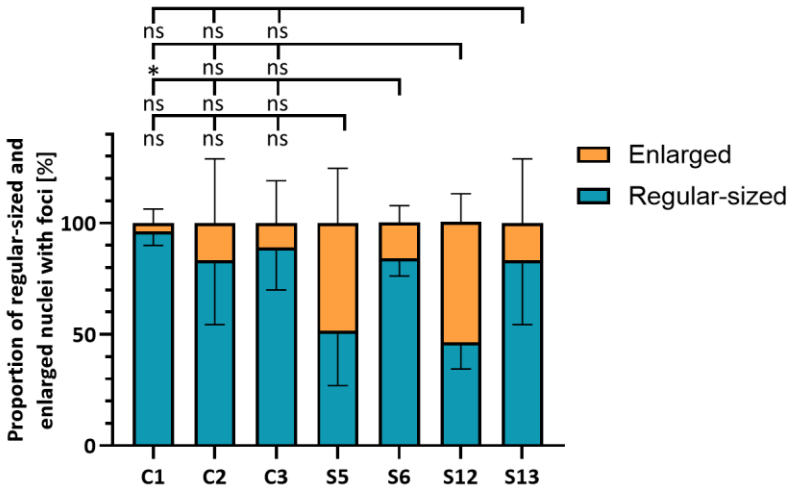

Figure S12. Proportion of regular-sized and enlarged nuclei containing ɣH2AX foci in control and subject cells.

Nuclei positive for γH2AX foci were categorized as regular-sized or enlarged, and the relative proportions of cells in each category were determined. The mean ± SD of three independent experiments in control and subject cells is shown. Statistical significance was determined by one-way ANOVA followed by Dunnett's multiple comparisons test. C1-C3, control fibroblasts; ns, not significant; S5, S6, S12, S13, subject fibroblasts. **P* ≤ 0.05.

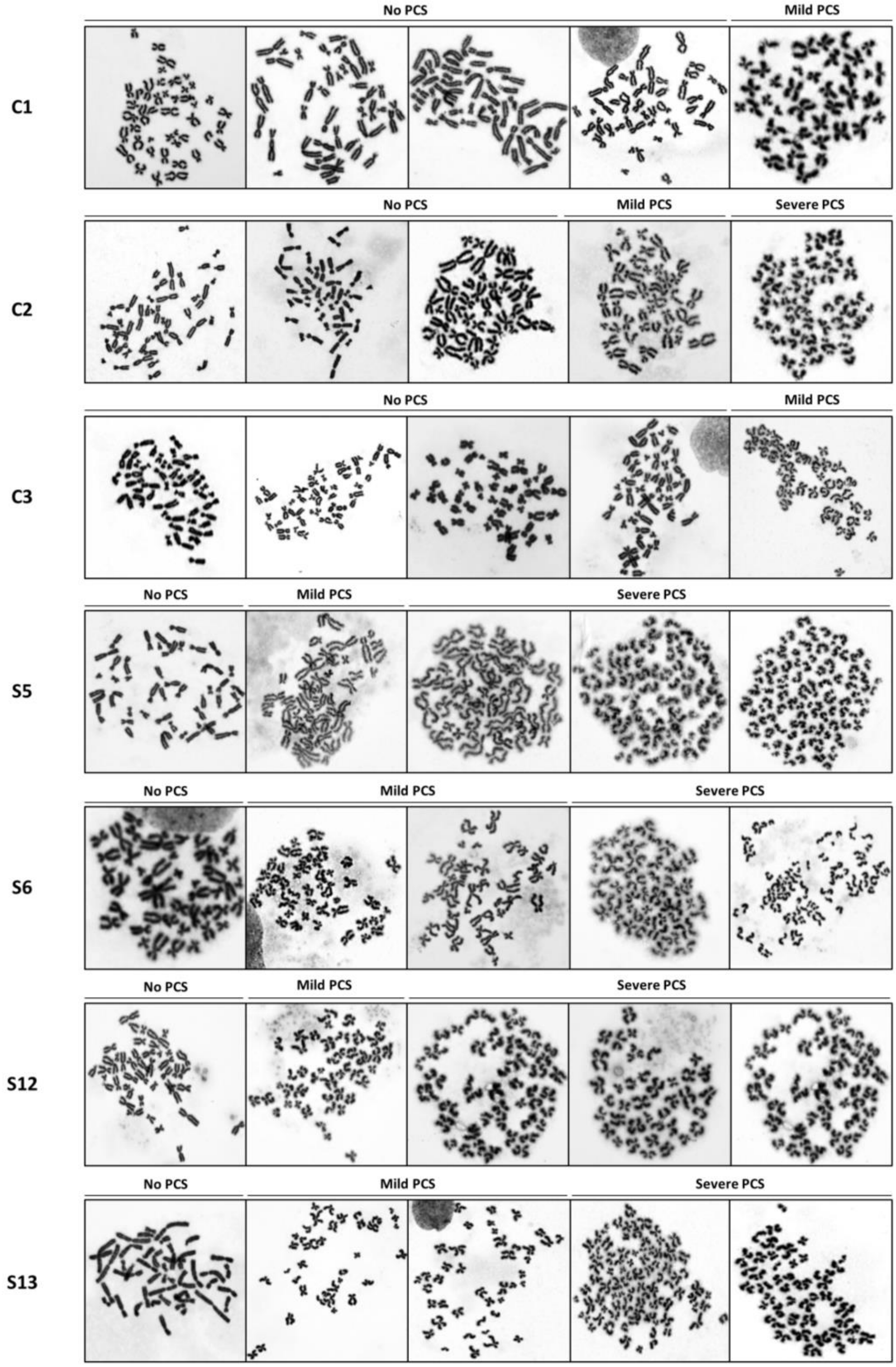

Figure S13. Analysis of premature chromatid separation (PCS) in fibroblasts with biallelic *WDHD1* variants.

Fibroblasts were arrested in mitosis, and metaphase spreads were prepared. Each metaphase (i.e. the complement of chromosomes from one cell) was classified as showing no PCS, mild PCS (>1 chromosome showing two separate chromatids with split centromere), or severe PCS (>50% of chromosomes showing two separate chromatids with split centromere). Representative images of each category are shown. C1-C3, control fibroblasts; S5, S6, S12, S13, subject fibroblasts.

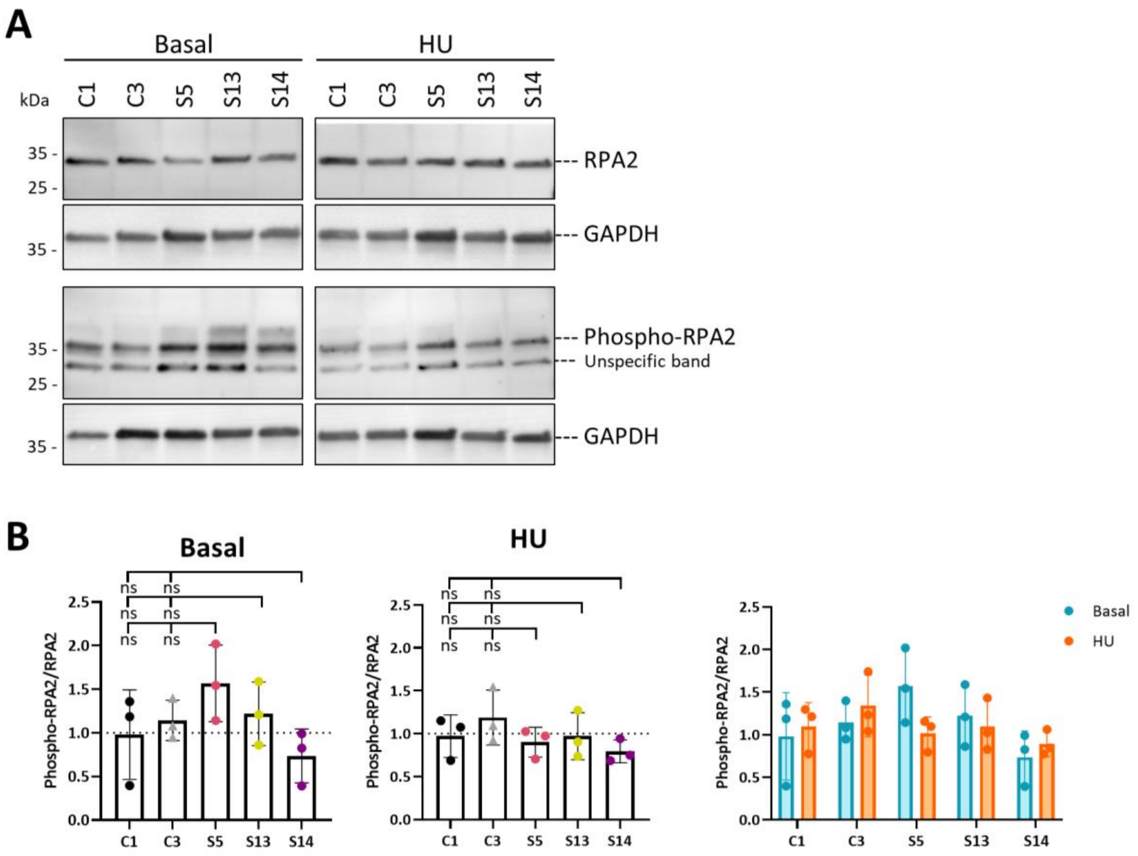

Figure S14. Determination of RPA2 and phospho-RPA2 levels in control and subject cells under basal and hydroxyurea-treated conditions.

(A) Representative immunoblots of fibroblast lysates from S5, S13, and S14 and controls under basal conditions (left) and following treatment with 2 mM hydroxyurea (HU) for 4 h (right). RPA2 and phospho-Ser4/8-RPA2 were detected using the indicated antibodies. An unspecific band is visible below the phospho-RPA2 band. GAPDH served as loading control for each blot. (B) Quantification of phospho-RPA2 relative to total RPA2. Band intensities of phospho-RPA2 and total RPA2 were first normalized to the corresponding GAPDH signal on each blot. The GAPDH-normalized phospho-RPA2 to RPA2 ratio is shown for subject and control cells cultivated under basal (left) and HU-treated (middle) conditions and is expressed relative to the mean of control cells. In the grouped analysis (right), values from HU-treated cells were normalized to the mean of control cells cultivated under basal conditions. The mean ± SD of three independent experiments is shown. Statistical significance was determined by one-way ANOVA followed by Dunnett's multiple comparisons test. C1, C3, control fibroblasts; ns, not significant; S5, S13, S14, subject fibroblasts.

Supplemental tables:

Table S1. Clinical characteristics of subjects with biallelic *WDHD1* variants

	Family 1	Family 2		Family 3		Family 4	Family 5	Family 6		Family 7
	**Subject 1**	**Subject 2**	**Subject 3**	**Subject 4**	**Subject 5**	**Subject 6**	**Subject 7**	**Subject 8**	**Subject 9**	**Subject 10**
**Sex**	Male	Male	Female	Male	Female	Male	Female	Male	Female	Male
**Age at last examination**	24 wk 5 d (gestation)	22 wk 5 d (gestation)	26 wk 3 d (gestation)	27 wk 3 d (gestation)	25 d	9 d	6 wk	7 wk	15 wk	2 d
**Alive/Dead**	Termination of pregnancy	Prenatal death	Prenatal death	Prenatal death	Died at 35 d	Died at 9 d	Died at 6 wk	Died at 8 wk	Died at 15 wk	Died at 2 d
**Consanguinity**	Yes	+	+	Unknown	Unknown	+	+	+	+	+
**Genotype**										
***WDHD1* variant** (NM_007086.4/ NP_009017.1)	c.1117C>T; p.(Arg373*) homozygous	c.1769-1G>C homozygous	c.1769-1G>C homozygous	c.1769-1G>C homozygous	c.1769-1G>C homozygous	c.1769-1G>C homozygous	c.1769-1G>C homozygous	c.1769-1G>C homozygous	c.1769-1G>C homozygous	c.2971G>T; p.(Glu991*) homozygous
**Chromosome position hg38**	Chr14-54995639	Chr14-54984861	Chr14-54984861	Chr14-54984861	Chr14-54984861	Chr14-54984861	Chr14-54984861	Chr14-54984861	Chr14-54984861	Chr14-54955640
**Phenotype**										
**Prenatal & neonatal features**	**Subject 1**	**Subject 2**	**Subject 3**	**Subject 4**	**Subject 5**	**Subject 6**	**Subject 7**	**Subject 8**	**Subject 9**	**Subject 10**
**Abnormalities in pregnancy**	IUGR, decreased fetal movements, borderline oligohydramnios	IUGR, oligohydramnios	IUGR	IUGR, oligohydramnios, abnormal umbilical artery doppler waveform during pregnancy	IUGR, oligohydramnios, abnormal umbilical artery doppler waveform during pregnancy	IUGR	IUGR	IUGR	IUGR	IUGR, oligohydramnios, decreased fetal movement
**Abnormal fetal morphology**	Microcephaly	Microcephaly	Microcephaly, cloverleaf skull	Microcephaly	–	Thickened nuchal skin fold	–	Microcephaly	Microcephaly	Retrognathia, bilateral dilatation of the renal pelvis, hyperechogenic kidneys
**Gestational age at birth**	–	–	–	–	33 wk 5 d	39 wk 3 d	37 wk 4 d	38 wk	35 wk 1 d	38 wk 2 d
**Length at birth**	Not reported	33 cm (– 0.99 z)	Not reported	Not reported	33 cm (– 4.00 z)	42 cm (– 4.42 z)	39 cm (– 4.71 z)	46 cm (– 2.22 z)	42 cm (– 1.94 z)	Not reported
**Weight at birth**	510 g (– 3.0 z)	530 g (– 0.78 z)	Not reported	542 g (– 2.10 z)	970 g (– 2.85 z)	1475 g (– 4.74 z)	1450 g (– 3.83 z)	2235 g (– 2.47 z)	1665 g (– 1.98 z)	1100 g (– 5.19 z)
**Occipitofrontal circumference (OFC) at birth**	18.5 cm (– 3.0 z)	Not reported	Not reported	21 cm (– 4.30 z)	24 cm (– 4.20 z)	28.5 cm (– 5.33 z)	28.5 cm (– 4.20 z)	29.5 cm (– 3.86 z)	28 cm (– 2.97 z)	Not reported
**Height at last examination**	Not applicable	Not applicable	Not applicable	Not applicable	39.5 cm (– 5.10 z)	43 cm (– 3.87 z)	40 cm (– 6.17 z)	48 cm (– 3.68 z)	52 cm (– 3.19 z)	Not reported
**Weight at last examination**	Not applicable	Not applicable	Not applicable	Not applicable	2.4 kg (– 1.90 z)	2.2 kg (– 2.57 z)	1.7 kg (– 4.41 z)	2.8 kg (– 4.20 z)	4.3 kg (– 1.90 z)	Not reported
**OFC at last examination**	Not applicable	Not applicable	Not applicable	Not applicable	28.1 cm (– 7.80 z)	29.5 cm (– 5.70 z)	28 cm (– 12.12 z)	30 cm (– 8.14 z)	35 cm (– 5.41 z)	Not reported

Development	Subject 1	Subject 2	Subject 3	Subject 4	Subject 5	Subject 6	Subject 7	Subject 8	Subject 9	Subject 10
**Motor delay**	Not applicable	Not applicable	Not applicable	Not applicable	Not applicable	Not applicable	+	Not applicable	Not applicable	Not applicable
**Age at walking**	Not applicable	Not applicable	Not applicable	Not applicable	Not applicable	Not applicable	Not applicable	Not applicable	Not applicable	Not applicable
**Delayed speech and language development**	Not applicable	Not applicable	Not applicable	Not applicable	Not applicable	Not applicable	Not applicable	Not applicable	Not applicable	Not applicable
**Age at first words**	Not applicable	Not applicable	Not applicable	Not applicable	Not applicable	Not applicable	Not applicable	Not applicable	Not applicable	Not applicable
**Current number of words**	Not applicable	Not applicable	Not applicable	Not applicable	Not applicable	Not applicable	Not applicable	Not applicable	Not applicable	Not applicable
**Global developmental delay/intellectual disability**	Not applicable	Not applicable	Not applicable	Not applicable	Not applicable	Not applicable	Not applicable	Not applicable	Not applicable	Not applicable
**EEG abnormality**	Not applicable	Not applicable	Not applicable	Not applicable	Not done	Not done	Intermittent flattening of the brain wave pattern	Seizure	Seizure	Not done
**Neurological features**	**Subject 1**	**Subject 2**	**Subject 3**	**Subject 4**	**Subject 5**	**Subject 6**	**Subject 7**	**Subject 8**	**Subject 9**	**Subject 10**
**Abnormal muscle tone**	Not applicable	Not applicable	Not applicable	Not applicable	Generalized hypotonia	Neonatal hypotonia	Generalized hypotonia	Generalized hypotonia	Generalized hypotonia	Neonatal hypotonia
**Brain abnormalities**	Prenatal ultrasound: no brain abnormality found	Cavum septum pellucidum, cerebellar hypoplasia, dysplastic corpus callosum, enlarged fetal cisterna magna, small cerebral cortex	Abnormal lateral ventricle morphology, cerebral hypoplasia, lissencephaly	Prenatal MRI: Hypoplasia of the corpus callosum	Ultrasound: Hypoplasia of the corpus callosum, frontal pachygyria	Immature gyration, lateral ventricular asymmetry	Relatively immature gyration, narrow symmetrical ventricular system	Ultrasound normal	MRI: abnormal brain morphology, abnormal myelination, cerebral edema, abnormal cortical gyration (reduced)	Abnormal brain morphology
**Abnormality of the face**	**Subject 1**	**Subject 2**	**Subject 3**	**Subject 4**	**Subject 5**	**Subject 6**	**Subject 7**	**Subject 8**	**Subject 9**	**Subject 10**
**Face**	Not reported	Not reported	Not reported	Not reported	+	Not reported	+	Small face	Small face	Not reported
**Forehead**	Not reported	Not reported	Not reported	Not reported	Not reported	Not reported	Not reported	–	–	Not reported
**Ear**	Not reported	Not reported	Not reported	Not opened	Low-set ears, pointed ear	Low-set ears	–	–	–	Not reported
**Eye**	–	Microphthalmia	Not reported	Microphthalmia, downslanted palpebral fissures, short palpebral fissure, hypertelorism, epicanthus	Microphthalmia, down-slanted palpebral fissures, short palpebral fissure, hypertelorism, epicanthus	Deeply set eyes	Not reported	Microphthalmia	Microphthalmia	Not reported
**Nose**	Not reported	Not reported	Wide nose	Not reported	Bulbous nose, wide nose	Not reported	Not reported	–	–	Not reported
**Mouth**	–	Not reported	Not reported	Cleft palate, furrowed tongue	Narrow mouth	Not reported	Not reported	–	–	Cleft palate
**Chin**	Not reported	Not reported	Not reported	Mild retrognathia	Mild retrognathia	Short chin	Not reported	Mild retrognathia	–	Retrognathia
**Miscellaneous**	**Subject 1**	**Subject 2**	**Subject 3**	**Subject 4**	**Subject 5**	**Subject 6**	**Subject 7**	**Subject 8**	**Subject 9**	**Subject 10**
**Abnormal hair morphology**	Not applicable	Not applicable	Not applicable	Not applicable	–	Not reported	Not reported	Not reported	Not reported	–
**Hearing impairment**	Not applicable	Not applicable	Not applicable	Not applicable	Not reported	Not reported	Not reported	Not reported	Not reported	Not reported
**Visual impairment**	Not applicable	Not applicable	Not applicable	Not applicable	Cataract (bilateral), abnormal retinal vascular morphology, microcornea	Not reported	Optic atrophy	Cataract, retinal dystrophy, iris malposition	Retinal dystrophy	Not reported

**Abnormal heart morphology**	Hypoplastic left heart, abnormal heart morphology	Ventricular septal defect	Small apical ventricular septal defect	Hypoplastic heart, abnormal anatomic location of the heart (left rotated), left ventricular dilatation	Patent foramen ovale	Patent ductus arteriosus	Minimal pericardial effusion	–	–	Abnormal heart morphology
**Abnormality of the respiratory system**	Not applicable	Not applicable	Not applicable	Not applicable	Respiratory insufficiency, highflow support	Respiratory insufficiency, highflow support	Neonatal respiratory distress,<br>respiratory distress	Respiratory insufficiency (mech. ventilated)	Respiratory insufficiency (mech. ventilated)	Not reported
**Abnormality of the endocrine system**	–	Not reported	Not reported	Hypoplasia of the thymus	Hyperinsulinemia, congenital hypothyroidism	–	Hyperinsulinemia	–	–	Not reported
**Abnormality of the immune system**	Not reported	Not reported	Not reported	Hypoplastic spleen	Recurrent infections	Not reported	Anti-herpes simplex antibody positivity, mild leukopenia	–	–	Not reported
**Abnormality of the digestive system**	Not reported	–	Abnormality of the gastrointestinal tract	Abnormal intestine morphology (malrotated)	Abnormal intestine morphology (pathological thickening of the mucosa),<br>mild ascites,<br>rectal prolapse	–	Ascites	Feeding difficulties, tube feeding	Feeding difficulties, tube feeding	Not reported
**Abnormality of the liver**	Not reported	Not reported	Not reported	Hepatic fibrosis, hepatic cysts	Decreased liver function, abnormal liver morphology (inhomogeneous), hepatic arterioportal fistulas, hypoplastic, intrahepatic gallbladder	Acute hepatic failure, cirrhosis, elevated hepatic iron concentration, giant cell hepatitis, cholestasis, suspected biliary atresia	Cirrhosis, acute hepatic failure, elevated hepatic iron concentration, giant cell hepatitis,<br>Hepatosplenomegaly	Cirrhosis (biopsy), acute hepatic failure, elevated hepatic iron concentration	Cirrhosis (biopsy at 6 wks), acute hepatic failure, elevated hepatic iron concentration	–
**Abnormality of metabolism/ homeostasis**	Not applicable	Not applicable	Not applicable	Not<br>applicable	Increased circulating lactate concentration, hypoglycemia	Not reported	Hypoglycemia, abnormality of amino acid metabolism, hyperammonemia	Hypoglycemia	Hypoglycemia	Not reported
**Abnormality of the genitourinary system**	Ambiguous genitalia, male	Not reported	Hyperechogenic right kidney	Enlarged kidneys	Vesicoureteral reflux (right), right-sided hydroureter, aplasia of the uterus	–	–	–	–	Ambiguous genitalia, male
**Abnormal skeletal morphology**	Not reported	Not reported	Dolichocephaly	Sandal gap	Hip dislocation (bilateral)	–	Skeletal dysplasia	–	–	Short long bones
**Abnormality of limbs**	Not reported	Not reported	Not reported	2-3 toe syndactyly (right foot)	2-4 toe syndactyly	Not reported	Abnormal foot morphology (hook feet)	2-3 toe syndactyly (left foot)	–	Malposition of feet with external rotation
**Other abnormalities**	Not reported	Not reported	Not reported	–	Hypoesthesia	Not reported	Mild thrombocytopenia,<br>short stature	–	–	Not reported

Table continues on next pages.

	Family 8	Family 9	Family 10	Family 11	Family 12	Family 13	Family 14
	**Subject 11**	**Subject 12**	**Subject 13**	**Subject 14**	**Subject 15**	**Subject 16**	**Subject 17**
**Sex**	Male	Female	Female	Male	Male	Female	Male
**Age at last examination**	5 y 11 m	8 y 10 m	10 y 7 m	3 y 1 m	11 y 9 m	16 y 9 m	9 y
**Alive/Dead**	Alive	Alive	Alive	Alive	Alive	Alive	Alive
**Consanguinity**	–	–	–	–	Not reported	–	–
**Genotype**							
***WDHD1* variant** (NM_007086.4/NP_009017.1)	c.1769-1G>C homozygous	c.1769-1G>C homozygous	c.1341+5_1341+6inv, c.3190-1G>A compound heterozygous	c.1341+5_1341+6inv, c.3157C>T; p.(Arg1053*) compound heterozygous	c.1287del; p.(Gln430Lysfs*37), c.2339G>A; p.(Arg780His) compound heterozygous	c.1341+5G>T, c.2531+1G>A compound heterozygous	c.505-1G>A, c.1342-6T>A compound heterozygous
**Chromosome position hg38**	Chr14-54984861	Chr14-54984861	Chr14-54991207-54991208, chr14-54941691	Chr14-54991207-54991208, chr14-54944364	Chr14-54991266, chr14-54963144	Chr14-54962951 Chr14-54991208	Chr14-55007376 Chr14-54989218
**Phenotype**							
**Prenatal & neonatal features**	**Subject 11**	**Subject 12**	**Subject 13**	**Subject 14**	**Subject 15**	**Subject 16**	**Subject 17**
**Abnormalities in pregnancy**	Likely IUGR, oligohydramnios	IUGR, oligohydramnios	IUGR, oligohydramnios, small placenta	IUGR, oligohydramnios, small placenta (281 g), abnormal umbilical cord blood vessel morphology	IUGR	IUGR	IUGR
**Abnormal fetal morphology**	Limited antenatal care	Not reported	Microcephaly, webbed neck, wide intermamillary distance, aplasia/hypoplasia of the patella	Microcephaly	Microcephaly	Microcephaly	Small for gestational age
**Gestational age at birth**	34 wk	27 wk 5 d	31 wk	34 wk 1 d	39 wk	37 wk	33 wk 6 d
**Length at birth**	Not reported	35 cm (– 0.36 z)	33 cm (– 2.30 z)	43.5 cm (– 1.03 z)	44 cm (– 3.20 z)	40 cm (– 3.96 z)	39 cm (– 2.20 z)
**Weight at birth**	1015 g (– 3.12 z)	1160 g (+ 0.59 z)	935 g (– 1.75 z)	1490 g (– 2.09 z)	2810 g (– 1.40 z)	1950 g (– 2.35 z)	1380 g (– 2.03 z)
**Occipitofrontal circumference (OFC) at birth**	Not reported	26 cm (+ 0.03 z)	25 cm (– 1.95 z)	28 cm (– 2.57 z)	Not reported	Not reported	29 cm (– 1.34 z)
**Height at last examination**	88.9 cm (– 5.71 z)	93 cm (– 6.65 z)	109 cm (– 4.10 z)	73.5 cm (– 5.7 z)	123.5 cm (– 3.30 z)	129.8 cm (– 5.69 z)	115.8 cm (– 3.11 z)
**Weight at last examination**	8.9 kg (– 9.39 z)	10.6 kg (– 8.82 z)	15.9 kg (– 4.10 z)	7.45 kg (– 5.34 z)	23 kg (– 3.39 z)	27.2 kg (– 6.82 z)	20.5 kg (– 2.65 z)
**OFC at last examination**	41.6 cm (– 8.13 z)	42 cm (– 10.72 z)	48.2 cm (– 3.58 z)	45.6 cm (– 3.2 z)	46.5 cm (– 4.60 z)	50 cm (– 4.55 z)	47 cm (– 2.9 z)
**Development**	**Subject 11**	**Subject 12**	**Subject 13**	**Subject 14**	**Subject 15**	**Subject 16**	**Subject 17**
**Motor delay**	+ Mild gross motor delay, fine motor delay	+ Mild gross motor delay	+	+	+	–	+ Fine and gross motor delay
**Age at walking**	20 m	22 m	3 y	18 m	Not reported	14 m	22 m

**Delayed speech and language development**	+<br>Mild to moderate receptive and expressive speech delay, articulation difficulties	+<br>Mild to moderate receptive and expressive speech delay	+<br>Mild	+<br>Mild	+	–	Articulation difficulties
**Age at first words**	3 y	2 y 5 m<br>Single words	2 y	2 y	3 y	10 m	15 m
**Current number of words**	Speaks in short sentences, both in Kurdish and German	8 y<br>Speaks in short sentences, both in Kurdish and German	Normal number of words	Speaks words, no sentences	Speaks in short sentences	Completed basic education	Speaks in sentences, in mainstream education
**Global developmental delay/intellectual disability**	+<br>Mild to moderate	+<br>Degree not tested	+<br>Mild	+<br>Mild speech receptive, moderate expressive, short attention span	+<br>Mild	–	+<br>Developmental quotient 1st percentile
**EEG abnormality**	Not done<br>(no clinical concern for seizures)	–	Not done	–	Not done	Not done	Not done
**Neurological features**	**Subject 11**	**Subject 12**	**Subject 13**	**Subject 14**	**Subject 15**	**Subject 16**	**Subject 17**
**Abnormal muscle tone**	–	–	Generalized hypotonia	–	Neonatal hypotonia	Not reported	Generalized hypotonia
**Brain abnormalities**	Choroid fissure cysts	Lateral ventricular asymmetry, aqueductal stenosis	MRI normal (shortly after birth, not repeated)	Ultrasound normal (after birth)	MRI: left temporal lobe cyst	MRI: pituitary hypoplasia without other abnormalities	MRI: Supratentorial white matter signal abnormality most prominently involving the frontal lobes and temporal poles
**Abnormality of the face**	**Subject 11**	**Subject 12**	**Subject 13**	**Subject 14**	**Subject 15**	**Subject 16**	**Subject 17**
**Face**	Small face, low posterior hairline	Small face	Small face	Small face	Narrow face	Small face	–
**Forehead**	–	Small forehead	High forehead	High forehead	High forehead	High forehead	High Forehead
**Ear**	–	–	Low-set ears	–	Not reported	–	–
**Eye**	Short palpebral fissure, long eyelashes	Microphthalmia (left eye), small upslanted palpebral fissures, synophrys, hypertelorism	Hypertelorism, downslanted palpebral fissures	Short palpebral fissures	Microphthalmia, deeply set eyes, narrow palpebral fissure, microcornea,<br>strabismus	Puffy eyes, myopia	Hypertelorism, blepharophimosis, strabismus, epicanthus
**Nose**	Wide nasal bridge	Bulbous nose	Bulbous nose	Bulbous tip of the nose	–	Bulbous nose	Broad nose
**Mouth**	Thin vermilion border, carious teeth	Delayed eruption of primary teeth	High palate	Normal palate, thin upper lip, late dentition (first teeth 16 months)	–	Thin vermilion border,<br>high palate	Thin vermillion border, carious teeth
**Chin**	Pointed chin	Pointed chin	Mild retrognathia, pointed chin	Mild retroganthia	Pointed chin	Retrognathia	Pointed chin
**Miscellaneous**	**Subject 11**	**Subject 12**	**Subject 13**	**Subject 14**	**Subject 15**	**Subject 16**	**Subject 17**
**Abnormal hair morphology**	Hypertrichosis	–	–	–	–	–	Sparse hair
**Hearing impairment**	Not reported	–	–	–	Stenosis of the external auditory canal	–	–
**Visual impairment**	Myopic astigmatism, visual acuity appropriate for age, requires prescription glasses	Exotropia, microcornea, secondary cataract with synechiae, exudative retinal detachment (right)	Optic atrophy, glaucoma, abnormality of the fovea	Optic atrophy	Optic atrophy	–	Myopia

**Abnormal heart morphology**	Functionally bicuspid aortic valve, mildly dilated ascending aorta	–	Patent foramen ovale	–	Not reported	–	–
**Abnormality of the respiratory system**	Neonatal respiratory distress	–	Laryngotracheal stenosis	Neonatal respiratory distress (surfactant, extubation after 5h, CPAP 3 days)	Neonatal respiratory distress	–	Laryngotracheomalacia
**Abnormality of the endocrine system**	–	Congenital hypothyroidism	Anterior hypopituitarism, hy-pogonadotropic hypogonadism, growth hormone deficiency, treated with growth hormone since the age of 4 months	Low IGF1, treated with growth hormone from 21 month	Central hypothyroidism, central adrenal insufficiency, growth hormone deficiency, treated with growth hormone since the age of 3 y	Congenital hypothyroidism, treated with rhGH from 2 to 9 months without effective re-sponse (normal GH secretion)	–
**Abnormality of the immune system**	Recurrent infections	Recurrent infections of the gas-trointestinal tract	Not reported	Not reported	–	–	–
**Abnormality of the digestive system**	Feeding difficulties, tube feed-ing, right inguinal hernia	Feeding difficulties, tube feed-ing, recurrent vomiting, percu-taneous endoscopic jejunos-tomy (PEJ)	Feeding difficulties	Feeding difficulties	–	–	–
**Abnormality of the liver**	Abnormal liver morphology, suggested focal hepatic focal nodular hyperplasia, slightly raised liver enzymes, NB re-solved on latest scan and blood aged 6 years	Intermittently elevated liver en-zymes	Not reported	–	–	–	–
**Abnormality of metabolism/ homeostasis**	Very slightly raised ammonia	Not reported	Neonatal hypoglycemia	Neonatal hypoglycemia	Hypoglycemia	–	–
**Abnormality of the genitouri-nary system**	Cryptorchidism	–	–	Small genitalia	Bilateral cryptorchidism	–	Glandular hypospadias, bilat-eral cryptorchidism, small scro-tum, chordee
**Abnormal skeletal morphology**	Slender long bones, moderate thoracic kyphosis with bullet shaped appearance to thoracic vertebrae but normal inter-pedicular distances	Hip dislocation (bilateral), marked delay in bone age, ver-tebral fusion (partial C5-C7)	Congenital hip dislocation, ab-normality of skeletal matura-tion, epiphyseal dysplasia	Congenital dislocation of right hip, abnormality of skeletal maturation	Hip dislocation (bilateral)	Hip dislocation (bilateral)	Hip dislocation (bilateral), slen-der long bones, delayed bone age, possible epiphyseal dyspla-sia
**Abnormality of limbs**	Clinodactyly of the 5th finger	–	Aplasia/hypoplasia of the pa-tella	Small patella	–	–	Lower limb asymmetry
**Other abnormalities**	High pitched voice, dental car-ies, demineralised teeth	High pitched voice	High pitched voice	High pitched voice	High pitched voice, microdontia	High pitched voice	High pitched voice

+, present; –, absent; d, days; EEG, electroencephalography; IUGR, intrauterine growth retardation; m, months; MRI, magnetic resonance imaging; wk, weeks; y, year(s).

Table S2. Sequence of oligonucleotides used in this work.

WDHD1 primer sequences for variant validation and/or segregation analysis			
**Template**	**Exon/ Intron**	**Direction**	**Sequence (5´ → 3´)**
Genomic DNA (subject 5)	Intron 14	Forward	CCTCAGTTGGATGGGTTATTCA
	Exon 15	Reverse	CCTTGTAAGAGGAAGAGGGTCA
Genomic DNA	Exon 12	Forward	GTCAAGAAGGCAGCATTCAC
Genomic DNA	Intron 12 (2)	Reverse	GCTCCAGGCTACTTAATCAAAG
Genomic DNA	Intron 25	Forward	GTGTAAATCTTTAATGTGCTCTGC
Genomic DNA	Exon 26	Reverse	CCTGCTTAAATGCAAAAGCTG
Genomic DNA	Intron 24	Forward	GAATTAACAAGTGTAACTGTGTCTTCC
Genomic DNA	Intron 25	Reverse	ACCATACCCAGCCAAAAGG
**Primer sequences for *WDHD1* transcript analysis**			
**Template**	**Exon/ Intron**	**Direction**	**Sequence (5´ → 3´)**
Complementary DNA (cDNA)	Exon 13	Forward	TCCATGATACCTCCATACACCA
cDNA	Exon 15	Reverse	CAGCTGAAAACCCAATCCAT
cDNA	Exon 16	Reverse	ATGGATACCAACCACCCAGTAG
cDNA	Exon 25	Forward	AAGACCGGGTTCCAGATGTG
cDNA	Exon 26	Reverse	TCCTGCTTAAATGCAAAAGCTG
cDNA	Exon 11	Forward	GTCACATCCTAGAAGATGATG
cDNA	Exon 13	Reverse	GCAGCGAATAATTCCAATAGAGTTCC
cDNA	Intron 12	Forward	GTAATCCTTGGATCTACACTTAAC
cDNA	3´UTR	Reverse	TGCATTTGGAGGCAGAGTAATC
cDNA	Intron 12-Exon 13	Reverse	CCACACTAAGAAGTTAAGTGTAGATCC
cDNA	Exon 24	Forward	GCATCTGCAGCATCCTATTTCC
cDNA	Exon 6	Forward	GATGGATCTGTCAGAGTGTGG
cDNA	Exon 9	Reverse	ATATTGCCCACAGGGAGACC
cDNA	Exon 12	Forward	CAAAAGCCATTTCAGTCAGG
cDNA	Exon 14	Reverse	GTCCAGCAAGGCTGAATACC
**Primer sequences for colony PCR**			
**Template**	**Region**	**Direction**	**Sequence (5´ → 3´)**
pCR2.1 TOPO vector	T7	Forward	TAATACGACTCACTATAGGG
pCR2.1 TOPO vector	M13	Reverse	CAGGAAACAGCTATGAC

Table continues on next page.

Primer sequences for quantitative *WDHD1* transcript analysis			
**Template**	**Exon**	**Direction**	**Sequence (5´ → 3´)**
cDNA	13	Forward	TGATACCTCCATACACCATGCAAC
cDNA	14	Reverse	CACTCTTTGCTTGAATCCCAAGAAC
cDNA	15	Forward	GGTGACCCTCTTCCTCTTACAAG
cDNA	16	Reverse	CCAACCACCCAGTAGTGATCAG

Table S3: Number of cells analyzed for quantification of γH2AX foci and nuclear morphology in three independent experiments.

	**C1**	**C2**	**C3**	**S5**	**S6**	**S12**	**S13**
**n = 1**	63	52	45	47	37	73	55
**n = 2**	78	41	44	56	73	43	51
**n = 3**	61	71	103	23	90	33	47
**Total**	202	164	192	126[a]	200	149	153

[a]As S5 fibroblasts show a severe proliferation defect, the target number of at least 150 nuclei could not be reached for the analysis.

Supplemental acknowledgements

Regarding subject 10: In some cases, clinical genomic sequencing data was generated by a NATA-accredited laboratory and transferred to the UDN-Aus study for research analysis. This was made possible by NSW Health Pathology, Genomics Laboratory, Prince of Wales Hospital, Randwick, NSW, Australia and the Sydney Children's Hospitals Network and Rare Diseases NSW undiagnosed disease program GeneAdd.

Supplemental material and methods

Genetic analyses

Subject 1

Trio whole-exome sequencing (WES) was performed on DNA extracted from amniotic fluid of subject 1 and parents' peripheral blood using standard protocols. Libraries preparation was performed using a TWIST BioScience Library EF KIT (TWIST- Alliance) kit, with libraries analyzed on an Illumina NovaSeq 6000 Sequencing System (Illumina San Diego, CA) in paired-end mode. Reads were aligned to Human Genome Reference Sequence GRCh38, and single nucleotide and short insertion/deletion variants were identified using the DRAGEN Enrichment pipeline (v4.4.6) on the Illumina BaseSpace DRAGEN Server. Variant filtering, prioritisation, and reporting were performed using the Genomics Annotation and Interpretation Application (GAIA) in-house pipeline. Variants were filtered based on inheritance pattern, impact, frequency, zygosity and in silico pathogenicity scores. The data analysis pipeline is based on Gemini (v18) with annotation from VEP and dbNSFP. Copy number variants (CNV) were identified by three CNV callers: CoNIFER, DECoN, and XHMM.[1-3] CNVs were then filtered according to morbid gene content relevant to the referral phenotype and the number of callers identifying a potential CNV. Each variant was evaluated based on the available information from the following: databases (typically including, but not limited to, the latest public version of ClinVar, LSDBs and gnomAD), published literature, clinical correlation, segregation analysis, functional studies, and predicted functional or splicing impact using evolutionary conservation analysis and computational

tools. Variants were classified following the joint consensus recommendations of the American College of Medical Genetics and Genomics and the Association for Molecular Pathology for the interpretation of sequence variants[4] as well as integrating guidance from the best practice guidelines for variant interpretation published by the Association for Clinical Genomic Science (https://www.acgs.uk.com/quality/best-practice-guidelines/). Variants were reported according to HGVS nomenclature. The possibility of significant maternal cell contamination was excluded by comparative genotyping at seven informative, unlinked STR loci using the Promega PowerPlex 16HS Kit.

Subjects 2 and 3

Genomic DNA extraction from EDTA blood and amniocytes was carried out by using the QIAamp DNA Blood Mini Kit (Qiagen, Hilden, Germany) according to the manufacturer's protocol. DNA concentration was measured with the Qubit system (dsDNA Assay Kit; Thermo Fisher Scientific, Waltham, MA, USA). WES on DNA of subjects 2 and 3 and parents was performed by using an xGen Exome Research Panel v2, targeting 19,433 genes (Integrated DNA Technology, Coralville, IA, USA) according to the manufacturer's protocol. Genomic DNA was processed via hybrid capture, with target regions enriched and amplified by PCR using the Lotus DNA Library Prep Kit (Integrated DNA Technology). Massively parallel sequencing was performed on the NextSeq 2000 Sequencing System (Illumina, San Diego, CA, USA). WES quality control criteria required a minimum coverage of 25× across 100% of the targeted regions. Further details on the protocols used are available upon request.

Subjects 4 and 5

DNA was isolated from EDTA blood samples of subjects 4 and 5 and parents, fragmented to 350-400 bp, and quality-checked. Libraries were prepared with the Human Core Exome Kit (Twist Bioscience) and sequenced on an Illumina NovaSeq 6000 (2 × 100 bp paired-end), achieving >20× coverage for >95% of the RefSeq target region. Raw reads were demultiplexed and aligned to GRCh37 using BWA-Mem v0.7.11 (https://ieeexplore.ieee.org/document/8820962),[5] ensuring >95% of targets reached

≥20× coverage. Variants (SNVs and indels) were called with HaplotypeCaller v3.8, and joint genotyping applied via GLnexus (preset GATK).[6] Quad variant analysis was conducted in VarFish.[7]

Subject 6

DNA was extracted from whole blood samples of subject 6 and parents. For all samples WES was performed. DNA enrichment and library preparation were carried out using xGen DNA Lib Prep EZ UNI (Integrated DNA Technologies, Inc., Coralville). Sequencing was conducted on an Illumina NovaSeq X Plus sequencer (Illumina San Diego, CA). Reads were aligned to the human reference genome GRCh38 using megSAP (version megSAP-2022_08-152-gdd675f86; https://github.com/imgag/megSAP). Variant prioritization and visualization were performed with GSvar (version ngs-bits-2024_08-35-g5b45dc95; https://github.com/imgag/ngs-bits), Integrative Genomics Viewer (IGV; version 2.16.0)[8] and Alamut® visual Plus (version 1.8.1; Interactive Biosoftware, Rouen, France).

Subjects 7 and 12

DNA was isolated from EDTA blood samples of subjects 7, 12, and their parents. WES was performed on genomic DNA from subjects 6 and 11 and parents, as previously described.[9] Analysis of the WES data was performed using an in-house pipeline (Exome Variant Annotation Database; https://github.com/mri-ihg/EVAdb). The Burrows-Wheeler Aligner (v.0.7.5a) was used to align sequencing reads to the human genome assembly GRCh37/hg19 (UCSC Genome Browser).[5] Single nucleotide variants, as well as small insertions and deletions, were detected using the Genome Analysis Toolkit.[10] ExomeDepth was used to detect copy number variants.[11] Mitochondrial DNA variants were detected from exonic data as previously reported outlined.[12]

Subjects 8 and 9

DNA was extracted from whole blood samples of subjects 8 and 9, their parents, and two siblings. For all samples WES was performed. DNA enrichment and library preparation were carried out using the

xGen Exome Research Panel (v1 or v2; Integrated DNA Technologies, Inc., Coralville) or TruSeq Exome (Illumina, San Diego, CA). Sequencing was conducted on a NextSeq 500 or on a NovaSeq6000 sequencer. Reads were aligned to the human reference genome (GRCh38) using megSAP (version megSAP-2022_08-152-gdd675f86; https://github.com/imgag/megSAP).

Variant prioritization and visualization were performed with GSvar (version ngs-bits-2024_08-35-g5b45dc95; https://github.com/imgag/ngs-bits), Integrative Genomics Viewer (IGV; v2.16.0)[8] and Alamut® visual Plus (v1.8.1; Interactive Biosoftware, Rouen, France).

Subject 10

Genomic DNA was isolated from amniotic fluid obtained from the mother of subject 10. Double stranded DNA capture baits against approximately 36.5 Mb of the human coding exome (targeting >98% of the coding RefSeq from the human genome build GRCh37/hg19) were used to enrich target regions from fragmented genomic DNA with the Twist Human Core Exome Plus kit. The generated library was sequenced on an Illumina platform to obtain at least 20x coverage depth for >98% of the targeted bases. An in-house bioinformatics pipeline, including read alignment to GRCh37/hg19 genome assembly, variant calling (single nucleotide and small deletion/insertion variants), annotation and comprehensive variant filtering was applied. All variants with minor allele frequency (MAF) of less than 1% in the gnomAD database, and disease-causing variants reported in HGMD®, in ClinVar or in CentoMD® were considered. The investigation for relevant variants was focused on coding exons and flanking +/-20 intronic nucleotides of genes. Variants with low quality and/or unclear zygosity were confirmed by orthogonal methods. Consequently, a specificity of >99.9% for all reported variants was warranted.

To exclude maternal cell contamination, 15 STR autosomal markers plus amelogenin were analyzed using the PowerPlex 16HS multiplex kit (Promega Corporation, Madison, USA).[13]

Subject 11

DNA was isolated from EDTA blood samples of subject 11 and parents and analyzed using WES at the Victorian Clinical Genetics Service (VCGS). As no diagnosis was reached the patient and their parents were recruited into the Undiagnosed Disease Network Australia Research Program, and the Sydney Children's Hospitals Network Undiagnosed Disease Program GeneAdd. Reanalysis of the existing trio whole-genome sequencing (WGS) was performed in a research setting at the Centre for Population Genomics following the DRAGEN GATK best practices pipeline. Reads were aligned to the hg38 reference genome using Dragmap (v1.3.0). Cohort-wide joint calling of single nucleotide variants and small insertion/deletion variants was performed using GATK HaplotypeCaller (v4.1.4.1) with "dragen-mode" enabled. Variants were annotated using VEP v105 and loaded into the web-based variant filtration platform, seqr.[14] Sample sex and relatedness quality checks were performed using Somalier (v0.2.15).[15] Variants were initially curated utilising the transcript predicted to be the most deleterious to the protein and were reported in accordance with HGVS nomenclature. Where no causative variants were identified within the prioritised gene lists, curation was expanded to "off-Mendeliome" genes covered by the original clinical test. Analysis of Short Tandem Repeats was performed using STRipy v2.2 pipeline.[16] Structural variant (SV) calling from short-read WGS data was performed using GATK-SV.[17] The resulting callset was then loaded into CPG instance of seqr for analysis. The GATK mitochondrial variant calling pipeline was used to call homoplasmic and heteroplasmic variants in mtDNA from WGS data using mitochondria mode of GATK MuTect2[18] and a MitoReport (https://github.com/bioinfomethods/mitoreport) was generated for analysis.

Subject 13

WES of isolated DNA from subject 13 and parents was performed at the CeGaT GmbH laboratory (Tübingen, Germany). The coding and flanking intronic regions were enriched using the SureSelect™ target enrichment system (Agilent Technologies, Santa Clara, CA) and were sequenced using the Illumina HiSeq/NovaSeq system. Illumina bcl2fastq2 was used for base calling and demultiplexing. High quality reads were aligned to the human reference genome (hg19) using the Burrows-Wheeler

Aligner (https://bio-bwa.sourceforge.net/). Variants were annotated based on several internal and external databases. Variant classification was performed according to ACMG guidelines for pathogenicity assessment.

Subject 14

Genomic DNA was extracted from peripheral blood samples of subject 14 and parents using standard procedures. Structural genomic variants in the proband were analyzed using a high-resolution single nucleotide polymorphism (SNP) array (Infinium Global Screening Array-24+ v3.0 Kit, GSA-Cyto, Illumina), which includes approximately 700,000 markers with an average inter-marker distance of 4 kb according to the manufacturer's protocol. The resulting karyotype was arr(X,Y)x1,(1-22)x2.
WES was performed using genomic DNA from subject 14, as previously described.[19] Briefly, human protein-coding genes (36.8 Mb in total) were captured from genomic DNA with the Twist Comprehensive Exome Panel and with the Mitochondrial Panel (Twist Bioscience, San Francisco, CA); reagents from the same kits were used to prepare DNA libraries, which were sequenced on a HiSeq platform (Illumina, San Diego, CA) with 150 bp read length in paired-end sequencing mode. The obtained sequencing reads were aligned to the human reference genome (GRCh38) (University of California Santa Clara, Santa Clara, CA) and single nucleotide variants and small indels were called with the Genome Analysis Toolkit (GATK) version 4.0 (https://github.com/broadinstitute/gatk). Sequencing reads were also aligned to the human reference genome (GRCh37) with SeqNext v5.0 (JSI, Kippenheim, Germany). The GRCh37 pipeline includes single nucleotide variants and small indels calling and the detection of single and multiple exon deletions and duplications.

Called variants were filtered for autosomal recessive mode of inheritance (including homozygous variants and two heterozygous variants in the same gene), for X-linked and autosomal dominant inheritance in the proband. Variants were filtered based on their predicted impact on splicing and protein function (missense, nonsense, intronic variants at exon-intron boundaries ranging from -15

to +15, in-frame indels, and frameshift). Only variants with an allele frequency of <0.01 in the gnomAD database (https://gnomad.broadinstitute.org/) were retained. Variants were evaluated *in silico* for pathogenicity by CADD (http://cadd.gs.washington.edu/score); missense variants were evaluated by PolyPhen-2 (http://genetics.bwh.harvard.edu/pph2) and SIFT; and splice site variants were evaluated using SpliceAI lookup (https://spliceailookup.broadinstitute.org/). *WDHD1* variants were validated in subject 13 and segregated in parents by Sanger sequencing using the BigDye terminator v3.1 chemistry on an ABI PRISM 3100 Genetic Analyzer (Applied Biosystems, Fisher Scientific). Sanger traces were evaluated with the Sequencher software.

Subject 15

WGS was performed within the framework of the National Genetic Initiative "100 000+Me". Genomic DNA from subject 15 and parents was sequenced on the DNBSEQ-T7 platform using paired-end sequencing (PE150). Library preparation was carried out with a PCR-free protocol involving enzymatic fragmentation (MGI). Variant nomenclature followed the guidelines of the Human Genome Variation Society (HGVS; http://varnomen.hgvs.org/recommendations/DNA (v20.05). The search for the genetic cause was conducted using the NGS-DATA software (https://ngs-data-ccu.epigenetic.ru/main/; accessed on May 24, 2025). Filtered variants were annotated according to HGVS nomenclature and referenced against the MANE Select transcript for the *WDHD1* gene. Variant classification was performed according to ACMG guidelines for pathogenicity assessment.

Subject 16

DNA was extracted from peripheral blood leukocytes of subject 16 and parents using standard protocols. WES on subject 16's DNA was performed as previously described.[20,21] Libraries were prepared with the Twist Exome 2.0 kit (Twist Bioscience, South San Francisco, CA, USA) following the manufacturer's instructions. Sequencing was performed on an Illumina NovaSeq 6000 (Illumina San Diego, CA) platform in paired-end mode. Reads were aligned to the GRCh37/hg19 human genome assembly

using the Burrows-Wheeler Aligner, and variants were called with Freebayes and GATK. CNVs were analyzed from WES data using clinCNV. Variants were analyzed using the Franklin by Genoox platform (franklin.genoox.com). Variants with a minor allele frequency <0.1% in population databases (gnomAD and ABraOM)[22,23] and located in coding regions or canonical splice sites were retained. Variants were categorized as high-impact (rare, protein-truncating variants: nonsense, canonical splice-site, frameshift) or moderate-impact (rare missense variants predicted deleterious [REVEL ≥0.64] or splice-altering [dbscSNV ≥0.70, SpliceAI ≥0.22]). Final candidate variants were prioritized based on zygosity, inheritance, gene function, protein expression, relevant phenotypes in humans or animal models, and literature or database evidence. *WDHD1* variants were validated and segregated in parents by Sanger sequencing using the BigDye terminator on an ABI PRISM 3100 Genetic Analyzer (Applied Biosystems, Fisher Scientific).

<u>Subject 17</u>

Reanalysis of the existing trio WGS was performed in a research setting at the Centre for Population Genomics following the DRAGEN GATK best practices pipeline. Reads were aligned to the hg38 reference genome using Dragmap (v1.3.0). Cohort-wide joint calling of single nucleotide variants and small insertion/deletion variants was performed using GATK HaplotypeCaller (v4.1.4.1) with “dragen-mode” enabled. Variants were annotated using VEP v105 and loaded into the web-based variant filtration platform, seqr.[14] Sample sex and relatedness quality checks were performed using Somalier (v0.2.15).[15] Variants were initially curated utilising the transcript predicted to be the most deleterious to the protein and were reported in accordance with HGVS nomenclature. Where no causative variants were identified within the prioritised gene lists, curation was expanded to “off-Mendeliome” genes covered by the original clinical test. Analysis of Short Tandem Repeats was performed using STRipy v2.2 pipeline.[16] Structural variant (SV) calling from short-read WGS data was performed using GATK-SV.[17] The resulting callset was then loaded into CPG instance of seqr for analysis. The GATK mitochondrial variant calling pipeline was used to call homoplasmic and heteroplasmic variants in

mtDNA from WGS data using mitochondria mode of GATK MuTect2[18] and a MitoReport (https://github.com/bioinfomethods/mitoreport) was generated for analysis. Variant(s) have been classified according to the American College of Medical Genetics and Genomics and the Association for Molecular Pathology joint consensus recommendations for the interpretation of sequence variants[4] with ClinGen recommendations.

Variant validation by Sanger sequencing

The biallelic *WDHD1* variants (NM_007086.4) or the reference sequence were validated and/or segregated in fibroblast-derived DNA from the subjects and in fibroblasts from healthy donors using Sanger sequencing. Primer sequences are described in **Supplementary Table 2**. PCR amplicons were sequenced using the ABI BigDye Terminator Sequencing kit (Applied Biosystems) and an automated capillary sequencer (ABI 3500, Applied Biosystems). Sequence electropherograms were analyzed using Chromas v2.6.6 (Technelysium Pty Ltd).

Supplemental references

1. Fowler, A., Mahamdallie, S., Ruark, E., Seal, S., Ramsay, E., Clarke, M., Uddin, I., Wylie, H., Strydom, A., Lunter, G., and Rahman, N. (2016). Accurate clinical detection of exon copy number variants in a targeted NGS panel using DECoN. Wellcome Open Res *1*, 20. 10.12688/wellcomeopenres.10069.1.
2. Fromer, M., Moran, J.L., Chambert, K., Banks, E., Bergen, S.E., Ruderfer, D.M., Handsaker, R.E., McCarroll, S.A., O'Donovan, M.C., Owen, M.J., et al. (2012). Discovery and statistical genotyping of copy-number variation from whole-exome sequencing depth. Am J Hum Genet *91*, 597-607. 10.1016/j.ajhg.2012.08.005.
3. Krumm, N., Sudmant, P.H., Ko, A., O'Roak, B.J., Malig, M., Coe, B.P., Project, N.E.S., Quinlan, A.R., Nickerson, D.A., and Eichler, E.E. (2012). Copy number variation detection and genotyping from exome sequence data. Genome Res *22*, 1525-1532. 10.1101/gr.138115.112.
4. Richards, S., Aziz, N., Bale, S., Bick, D., Das, S., Gastier-Foster, J., Grody, W.W., Hegde, M., Lyon, E., Spector, E., et al. (2015). Standards and guidelines for the interpretation of sequence variants: a joint consensus recommendation of the American College of Medical Genetics and Genomics and the Association for Molecular Pathology. Genet Med *17*, 405-424. 10.1038/gim.2015.30.
5. Li, H., and Durbin, R. (2009). Fast and accurate short read alignment with Burrows-Wheeler transform. Bioinformatics *25*, 1754-1760. 10.1093/bioinformatics/btp324.
6. Yun, T., Li, H., Chang, P.C., Lin, M.F., Carroll, A., and McLean, C.Y. (2021). Accurate, scalable cohort variant calls using DeepVariant and GLnexus. Bioinformatics *36*, 5582-5589. 10.1093/bioinformatics/btaa1081.
7. Holtgrewe, M., Stolpe, O., Nieminen, M., Mundlos, S., Knaus, A., Kornak, U., Seelow, D., Segebrecht, L., Spielmann, M., Fischer-Zirnsak, B., et al. (2020). VarFish: comprehensive DNA variant analysis for diagnostics and research. Nucleic Acids Res *48*, W162-W169. 10.1093/nar/gkaa241.
8. Robinson, J.T., Thorvaldsdottir, H., Winckler, W., Guttman, M., Lander, E.S., Getz, G., and Mesirov, J.P. (2011). Integrative genomics viewer. Nat Biotechnol *29*, 24-26. 10.1038/nbt.1754.
9. Zech, M., Jech, R., Boesch, S., Skorvanek, M., Weber, S., Wagner, M., Zhao, C., Jochim, A., Necpal, J., Dincer, Y., et al. (2020). Monogenic variants in dystonia: an exome-wide sequencing study. Lancet Neurol *19*, 908-918. 10.1016/S1474-4422(20)30312-4.
10. Van der Auwera, G.A., Carneiro, M.O., Hartl, C., Poplin, R., Del Angel, G., Levy-Moonshine, A., Jordan, T., Shakir, K., Roazen, D., Thibault, J., et al. (2013). From FastQ data to high confidence variant calls: the Genome Analysis Toolkit best practices pipeline. Curr Protoc Bioinformatics *43*, 11 10 11-11 10 33. 10.1002/0471250953.bi1110s43.
11. Plagnol, V., Curtis, J., Epstein, M., Mok, K.Y., Stebbings, E., Grigoriadou, S., Wood, N.W., Hambleton, S., Burns, S.O., Thrasher, A.J., et al. (2012). A robust model for read count data in exome sequencing experiments and implications for copy number variant calling. Bioinformatics *28*, 2747-2754. 10.1093/bioinformatics/bts526.
12. Wagner, M., Berutti, R., Lorenz-Depiereux, B., Graf, E., Eckstein, G., Mayr, J.A., Meitinger, T., Ahting, U., Prokisch, H., Strom, T.M., and Wortmann, S.B. (2019). Mitochondrial DNA mutation analysis from exome sequencing-A more holistic approach in diagnostics of suspected mitochondrial disease. J Inherit Metab Dis *42*, 909-917. 10.1002/jimd.12109.
13. Trujillano, D., Bertoli-Avella, A.M., Kumar Kandaswamy, K., Weiss, M.E., Koster, J., Marais, A., Paknia, O., Schroder, R., Garcia-Aznar, J.M., Werber, M., et al. (2017). Clinical exome sequencing: results from 2819 samples reflecting 1000 families. Eur J Hum Genet *25*, 176-182. 10.1038/ejhg.2016.146.
14. Pais, L.S., Snow, H., Weisburd, B., Zhang, S., Baxter, S.M., DiTroia, S., O'Heir, E., England, E., Chao, K.R., Lemire, G., et al. (2022). seqr: A web-based analysis and collaboration tool for rare disease genomics. Hum Mutat *43*, 698-707. 10.1002/humu.24366.

15. Pedersen, B.S., Bhetariya, P.J., Brown, J., Kravitz, S.N., Marth, G., Jensen, R.L., Bronner, M.P., Underhill, H.R., and Quinlan, A.R. (2020). Somalier: rapid relatedness estimation for cancer and germline studies using efficient genome sketches. Genome Med *12*, 62. 10.1186/s13073-020-00761-2.
16. Halman, A., Dolzhenko, E., and Oshlack, A. (2022). STRipy: A graphical application for enhanced genotyping of pathogenic short tandem repeats in sequencing data. Hum Mutat *43*, 859-868. 10.1002/humu.24382.
17. Collins, R.L., Brand, H., Karczewski, K.J., Zhao, X., Alfoldi, J., Francioli, L.C., Khera, A.V., Lowther, C., Gauthier, L.D., Wang, H., et al. (2020). A structural variation reference for medical and population genetics. Nature *581*, 444-451. 10.1038/s41586-020-2287-8.
18. Laricchia, K.M., Lake, N.J., Watts, N.A., Shand, M., Haessly, A., Gauthier, L., Benjamin, D., Banks, E., Soto, J., Garimella, K., et al. (2022). Mitochondrial DNA variation across 56,434 individuals in gnomAD. Genome Res *32*, 569-582. 10.1101/gr.276013.121.
19. Hackl, L., Haberlandt, E., Muller, T., Piribauer, S., Garczarczyk-Asim, D., Zoggeler, T., Karall, D., Zschocke, J., and Janecke, A.R. (2025). Homozygous DHCR7 p.Val330Met Variant Associated with Mild Non-Syndromic Intellectual Disability and Elevated Serum 7-Dehydrocholesterol Levels in Two Siblings. Genes (Basel) *16*. 10.3390/genes16070838.
20. Kim, G.J., Vasco de Albuquerque Albuquerque, E., Rezende, R.C., De Polli Cellin, L., Santillan Vasconez, A.M., Krepischi, A.C.V., Santana, L., Lerario, A.M., de Souza, V., Scalco, R., and Jorge, A.A.L. (2025). Exome sequencing of patients with syndromic tall stature reveals four novel candidate genes. Endocr Connect *14*. 10.1530/EC-25-0137.
21. Rezende, R.C., Menezes de Andrade, N.L., Branco Dantas, N.C., de Polli Cellin, L., Victorino Krepischi, A.C., Lerario, A.M., and de Lima Jorge, A.A. (2024). Exome Sequencing Identifies Multiple Genetic Diagnoses in Children with Syndromic Growth Disorders. J Pediatr *265*, 113841. 10.1016/j.jpeds.2023.113841.
22. Chen, S., Francioli, L.C., Goodrich, J.K., Collins, R.L., Kanai, M., Wang, Q., Alfoldi, J., Watts, N.A., Vittal, C., Gauthier, L.D., et al. (2024). A genomic mutational constraint map using variation in 76,156 human genomes. Nature *625*, 92-100. 10.1038/s41586-023-06045-0.
23. Naslavsky, M.S., Scliar, M.O., Yamamoto, G.L., Wang, J.Y.T., Zverinova, S., Karp, T., Nunes, K., Ceroni, J.R.M., de Carvalho, D.L., da Silva Simoes, C.E., et al. (2022). Whole-genome sequencing of 1,171 elderly admixed individuals from Sao Paulo, Brazil. Nat Commun *13*, 1004. 10.1038/s41467-022-28648-3.
