# Supplementary material for: Bi-allelic WDHD1 variants cause microcephalic primordial dwarfism
Source: Am J Hum Genet. 2026 Apr 9;113(5):1067–89. doi: 10.1016/j.ajhg.2026.03.010 (PMC13277691; doi:10.1016/j.ajhg.2026.03.010)
Supplement: Document S2. Article plus supplemental information [file mmc2.pdf]

# Bi-allelic *WDHD1* variants cause microcephalic primordial dwarfism

## Graphical abstract

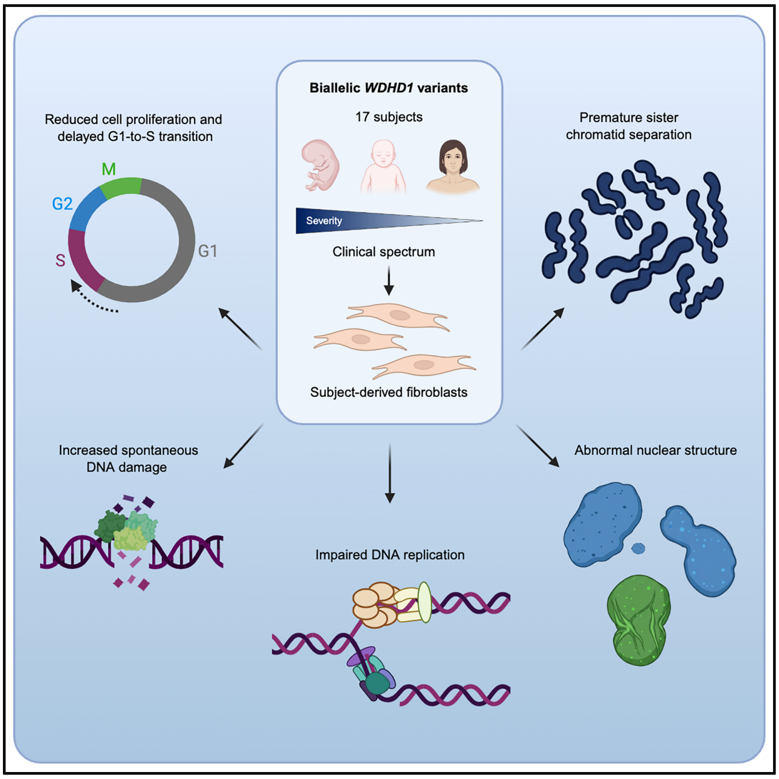



## Authors

Debora Tibbe, Marie Ronja Vogt, Tess Holling, ..., Holger Prokisch, Kerstin Borgmann, Kerstin Kutsche

## Correspondence

kkutsche@uke.de



**We describe 17 subjects with bi-allelic *WDHD1* variants and a clinical spectrum ranging from early fetal lethality to microcephalic primordial dwarfism without developmental delay. Subject-derived fibroblasts showed impaired cell proliferation, delayed cell cycle progression, reduced DNA replication speed, increased DNA damage, premature sister chromatid separation, and abnormal nuclear morphology.**



Tibbe et al., 2026, The American Journal of Human Genetics *113*, 1067–1089
May 7, 2026 © 2026 The Authors. Published by Elsevier Inc. on behalf of American Society of Human Genetics.
https://doi.org/10.1016/j.ajhg.2026.03.010

# Bi-allelic *WDHD1* variants cause microcephalic primordial dwarfism

Debora Tibbe,[1] Marie Ronja Vogt,[2] Tess Holling,[1] Lea Dewi Schlieben,[3] Fanny Kortüm,[1] Moneef Shoukier,[4] Christoph Bagowski,[4] Felix Distelmaier,[5] Luisa Averdunk,[5] Alexej Knaus,[6] Peter Krawitz,[6] Alma Kuechler,[7] Elke Lainka,[8] Amelie Stalke,[9] Sandra von Hardenberg,[9] Bernd Auber,[9] Eva-Doreen Pfister,[10] Bruno Reversade,[11] Anthony Sabbagh,[12] Aida M. Bertoli-Avella,[13] Salem Alawbathani,[13] Elizabeth E. Palmer,[14] Manisha Chauhan,[15] Rocio Rius,[16,17] Yoonji Kim,[14] Australian Undiagnosed Diseases Network (UDN-Aus),[18] Dzhoy Papingi,[1] Deborah Bartholdi,[19] Dominique Braun,[19] Oliver Maier,[20] April Dinwiddie,[21] Elisabeth Steichen-Gersdorf,[22] Andreas R. Janecke,[22,23] Anatoly Tiulpakov,[24] Nikolay Zernov,[25] Maria Izabel Arismendi,[26] Alexander A.L. Jorge,[26] Himanshu Goel,[27,28] Lauren Dreyer,[29] Lily Loughman,[29] Holger Prokisch,[3,30,31] Kerstin Borgmann,[2] and Kerstin Kutsche[1,32,*]

## Summary

DNA replication is carried out by the replisome and is essential for maintaining genome integrity and cell proliferation. Pathogenic variants in genes encoding various replisome components cause microcephalic primordial dwarfism (MPD), characterized by growth retardation, microcephaly, and developmental abnormalities. Here, we report bi-allelic hypomorphic variants in *WDHD1* as a cause of MPD with a broad spectrum of additional abnormalities, including acute liver failure, in 17 subjects from 14 families. *WDHD1* encodes a replisome scaffolding protein (also known as AND-1 and Ctf4), which is essential for replisome assembly, replication fork stability, and sister chromatid cohesion. We found aberrant splicing of *WDHD1* pre-mRNAs for all intronic variants tested and markedly reduced WDHD1 protein levels in subject-derived fibroblasts. Fibroblasts with bi-allelic *WDHD1* variants showed globally reduced replication fork speed and impaired replication control, accompanied by spontaneous DNA damage and a G1-to-S transition defect. Using various cell biology approaches, we show that subject fibroblasts displayed reduced proliferation, abnormal nuclear morphology, including micronuclei, multilobed, and enlarged nuclei, as well as an increased number of metaphases with premature sister chromatid separation. Together, our findings establish WDHD1 as a protein required for normal organismal growth and development in humans and underscore its multiple functions in maintaining genome integrity.

## Introduction

Duplication of genomic material is essential for cellular proliferation and is initiated at origins of replication during the late G1 phase of the cell cycle, followed by efficient DNA synthesis in the subsequent S phase. Progression through both G1 and S phases is tightly regulated by key cell cycle proteins.[1] DNA replication proceeds bidirectionally from the licensed origins and is carried out by the replisome, a multi-protein complex at the replication fork.[2]

[1]Institute of Human Genetics, University Medical Center Hamburg-Eppendorf, 20246 Hamburg, Germany; [2]Department of Radiotherapy & Radiation Oncology, Hubertus Wald Tumor Center - University Cancer Center Hamburg, University Medical Center Hamburg-Eppendorf, 20246 Hamburg, Germany; [3]Institute of Human Genetics, School of Medicine and Health, Technical University Munich, 81675 Munich, Germany; [4]Eurofins Humangenetik und Pränatal-Medizin MVZ GmbH, 80639 Munich, Germany; [5]Department of General Pediatrics, Neonatology and Pediatric Cardiology, University Children's Hospital, Heinrich-Heine-University Düsseldorf, 40225 Düsseldorf, Germany; [6]Institute for Genomic Statistics and Bioinformatics, University Hospital Bonn, 53113 Bonn, Germany; [7]Institute of Human Genetics, University Hospital Essen, University Duisburg-Essen, 45147 Essen, Germany; [8]University Children's Hospital Essen, Pediatric Gastroenterology, Rheumatology, Transplant Medicine, 45147 Essen, Germany; [9]Department of Human Genetics, Hannover Medical School, 30625 Hannover, Germany; [10]Department for Pediatric Kidney, Liver and Metabolic Diseases, Hannover Medical School, 30625 Hannover, Germany; [11]Laboratory of Human Genetics & Therapeutics, King Abdullah University of Science and Technology, Thuwal 23955, Saudi Arabia; [12]Universite Saint Joseph de Beyrouth, Faculte de Medecine Damascus, Beirut 1107-2180, Lebanon; [13]CENTOGENE GmbH, 18055 Rostock, Germany; [14]Discipline of Paediatrics and Child Health, School of Clinical Medicine, Faculty of Medicine and Health, University of New South Wales Sydney, Sydney, NSW 2031, Australia; [15]Centre for Clinical Genetics, Sydney Children's Hospitals Network, Randwick, Sydney, NSW 2031, Australia; [16]Centre for Population Genomics, Garvan Institute of Medical Research and UNSW Sydney, Sydney, NSW 2031, Australia; [17]Centre for Population Genomics Murdoch Children's Research Institute, Melbourne, VIC 3051, Australia; [18]Australian Undiagnosed Diseases Network (UDN-Aus), Murdoch Children's Research Institute, Melbourne, VIC 3052, Australia; [19]INSELSPITAL, University Hospital Bern Department of Human Genetics, 3010 Bern, Switzerland; [20]Department of Child Neurology, Developmental Medicine and Rehabilitation, Children's Hospital of Eastern Switzerland, 9006 St. Gallen, Switzerland; [21]CeGaT, 72076 Tübingen, Germany; [22]Department of Pediatrics I, Medical University of Innsbruck, 6020 Innsbruck, Austria; [23]Institute of Human Genetics, Medical University of Innsbruck, 6020 Innsbruck, Austria; [24]Department of Endocrine Genetics, Research Centre for Medical Genetics, Moscow 115522, Russian Federation; [25]Biotech Campus LLC, Moscow 117997, Russian Federation; [26]Genetic Endocrinology Unit (LIM25), Endocrinology Division, Faculdade de Medicinada Universidade de São Paulo (HC-FMUSP), São Paulo 01246-903, Brazil; [27]Hunter Genetics, Waratah, NSW 2298, Australia; [28]University of Newcastle, Callaghan, NSW 2308, Australia; [29]Genetic Health Western Australia, Perth, WA 6008, Australia; [30]Institute of Neurogenomics, Computational Health, Helmholtz Zentrum München, 85764 Munich, Germany; [31]German Center for Child and Adolescent Health (DZKJ), Partner Site Munich, 80337 Munich, Germany; [32]German Center for Child and Adolescent Health (DZKJ), Partner Site Hamburg, 20251 Hamburg, Germany
*Correspondence: kkutsche@uke.de
https://doi.org/10.1016/j.ajhg.2026.03.010.
© 2026 The Authors. Published by Elsevier Inc. on behalf of American Society of Human Genetics.
This is an open access article under the CC BY license (http://creativecommons.org/licenses/by/4.0/).

The assembly of the pre-replisome complex begins with the formation of the Cdc45-MCM-GINS (CMG) complex, which has helicase activity.[3,4] On the leading strand, DNA is catalyzed by DNA polymerase ε, while on the lagging strand, synthesis is initiated by the DNA polymerase α-primase complex and completed by DNA polymerase δ.[5] Additional accessory factors complete the replisome and ensure high-fidelity DNA replication.[2] One such factor is WDHD1, also known as AND-1 in mammals and Ctf4 in *Saccharomyces cerevisiae*, which plays a critical role during DNA replication.[6] In human cells, the homotrimeric scaffolding protein WDHD1 is required for the formation of the CMG complex and for the binding to and stabilization of the DNA polymerase α-primase complex.[7–11] Recent data show that human WDHD1 enhances leading-strand replication, while its interaction with DNA polymerase α is not required for lagging-strand synthesis.[12]

Mammalian WDHD1 is required for cell cycle progression, cell growth, and repair of DNA double-strand breaks by homologous recombination.[13–16] Furthermore, WDHD1 is essential for protecting replication forks from nucleolytic processing, thereby preventing the formation of long single-stranded DNA (ssDNA) regions.[16] Human WDHD1 interacts with several proteins involved in sister chromatid cohesion,[13] and *Xenopus* wdhd1 is required for chromosome cohesion,[17,18] a crucial process for the distribution of genetic information between daughter cells.[19] In *S. cerevisiae*, Ctf4 coordinates both the replisome and the sister chromatid cohesion complex to ensure efficient progression of the replication fork.[20]

DNA replication stress triggers the activation of the intra-S checkpoint. DNA lesions can cause replication fork stalling followed by the generation of DNA double-strand breaks, which are a major source of genome rearrangements.[21,22] In *S. cerevisiae*, Ctf4 suppresses the formation of DNA double-strand breaks at the arrested replication fork and prevents chromosome rearrangements.[23] In human cells, WDHD1 is phosphorylated upon replication stress, accumulates at DNA damage sites, recruits the intra-S-phase checkpoint kinase 1 (Chk1) to stalled forks, and is important for fork recovery.[24] Collectively, these and other data underscore an important role of WDHD1 in the maintenance of genomic integrity, most likely by interacting with multiple proteins and enzymes that counteract replication stress.[16,23–26]

Precise and timely DNA replication is essential for cell division and differentiation and is therefore fundamental to human development and the maintenance of tissue homeostasis.[27] Hypomorphic variants in genes encoding replisome components have been associated with several Mendelian disorders, highlighting the critical role of the replication machinery in genomic stability and human health.[28] One such group of DNA replication-associated diseases is microcephalic primordial dwarfism (MPD), which is characterized by both pre- and postnatal growth retardation and microcephaly, with or without additional developmental abnormalities.[29,30] MPD is genetically heterogeneous. Meier-Gorlin syndrome (MIM: PS224690) belongs to MPD and is characterized by the triad of short stature, microtia, and patella hypo- or aplasia. Pathogenic variants in at least 13 genes encoding components of the DNA replication machinery have been associated with this disorder, including twelve genes with essential roles in early DNA replication and nine involved in pre-replication complex formation.[31] Bi-allelic variants in *DONSON* (MIM: 611428), *TRAIP* (MIM: 605958), and *DNA2* (MIM: 601810), which code for proteins involved in DNA replication and/or DNA repair pathways, cause MPD or growth restriction with additional abnormalities.[32–36] In addition, pathogenic variants in other genes encoding components of distinct DNA polymerases, such as *POLA1* (MIM: 312040), *POLD1* (MIM: 174761), *POLD2* (MIM: 600815), *POLE* (MIM: 174762), and *PRIM1* (MIM: 176635), have been associated with a range of Mendelian disorders.[28,37,38]

Here, we report the identification and functional characterization of bi-allelic variants in *WDHD1* (MIM: 608126) in subjects with MPD, along with a range of additional developmental abnormalities in most affected individuals. Of the twelve different *WDHD1* variants, seven are non-coding; five of these were experimentally shown to affect *WDHD1* pre-mRNA splicing. We demonstrate that markedly reduced WDHD1 protein levels in subject-derived fibroblasts permit cell survival but impair several critical cellular functions, including the cell cycle, DNA replication, sister chromatid pairing, and nuclear envelope integrity.

## Subjects and methods

### Ethical approval

All work was performed in accordance with the ethical standards of the relevant institutional and national committees for such matters and the WMS Declaration of Helsinki on ethical principles for medical research. Genetic studies (see the supplemental methods) were approved by local institutional review boards (IRBs), including the IRB of the Heinrich-Heine University Düsseldorf, Düsseldorf, Germany (2021-1340/FD-LA), for subjects 4 and 5 (S4 and S5); the ethics committee of Hannover Medical School, Hannover, Germany (10235_BO_S_2022, 7656-2017, and 8657_BO_K_2019), for S6, S8, and S9; the ethics committee of the Klinikum Rechts der Isar, Technical University of Munich, Munich, Germany (TUM 5360/13), for S7; the IRB of the Agency for Science, Technology and Research (A*STAR), Singapore (2019-087), and the Institutional Biosafety and Bioethics Committee of the King Abdullah University of Science and Technology, Thuwal, Saudi Arabia (protocol 23IBEC090), for S10; the Sydney Children's Hospitals Networks (SCHN) Human Research Ethics Committee (HREC) (reference number 2019/ETH12990) for the GeneAdd study and the Royal Children's Hospital for the UDNAus Study (reference number UDN-Aus: RCH79712), for S11; the ethics committee of the Hamburg Medical Chamber, Hamburg, Germany (PV7038-4438-BO-ff), for S12; the Cantonal Ethics Board Bern (KEK) Bern, Switzerland (2021-01396), for

S13; the IRB of the Medical University of Innsbruck, Innsbruck, Austria (no. UN4501), for S14; the ethics committee of Research Centre for Medical Genetics, Moscow, Russian Federation (2024-04081), for S15; the ethics committee of Hospital das Clinicas da Faculdade de Mediciana da Universidade de Sao Paulo (37868114.3.0000.0068), Sao Paulo, Brasilia, for S16; and the King Edward Memorial Hospital Research Governance and Human Research Ethics Committee (PRN: RGS0000005438), Perth, Australia, for S17. Genetic studies were performed clinically in S1, S2, and S3, and their legal guardians provided informed written consent for genetic testing. The parents of all subjects provided written informed consent for participation in the study, clinical data and specimen collection, genetic analysis, and publication of relevant findings. Written informed consent for the publication of photographs was obtained for S5, S11–S14, S16, and S17.

## Cell culture

Primary fibroblasts were obtained from skin biopsies of S5, S6, and S12–S14, as well as three healthy 4-year-old girls (control individuals 1–3) and one additional 4-year-old girl with unknown health status (control individuals 4). Cells were cultured in FibroPlus 333 Complete Medium for Fibroblasts, with L-Glutamine (Capricorn Scientific), supplemented with penicillin-streptomycin (100 U/mL and 100 μg/mL, Thermo Fisher Scientific), and incubated at 37°C in a humidified atmosphere with 5% $CO_2$. Cells were confirmed to be mycoplasma-free by PCR testing.

## RNA isolation, cDNA synthesis, and transcript analysis

Total RNA was extracted from leukocytes (PAXgene Blood RNA Tubes; PreAnalytiX, #762165) and from fibroblasts using the PAXgene Blood RNA Kit (PreAnalytiX, #762174) and the Monarch Total RNA Miniprep Kit (New England Biolabs, #T2010S), respectively, according to the manufacturers' instructions. RNA concentration and purity were assessed using the Epoch Microplate Spectrophotometer (BioTek Instruments). For each sample, 1 μg of RNA was reverse transcribed into complementary DNA (cDNA) using the LunaScript RT SuperMix Kit (New England Biolabs, #E3010L). Reverse-transcription PCR (RT-PCR) was performed using OneTaq Quick-Load 2× Master Mix (New England Biolabs, #M0486L) with primers described in Table S2. RT-PCR products were analyzed by agarose gel electrophoresis, followed by direct Sanger sequencing. Alternatively, RT-PCR amplicons were cloned into the pCR2.1-TOPO vector using the TOPO TA cloning kit (Thermo Fisher Scientific, #K450002) or the pcDNA3.1 vector using the In-Fusion cloning system (Takara, #639298 and #639649) according to the manufacturers' instructions. Single *E. coli* clones were either analyzed by colony PCR with vector-specific primers (M13 reverse and T7 forward for pCR2.1 TOPO TA; Table S2) followed by Sanger sequencing, or plasmid DNA was isolated and the cloned inserts Sanger sequenced (for the In-Fusion cloning).

## Real-time quantitative PCR

Real-time quantitative PCR (real-time qPCR) was conducted using the Luna Universal qPCR Master Mix (New England Biolabs, #M3003L) in 10 μL reactions containing 500 nM of each primer and 1 μL of cDNA. Sequences of the two applied real-time qPCR primer pairs are described in Table S2. All reactions were performed in technical triplicates on the QuantStudio 3 Real-Time PCR System (Thermo Fisher Scientific) under the following cycling conditions: initial denaturation at 95°C for 5 min, followed by 40 cycles of 95°C for 30 s, 58°C for 30 s, and 72°C for 45 s. Melting curve analysis (from 60°C to 95°C) was used to confirm the specificity of the amplification. Target transcript levels were normalized to *GAPDH* as a housekeeping gene, and relative transcript levels were calculated using the ΔΔCT method. Data analysis was performed using the QuantStudio Design & Analysis Software v.1.4.3 (Thermo Fisher Scientific).

## Antibodies and conjugates

The primary antibodies and dilutions used were rabbit polyclonal anti-WDHD1 (immunoblot [IB]: 1:250, Novus Biologicals, #NBP1-89091), mouse monoclonal anti-phosphorylated histone H2AX (γH2AX; Ser139) (immunofluorescence [IF]: 1:500, clone JBW301 ZooMAb, Sigma Aldrich, #05-636), rabbit polyclonal anti-lamin B1 (IF: 1:500, Abcam, #ab16048), anti-RPA 32 kDa subunit (IB: 1:1,000, Santa Cruz, #sc53496), and anti-pRPA32 (Ser4 and Ser8) (IB: 1:1,000, Thermo Fisher Scientific, #A300-245A).

The secondary antibodies and dilutions used were goat anti-rabbit IgG StarBright Blue 700 (IB: 1:5,000, Bio-Rad, #12004161) and goat anti-mouse IgG secondary antibody Alexa Fluor 488 conjugated (IF: 1:500, Invitrogen, #A11029). The conjugated antibody used was anti-GAPDH hFAB Rhodamine (IB: 1:10,000, Bio-Rad, #12004167).

## IB analysis

Fibroblasts were seeded in six-well plates at a density of $1.5 \times 10^5$ cells per well. Upon reaching ~80% confluence, cells were washed with cold PBS and harvested in ice-cold radioimmunoprecipitation assay (RIPA) buffer (50 mM Tris-HCl [pH 8.0], 150 mM NaCl, 1% NP-40, 0.5% sodium deoxycholate, and 0.1% SDS) supplemented with protease and phosphatase inhibitors (Roche, Mini Protease Inhibitor Cocktail, #11836170001, and PhosSTOP #PHOSS-RO). For the detection of RPA2 and phospho-RPA2, fibroblasts were treated with either 2 mM hydroxyurea (HU; Sigma-Aldrich) or an equivalent volume of DMSO in complete culture medium for 4 h prior to cell lysis. Lysates were incubated on ice for 10 min and clarified by centrifugation at $13{,}000 \times g$ for 10 min at 4°C. The lysates were mixed with 4× sample buffer (33% glycerol, 80 mM Tris-HCl [pH 6.8], 0.3 M DTT, 6.7% SDS, and 0.1% bromophenol blue) and denatured by heating at 95°C for 5 min. Protein extracts were separated by SDS-PAGE and transferred to polyvinylidene fluoride membranes. Membranes were blocked with 5% non-fat dried milk or 5% bovine serum albumin (BSA) in Tris-buffered saline-Tween (TBS-T; 20 mM Tris-HCl [pH 7.5], 150 mM NaCl, and 0.1% Tween 20) followed by incubation with the indicated primary antibody at 4°C overnight and with secondary antibodies at room temperature for 1 h. Signals were digitally imaged with a ChemiDoc MP imaging system (Bio-Rad). Band intensities were quantified with Image Lab software (v.6.0; Bio-Rad), and data were normalized to GAPDH as a loading control.

## Proliferation assay

Fibroblasts were seeded at a density of $1 \times 10^5$ cells in five wells of a 6-well plate each and maintained under standard culture conditions, without medium changes or passaging. Cell numbers were determined every 2 days over a period of 10 days. At each time

point, cells were harvested by trypsinization and counted using a Vi-CELL XR cell counter (Beckman Coulter).

## Cell cycle analysis

$1 \times 10^5$ fibroblasts were seeded onto 6-well plates. The following day, fibroblasts were rinsed with PBS, harvested by trypsinization, and transferred into flow cytometry tubes. Cells were collected by centrifugation at $600 \times g$ for 5 min, washed once with PBS, and fixed with 300 μL 70% ethanol for 30 min at 4°C. After centrifugation, cells were incubated with 50 μL of 100 μg/mL RNase A/T1 mix (Thermo Fisher Scientific) for 20 min at 37°C, followed by staining with 100 μL of 50 μg/mL propidium iodide (PI) (Sigma-Aldrich) for 20 min at room temperature in the dark. Fluorescence signals were acquired using a FACS NovoCyte Quanteon (Agilent) equipped with a 598 mirror and 615/20 nm band-pass filter, using a 488 nm excitation laser. Data were collected and analyzed using NovoExpress software v.1.6.2 (Agilent).

## DNA fiber assay

Exponentially growing fibroblasts were sequentially labeled with 25 μM CldU (Sigma-Aldrich) and 250 μM IdU (Sigma-Aldrich) for 30 min each. 2 mM HU was added to the cells between the two labeling steps for 120 min. Cells were then harvested, and DNA fibers were prepared, spread, fixed, and stained as previously described.[39,40] Fiber analysis was performed using an Axioplan 2 fluorescence microscope (Carl Zeiss Microscopy). Track lengths were measured using ImageJ 1.54g software. For each experiment, a minimum of 50 replication tracks or structures were analyzed from 3–4 independent experiments.

## IF analysis and quantification of γH2AX foci and nuclear morphology

$5 \times 10^4$ fibroblasts were cultivated on glass coverslips in 12-well plates, fixed with ice-cold paraformaldehyde (PFA; 4% in PBS), and washed three times with PBS. After treatment with permeabilization/blocking solution (2% BSA, 3% goat serum, and 0.5% NP-40 in PBS), cells were incubated in antibody solution (3% goat serum and 0.1% NP-40 in PBS) containing the primary rabbit anti-γH2AX or primary rabbit anti-lamin B1 antibody. Cells were washed with PBS and incubated with goat anti-rabbit Alexa Fluor 488-coupled secondary antibody. After extensive washing with PBS, cells were embedded in mounting solution with DAPI (Invitrogen) and imaged with a Zeiss ApoTome microscope with a 63× oil-immersion objective.

For quantification of the γH2AX foci and nuclear morphology, between 126 and 202 cells per cell line were analyzed (Table S3). Fluorescence images were acquired under identical imaging conditions across slides and three independent experiments. Image analysis was performed using Fiji (ImageJ, v.1-54f, http://imagej.org). The contrast of green channel images was maximized, and the remaining green puncta were counted as γH2AX foci using the multi-point tool. Area quantification of the nucleus was performed by manually outlining DAPI-stained nuclei using the polygon selections tool. Nuclear morphology was categorized into three groups based on DAPI-stained nuclear shape, such as multilobed, micronuclei, and general abnormal morphology. Multilobed nuclei were defined as nuclei with deep indentations or constrictions, giving the appearance of two or more connected round compartments. Micronuclei were defined as distinct, small, round DAPI-positive structures located directly adjacent to or in close proximity to, but separated from, the main nucleus. Nuclei of the general abnormal morphology category included all nuclei lacking lobes or micronuclei but exhibiting irregular shape (e.g., wavy contours) and/or protrusions. Nuclei without any of these alterations were classified as morphologically normal. Nuclei that were overlapping, out of focus, or undergoing mitosis were excluded from the analysis.

## Preparation of mitotic spreads and analysis of premature chromatid separation

Mitotically active fibroblasts from passages 8–15 were maintained in FibroPlus 333 Complete Medium for Fibroblasts, with L-Glutamine (Capricorn Scientific), supplemented with penicillin-streptomycin (100 U/mL and 100 μg/mL, Thermo Fisher Scientific). Cultures with ~60% confluence were used for mitotic spread preparation. To arrest cells in metaphase, cells were treated with 0.5 μg/mL Colcemid for 6 h. Cells were harvested by trypsinization, collected in 15 mL centrifuge tubes, and pelleted at 1,000 rpm for 10 min. Pellets were resuspended in 7 mL prewarmed 0.075 M KCl solution and incubated at 37°C for 15 min to induce hypotonic swelling. Cells were fixed by dropwise addition of ice-cold methanol:acetic acid (3:1) while gently mixing the suspension, followed by three successive washes with fixative, each centrifuged at 1,000 rpm for 10 min. Final cell suspensions were prepared with 200 μL fixative and stored at −20°C until metaphase preparation. Frozen cell suspensions were thawed on ice and mixed gently. Metaphase spreads were prepared in a humidity-controlled environment with 50% humidity using a laboratory humidifier at ~21°C. Glass slides were precleaned in glass staining jars containing a Mucasol/deionized water solution and incubated at 4°C overnight. Slides were briefly dried on filter paper to remove excess liquid without allowing the surface to dry completely. Approximately 100 μL of the cell suspension was pipetted using a cut-tip pipette and dropped from a height of ~50 cm onto the slide in a raster pattern to facilitate optimal chromosome spreading. Excess liquid was removed gently with filter paper, and slides were placed in the preheated incubator at 94°C for 2 min to aid spreading. Slides were then inspected under a phase-contrast microscope to identify metaphases suitable for analysis. Slides were stained with a 10% Giemsa solution, prepared by diluting Giemsa stock solution in phosphate buffer (Weise buffer, Titrisol [pH 7.2], Merck). Then, slides were mounted and dried overnight. Images of mitoses were acquired with the Leica DM2500 upright light microscope and Ikaros Karyotyping imaging software v.6.3 (MetaSystems). A total of 160 metaphases was analyzed per cell line (40 cells per independent experiment, $n = 4$), except for fibroblasts from S5 and S12, for which 140 and 120 metaphases, respectively, were analyzed due to the limited availability of mitotic cells. Each metaphase was assessed for premature chromatid separation (PCS). PCS was defined as clearly visible split sister chromatids with a separated centromere. Metaphases were categorized as showing no PCS, mild PCS (≥1 chromosome with separated sister chromatids and a split centromere), and severe PCS (≥50% of chromosomes with separated sister chromatids and a split centromere).

## Statistics

Quantitative data were analyzed using GraphPad Prism 8 software (Dotmatics). The means of each independent experiment and cell line and the mean ± standard deviation (SD) or mean are shown. Statistical analysis was performed using one-way

ANOVA followed by Dunnett's post hoc test for multiple comparisons or an unpaired, two-tailed $t$ test. $p \leq 0.05$ was considered statistically significant (*$p \leq 0.05$, **$p \leq 0.01$, ***$p \leq 0.001$, and ****$p \leq 0.0001$).

## Results

### Identification of bi-allelic variants in WDHD1

We evaluated an 8-year-10-month-old girl with a history of intrauterine growth retardation (IUGR) who presented with MPD, delayed speech and language development, microphthalmia, and failure to thrive (Figure 1A; Tables 1 and S1). Whole-exome sequencing (WES) of the individual (S12) and healthy parents revealed the homozygous substitution c.1769−1G>C (GenBank: NM_007086.4) in *WDHD1*, affecting the invariant splice acceptor site in intron 14 (Figure 1B).

Through GeneMatcher[41] and national and international collaborations, we identified 16 additional subjects with variants in *WDHD1* and a broad spectrum of clinical features (Tables 1, 2 and S1; Figure S1). They range from MPD with normal cognitive function at the mild end to prenatal death of fetuses with IUGR, microcephaly, brain abnormalities, and/or additional organ abnormalities at the severe end of the clinical spectrum (Tables 1 and S1). In S1, a fetus with IUGR, microcephaly, heart abnormalities, and ambiguous genitalia, a rare homozygous *WDHD1* nonsense variant c.1117C>T (p.Arg373*) was identified (Tables 1, 2, and S1; Figure S1).

A total of ten subjects (S2–S9, S11, and S12) had the same homozygous intronic c.1769−1G>C variant, including three sibling pairs (Figure S1). This splice site variant is absent in the Genome Aggregation Database (gnomAD; v.4.1.0) (Table 2).[42] Trio WES or whole-genome sequencing (WGS) and/or segregation of the variant in healthy parents were performed in the families of S2–S9, S11, and S12 and confirmed the healthy parents as heterozygous carriers (Table 2; Figure S1). We mapped a 13.3-Mb haplotype on chromosome 14 that contained *WDHD1*. Similar haplotypes were identified in six subjects with the same homozygous *WDHD1* splice site variant (Figure S2).

S10, who died 2 days after birth, had the rare homozygous *WDHD1* variant c.2971G>T that is predicted to lead to the introduction of a premature stop codon in the mRNA (p.Glu991*) (Table 2). Trio WES in S13 identified the maternally inherited variant c.1341+5_1341+6inv and the heterozygous *de novo* variant c.3190−1G>A in *WDHD1* that were confirmed by Sanger sequencing (Figure 1B). Both variants are absent in the gnomAD. S14 had the same intronic inversion, c.1341+5_1341+6inv, as S13 on the paternal allele and a rare nonsense variant, c.3157C>T (p.Arg1053*), on the maternal allele. Trio WGS on S15 and parents revealed the compound heterozygous *WDHD1* variants c.1287del (p.Gln430Lysfs*37) and c.2339G>A (p.Arg780His) in the affected individual. While the 1-bp deletion is absent in the gnomAD, the missense variant has a worldwide allele frequency of 0.0005%. S16 and S17 were compound heterozygous for the rare intronic variants c.1341+5G>T and c.2531+1G>A and c.505−1G>A and c.1342−6T>A, respectively (Figures 1B and S1; Tables 1, 2, and S1). The *WDHD1* variants listed in the gnomAD were observed in the heterozygous, not the homozygous, state. All variants segregated among family members in a manner that is consistent with an autosomal recessive inheritance pattern (Table 2). Constraint data from gnomAD revealed a LOEUF (loss-of-function observed/expected upper bound fraction) of 0.923 for *WDHD1*, indicating that the gene is relatively tolerant to inactivating variants.[42] This finding is consistent with the autosomal-recessive inheritance pattern observed for the *WDHD1* variants.

In conclusion, the bi-allelic variants in *WDHD1* identified in our cohort include seven distinct intronic variants, present either in the homozygous or compound heterozygous state, or in *trans* with a nonsense variant. One subject was compound heterozygous for a frameshift and a missense variant, while another was homozygous for a predicted nonsense variant. In one subject, the identification of a maternally inherited intronic variant and a *de novo* splice site variant required confirmation of compound heterozygosity. Collectively, these findings suggest that most of the identified *WDHD1* variants are likely hypomorphic rather than complete loss-of-function alleles.

### Subjects with WDHD1 variants have microcephaly, intrauterine and postnatal growth retardation, and various other congenital anomalies that can lead to prenatal or early death

All of the subjects with *WDHD1* variants had intrauterine and/or postnatal growth retardation. The associated clinical spectrum can be divided into three groups based on the severity of clinical features and attained age (Tables 1 and S1). Group 1 comprises four fetuses, three of whom died *in utero*. Four presented with microcephaly and three with brain malformations, including hypoplasia of the cerebrum, cerebellum, or corpus callosum and/or lissencephaly. Two fetuses had microphthalmia. All four individuals had heart abnormalities. Gastrointestinal malformations, as well as renal, liver, and genital anomalies, were found in some fetuses. The six subjects in group 2 survived to term but died in the neonatal period. In five of the individuals, microcephaly (ranging from –5.4 to –12.1 $Z$ score) was observed, accompanied by various brain abnormalities. After birth, one subject had respiratory distress, and four had respiratory insufficiency. Other clinical features included hypotonia (all subjects), feeding difficulties in the neonatal period (2/5), microphthalmia (3/4) (Figure 1A), optic atrophy (1/4), retinal abnormalities (3/4), cardiac anomalies (4/6), gastrointestinal tract malformations (1/5), hypothyroidism (1/5), and hypoglycemia (4/4). Urogenital anomalies included uterine aplasia

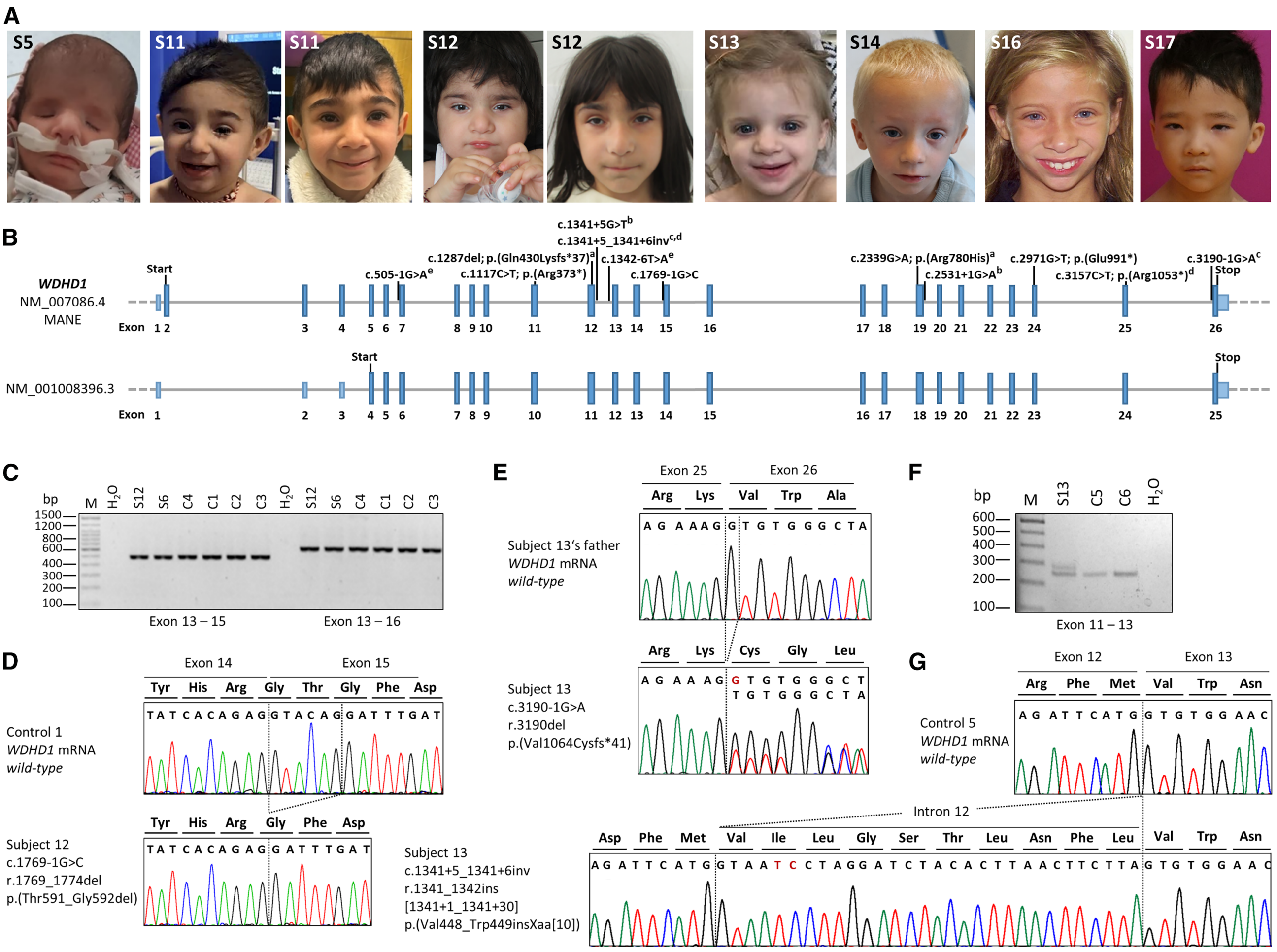


**Figure 1. Subjects with bi-allelic *WDHD1* variants and aberrantly spliced *WDHD1* mRNAs in subject-derived cells**

(A) Facial photographs of seven subjects with bi-allelic *WDHD1* variants. S5 is shown as a neonate. S11 is shown at 20 months (left) and at 5 years and 11 months (right). S12 is shown at 1 year (left) and at 8 years and 11 months (right). S13 is aged 2 years and 8 months, S14 is aged 3 years, S16 is aged 10 years, and S17 is aged 4 years and 10 months. Shared facial features include a small and/or narrow face, microphthalmia (in S5 and S12), a bulbous nose, retrognathia, and/or a pointed chin. S17 had blepharophimosis.

(B) Schematics of the *WDHD1* exon-intron structure of two transcript variants. The top image shows the MANE Select transcript (GenBank: NM_007086.4) and the bottom image an alternatively spliced transcript variant encoding a WDHD1 isoform with a shortened N terminus (GenBank: NM_001008396.3). Exons are shown as boxes, with coding regions in dark blue and untranslated regions in light blue. Introns are represented by gray lines. The location of start and stop codons and of variants is indicated by black lines. The variants c.1769−1G>C and c.2971G>T (p.Glu991*) were found in the homozygous state in S2–S9, S11, and S12 and in S10, respectively. Compound heterozygous variants are labeled (a–e) to indicate the two alleles found in each subject (a: S13, b: S14, c: S15, d: S16, and e: S17).

(C) Agarose gel showing RT-PCR amplicons generated from fibroblast-derived RNA (cDNA) of S6 and S12 with the homozygous c.1769−1G>C variant and of four control subjects (C1–C4). Two primer pairs targeting *WDHD1* were used, one spanning exons 13–15 (expected size: 495 bp) and one spanning exons 13–16 (expected size: 635 bp).

(D) Partial sequence electropherograms of *WDHD1* transcripts in fibroblast-derived RNA (cDNA) from S12 with the homozygous c.1769−1G>C variant compared to wild-type *WDHD1* transcripts (C1). The top image displays the *WDHD1* wild-type sequence at the exon 14-exon 15 junction. The bottom image shows *WDHD1* transcripts from S12, which contain a deletion of the first 6 bp of exon 15 (r.1769_1774del), predicted to result in loss of two amino acid residues at the protein level (p.Thr591_Gly592del). The black dotted lines indicate the aberrant splicing event.

(E) Partial sequence electropherograms showing the exon 25-exon 26 junction in *WDHD1* transcripts amplified from lymphocyte-derived RNA (cDNA) of S13's father and of S13 with the heterozygous variant c.3190−1G>A. The sequence of S13's father shows the *WDHD1* wild-type sequence at the exon 25-exon 26 junction (top). The c.3190−1G>A variant leads to loss of the first base of exon 26 (r.3190del; deleted guanine highlighted in red) that is predicted to result in a frameshift followed by the introduction of a stop codon (p.Val1064Cysfs*41). The black dotted lines indicate an aberrant splicing event.

(F) Agarose gel showing RT-PCR amplicons generated from lymphocyte-derived RNA (cDNA) of S13 with the heterozygous variant c.1341+5_1341+6inv and two control subjects (C5 and C6). The primer pair spans exons 11–13 (expected size: 251 bp).

(G) Partial sequence electropherograms showing *WDHD1* transcripts generated from lymphocyte-derived RNA (cDNA) from S13 with the heterozygous variant c.1341+5_1341+6inv and control subject 5 using RT-PCR followed by cloning. The sequence of control individual 5 shows the *WDHD1* wild-type sequence at the exon 12-exon 13 junction (top). The c.1341+5_1341+6inv variant leads to the insertion of the first 30 bp of intron 12, predicted to result in an *in-frame* insertion of ten amino acids (p.Val448_Trp449insValIleLeuGlySerThrLeuAsnPheLeu). The black dotted lines indicate aberrant splicing events.

**Table 1. Main clinical characteristics of 17 subjects with bi-allelic *WDHD1* variants**

| Feature | Number of subjects | | |
|---|---|---|---|
| | 4 (group 1), died prenatally or termination of pregnancy | 6 (group 2), died between the 2nd day of life and the 15th week | 7 (group 3), alive (aged 3–16 years) |
| Bi-allelic *WDHD1* variants | c.1117C>T, c.1769−1G>C | c.1769−1G>C, c.2971G>T | c.505−1G>T, c.1287del, c.1341+5G>T, c.1341+5_1341+6inv, c.1342−6T>A, c.2339G>A, c.2531+1G>A, c.3157C>T, c.3190−1G>A |
| Intrauterine growth retardation | 4/4 | 6/6 | 7/7 |
| Postnatal growth retardation | N/A | 5/5 | 7/7 |
| (Neonatal) respiratory insufficiency | N/A | 5/5 | 3/7 |
| (Primary) microcephaly | 4/4 | 5/5 | 7/7 |
| **Neurodevelopmental abnormality** | | | |
| Abnormal brain morphology, including hypoplasia of the cerebellum, cerebrum, and/or corpus callosum and/or abnormal cortical gyration | 3/4 | 5/6 | 0/7 |
| Abnormal cerebral ventricle morphology | 1/4 | 2/6 | 1/7 |
| Neonatal or generalized hypotonia | N/A | 6/6 | 3/7 |
| Motor delay | N/A | 1/1 | 6/7 |
| Delayed speech and language development | N/A | N/A | 5/7 |
| Mild developmental delay | N/A | N/A | 6/7 |
| High-pitched voice | N/A | N/A | 7/7 |
| **Abnormality of the face** | | | |
| Small face | N/A | 2/4 | 6/7 |
| Microphthalmia | 2/3 | 3/4 | 2/7 |
| Bulbous nose | 0/1 | 1/3 | 4/7 |
| Retrognathia | 1/1 | 4/5 | 3/7 |
| **Visual impairment** | | | |
| Optic atrophy | N/A | 1/4 | 3/7 |
| Abnormal heart morphology | 4/4 | 4/6 | 2/6 |
| **Abnormality of the endocrine system** | | | |
| (Congenital) hypothyroidism | N/A | 1/5 | 3/7 |
| **Abnormality of the digestive system** | | | |
| Abnormality of the gastrointestinal tract | 2/3 | 1/5 | 0/7 |
| Feeding difficulties (tube feeding) | N/A | 2/5 | 4/7 |
| **Abnormality of the liver** | | | |
| Abnormal liver morphology | 1/1 | 1/6 | 1/6 |
| Cirrhosis | 0/1 | 4/6 | 0/6 |
| Acute hepatic failure | 0/1 | 4/6 | 0/6 |
| Hepatic fibrosis | 1/1 | 0/6 | 0/6 |
| Giant cell hepatitis | 0/1 | 2/6 | 0/6 |

(*Continued on next page*)

Table 1. *Continued*

| Feature | Number of subjects | | |
|---|---|---|---|
| Elevated hepatic iron concentration | 0/1 | 4/6 | 0/6 |
| Elevated liver enzymes | 0/1 | 0/6 | 2/6 |
| **Abnormal homeostasis** | | | |
| (Neonatal) hypoglycemia | N/A | 4/4 | 3/6 |
| **Abnormality of the upper urinary tract** | | | |
| Abnormality of the kidney | 2/2 | 0/6 | 0/7 |
| Abnormality of the ureter | 0/2 | 1/6 | 0/7 |
| Abnormal reproductive system morphology | 1/3 | 2/6 | 4/7 |
| **Abnormality of the skeletal system** | | | |
| Abnormality of skeletal maturation | N/A | N/A | 4/7 |
| Hip dislocation | N/A | 1/6 | 6/7 |

N/A, not applicable.

(1/6), ambiguous genitalia (1/6), and abnormal ureter morphology (1/6). Skeletal abnormalities were observed in three of the six subjects. Five subjects had various liver abnormalities, including elevated hepatic iron concentration, impaired liver function, altered liver morphology, and/or cirrhosis. Four of these five subjects developed acute liver failure (ALF), which likely contributed to or caused their early death.

Group 3 includes seven individuals, aged between 3 and 16 years at last evaluation. Respiratory distress during the neonatal period was observed in three subjects and feeding difficulties during infancy in four. All subjects had microcephaly (ranging from –2.9 to –10.7 *Z*), short stature (ranging from –3.1 to –6.7 *Z*), and a high-pitched voice. Motor delay and mild developmental delay were present in six subjects. Five individuals had delayed speech and language development. A recognizable facial gestalt was not evident, although six had a small face, four a bulbous nose, and three retrognathia (Figure 1A). Two had microphthalmia (Figure 1A), and three had optic atrophy. Skeletal abnormalities included marked delay in bone age and hip dislocation as the most common features. Cryptorchidism, small genitalia, and/or other genital abnormalities were observed in four male subjects, and two of six had abnormal heart morphology. Endocrine abnormalities included hypothyroidism (3/7), growth hormone deficiency (3/7), and hypoglycemia (3/6). Among the six subjects evaluated for liver function and morphology, two showed transiently elevated liver enzymes and/or abnormal liver morphology. The mildest phenotype was observed in the 16-year-old female S16, who had MPD, pituitary hypoplasia, congenital hypothyroidism, and hip dislocation.

In conclusion, the common clinical presentation in the 17 subjects that fits MPD with or without additional neurodevelopmental and health issues provides strong evidence that bi-allelic variants in *WDHD1* cause an autosomal recessive human disorder. The associated clinical spectrum is broad, ranging from MPD without developmental delay to severe fetal phenotypes that may result in intrauterine fetal demise.

### Effects of intronic variants on WDHD1 transcripts

To investigate the consequences of the intronic variants c.505−1G>A, c.1341+5G>T, c.1341+5_1341+6inv, c.1342−6T>A, c.1769−1G>C, c.2531+1G>A, and c.3190−1G>A, we first assessed them using various splice site prediction programs.[44–47] All predicted loss or weakening of the directly affected or nearby canonical splice sites (Table 2), suggesting aberrant splicing of *WDHD1* pre-mRNA in the subjects carrying these variants. We also analyzed the four coding variants c.2971G>T, c.3157C>T, c.1287del, and c.2339G>A by the splice site prediction programs. The c.2971G>T change is predicted to create a new splice donor site within exon 24, while the canonical splice donor site at the exon 24-intron 24 boundary remains unaffected (Figure S3; Table 2). Possible splicing at the new donor site would result in the removal of 81 nucleotides (nt) from the mRNA (r.2970_3050del), potentially producing a WDHD1 protein lacking 27 amino acids (aa) (p.Glu991_Arg1017del). No biological specimens from S10 were available to assess the potential aberrant splicing of *WDHD1* pre-mRNA. For the three other coding variants analyzed, no alterations in canonical, new, or cryptic splice sites were predicted (Table 2).

For *WDHD1* transcript analysis and functional studies, we obtained primary fibroblasts from S5, S6, and S12–S14 and blood samples from S13, S13's parents, and S17. *WDHD1* variants were validated in fibroblast-derived DNA (Figure S4). We first investigated the effect of the homozygous variant c.1769−1G>C in intron 14 on *WDHD1* pre-mRNA splicing in fibroblasts from S6 and S12 and

Table 2. Bi-allelic *WDHD1* variants identified in 17 subjects

| Subject | Nucleotide change (GenBank: NM_007086.4) | Amino acid alteration (GenBank: NP_009017.1) | Segregation | Allele frequency (gnomAD v.4.1.0) | CADD v.1.6 | Splice site predictions | | | | | | | | |
|---|---|---|---|---|---|---|---|---|---|---|---|---|---|---|
| | | | | | | Splice site | SpliceSite Finder-like (range: 0–100) | | MaxEntScan (range: 0–16) | | NNSPLICE (range: 0–1) | | GeneSplicer (range: 0–21) | |
| | | | | | | | WT | Var | WT | Var | WT | Var | WT | Var |
| 1 | c.1117C>T (hom) | p.Arg373* | mat, pat | $8.70 \times 10^{-6}$ | 36 | c.1153 (CD) | 82.03 | 82.03 | 8.85 | 8.85 | 0.97 | 0.97 | 3.94 | 3.29 |
| 2 | c.1769−1G>C (hom) | p.Thr591_Gly592del | mat, pat[a] | absent | 34 | c.1769 (CA) | 85.14 | – | 8.05 | – | 0.99 | – | 5.95 | – |
| 3 | c.1769−1G>C (hom) | p.Thr591_Gly592del | mat, pat[a] | absent | 34 | c.1769 (CA) | 85.14 | – | 8.05 | – | 0.99 | – | 5.95 | – |
| 4 | c.1769−1G>C (hom) | p.Thr591_Gly592del | mat, pat[a] | absent | 34 | c.1769 (CA) | 85.14 | – | 8.05 | – | 0.99 | – | 5.95 | – |
| 5 | c.1769−1G>C (hom) | p.Thr591_Gly592del | mat, pat[a] | absent | 34 | c.1769 (CA) | 85.14 | – | 8.05 | – | 0.99 | – | 5.95 | – |
| 6 | c.1769−1G>C (hom) | p.Thr591_Gly592del | mat, pat | absent | 34 | c.1769 (CA) | 85.14 | – | 8.05 | – | 0.99 | – | 5.95 | – |
| 7 | c.1769−1G>C (hom) | p.Thr591_Gly592del | mat, pat | absent | 34 | c.1769 (CA) | 85.14 | – | 8.05 | – | 0.99 | – | 5.95 | – |
| 8 | c.1769−1G>C (hom) | p.Thr591_Gly592del | mat, pat[a,b] | absent | 34 | c.1769 (CA) | 85.14 | – | 8.05 | – | 0.99 | – | 5.95 | – |
| 9 | c.1769−1G>C (hom) | p.Thr591_Gly592del | mat, pat[a,b] | absent | 34 | c.1769 (CA) | 85.14 | – | 8.05 | – | 0.99 | – | 5.95 | – |
| 10 | c.2971G>T (hom) | p.Glu991* | not done | $6.285 \times 10^{-7}$ | 38 | c.2969 (ND) | – | 74.78 | – | 8.82 | – | 0.99 | – | – |
| 11 | c.1769−1G>C (hom) | p.Thr591_Gly592del | mat, pat | absent | 34 | c.1769 (CA) | 85.14 | – | 8.05 | – | 0.99 | – | 5.95 | – |
| 12 | c.1769−1G>C (hom) | p.Thr591_Gly592del | mat, pat[b] | absent | 34 | c.1769 (CA) | 85.14 | – | 8.05 | – | 0.99 | – | 5.95 | – |
| 13 | c.1341 + 5_1341+6inv (comp. het) | p.Val448_ Trp449insXaa[10] | mat | absent | N/A | c.1341 (CD) | 85.85 | 73.01 | 9.48 | 4.14 | 1.00 | 0.88 | 3.45 | – |
| | c.3190−1G>A (comp. het) | p.Val1064Cysfs*41 | *de novo* (on paternal allele) | absent | 33 | c.3190 (CA) | 72.88 | – | 6.19 | – | 0.61 | – | 6.26 | – |
| 14 | c.1341+5_1341+6inv (comp. het) | p.Val448_ Trp449insXaa[10] | pat | absent | N/A | c.1341 (CD) | 85.85 | 73.01 | 9.48 | 4.14 | 1.00 | 0.88 | 3.45 | – |
| | c.3157C>T (comp. het) | p.Arg1053* | mat | $1.967 \times 10^{-4}$ | 39 | c.3189 (CD) | 94.67 | 94.67 | 10.51 | 10.51 | 1.00 | 1.00 | 3.29 | 2.39 |
| 15 | c.1287del (comp. het) | p.Gln430Lysfs*37 | pat | absent | N/A | c.1341 (CD) | 85.85 | 85.85 | 9.48 | 9.48 | 1.00 | 1.00 | 3.45 | 3.28 |
| | c.2339G>A (comp. het) | p.Arg780His | mat | $4.680 \times 10^{-6}$ | 28 | c.2311 (CA) | 74.91 | 74.91 | 6.55 | 6.55 | 0.66 | 0.66 | 4.77 | 4.66 |
| 16 | c.2531+1G>A (comp. het) | ? | mat | $6.197 \times 10^{-7}$ | 33 | c.2531 (CD) | 81.58 | – | 8.91 | – | 0.99 | – | 0.66 | – |
| | c.1341+5G>T (comp. het) | ? | pat | $3.294 \times 10^{-5}$ | 16 | c.1341 (CD) | 85.85 | 73.46 | 9.48 | 5.05 | 1.00 | 0.84 | 3.45 | 1.18 |

(*Continued on next page*)

**Table 2. Continued**

| Subject | Nucleotide change (GenBank: NM_007086.4) | Amino acid alteration (GenBank: NP_009017.1) | Segregation | Allele frequency (gnomAD v.4.1.0) | CADD v.1.6 | Splice site predictions: Splice site | SpliceSite Finder-like (range: 0–100) WT | SpliceSite Finder-like Var | MaxEntScan (range: 0–16) WT | MaxEntScan Var | NNSPLICE (range: 0–1) WT | NNSPLICE Var | GeneSplicer (range: 0–21) WT | GeneSplicer Var |
|---|---|---|---|---|---|---|---|---|---|---|---|---|---|---|
| 17 | c.1342−6T>A (comp. het) | p.Val448Glnfs*8 | mat | $1.24 \times 10^{-5}$ | 17 | c.1342 (CA) | 85.49 | 79.72 | 9.13 | 7.90 | 0.98 | 0.84 | 8.03 | 5.11 |
| | c.505−1G>A (comp. het) | p.Thr169_Lys200del | pat | $1.33 \times 10^{-5}$ | 29 | c.505 (CA) | 78.87 | – | 5.75 | – | 0.80 | – | 4.44 | – |

Worldwide allele frequency of *WDHD1* variants in the gnomAD database (v.4.1.0)[42] is given. The functional impact of the variants was predicted by the CADD tool v.1.6. CADD is a unified deleteriousness scoring framework that integrates multiple genomic annotations to rank variants by predicted pathogenicity. Reported CADD scores are Phred-like rank scores based on the rank of that variant's score among all possible single-nucleotide variants of hg38, with 10 corresponding to the top 10%, 20 at the top 1%, and 30 at the top 0.1%. The higher the score, the more likely the variant has deleterious effects; the score range observed here is strongly supportive of pathogenicity, with all observed variants ranking above ~99% of all variants in a typical genome and scoring similarly to variants reported in ClinVar as pathogenic (~85% of which scored > 15).[43] Splice site prediction scores were predicted for wild-type sequences and the sequence with the variant using Alamut Visual Plus (v.1.4; 2021 SOPHIA GENETICS), which implements the following splice site prediction algorithms: SpliceSiteFinder-like, MaxEntScan, NNSPLICE (v.0.9), and GeneSplicer.[44–47] High scores indicate strong splice sites. CA, canonical acceptor splice site; CADD, Combined Annotation Dependent Depletion; CD, canonical donor splice site; comp. het, compound heterozygous; hom, homozygous; mat, variant identified in mother; N/A, not applicable; ND, new donor splice site; pat, variant identified in father; Var, variant; WT, wild type; –, no splice site detected.
[a]S2-S3, S4-S5, and S8-S9 are sibling pairs.
[b]Two healthy siblings carried the *WDHD1* variant in the heterozygous state (Figure S1).

four control individuals. We amplified RT-PCR products using primers located in *WDHD1* exons 13 and 15 (495 bp) and 13 and 16 (635 bp) and obtained amplicons of the expected size in S6 and S12 cells (Figure 1C). Sanger sequencing of the RT-PCR products revealed canonical splicing of exons 14–15 in control cells, while *WDHD1* transcripts in subject cells had a loss of the first 6 bp of exon 15 (r.1769_1774del), indicating the activation of a cryptic splice acceptor site in exon 15. The predicted consequence on protein level is an *in-frame* loss of threonine 591 and glycine 592 (p.Thr591_Gly592del) (Figure 1D). We next studied the maternally inherited variant c.1341+5_1341+6inv (intron 12) and the *de novo* variant c.3190−1G>A (intron 25) in S13 using RNA extracted from leukocytes. We amplified an RT-PCR product comprising exons 24–26 in S13 and her parents and directly sequenced the amplicons. Exon 25 was correctly spliced to exon 26 in the father's *WDHD1* transcripts, while two *WDHD1* transcripts were detected in S13: the wild-type transcript and an aberrant transcript lacking the first nucleotide (guanine) of exon 26 (r.3190del [p.Val1064Cysfs*41]) (Figure 1E). For the small intron 12 inversion c.1341+5_1341+6inv, we amplified an RT-PCR product with primers located in exons 11 and 13 and obtained an RT-PCR product of the expected 251 bp in addition to a larger amplicon of ~280 bp in S13 (Figure 1F). Subsequent cloning and Sanger sequencing of these PCR products demonstrated two *WDHD1* transcripts in S13, one canonically spliced and one aberrantly spliced transcript, which included the first 30 bp of intron 12, harboring the two inverted nucleotides, inserted between exons 12 and 13 (Figure 1G). The intronic insertion r.1341_1342ins[1341+1_1341+30] is predicted to cause an *in-frame* insertion of ten aa residues at the protein level (p.Val448_Trp449insXaa[10]).

We next aimed to determine whether the maternally inherited c.1341+5_1341+6inv inversion and the *de novo* c.3190−1G>A variant were present in *trans* in S13, thereby potentially underlying her phenotype. We first amplified *WDHD1* transcripts containing the 30-nt insertion from intron 12 through to the 3′ untranslated region (UTR) of the mRNA from S13's RNA (Figure S5A). We obtained the expected 2,286-bp RT-PCR product in S13, which was absent in the RNA of control individual 1 (Figure S5B). Sequencing of this amplicon revealed the intron 12-exon 13 junction along with canonically spliced exons 25 and 26 (Figure S5B), suggesting that the c.3190−1G>A variant is located on the paternal allele. To confirm the allelic phase of the two variants, we next used a primer combination to amplify *WDHD1* transcripts with or without the intron 12 insertion through to the final exon 26. The PCR products were cloned, and the inserts of 54 individual clones were sequenced. Among these, 27 clones (50%) contained the intron 12 insertion and the wild-type exon 25- exon 26 junction, 19 clones (35%) had the wild-type exon 12-exon 13 junction along with the r.3190del variant in exon 26, and 8 clones (15%-) corresponded to the *WDHD1* reference transcript

(Figure S5C). These results demonstrate that the two *WDHD1* variants are in *trans* in S13 (Figure S5A) and that residual wild-type *WDHD1* mRNA is expressed from one or both alleles.

S14 carried the intronic variant c.1341+5_1341+6inv and the nonsense variant c.3157C>T (p.Arg1053*) in the compound heterozygous state (Table 2). To confirm the same insertion in *WDHD1* transcripts due to the small inversion in intron 12 as in S13, we isolated RNA from S14's primary fibroblasts and amplified an RT-PCR product using a forward primer in exon 11 and a reverse primer in intron 12 (Figure S6A). We obtained a 406-bp amplicon from RNA of S14 and S13 (as positive control) but not from control cells (Figure S6B). Sequencing of the RT-PCR product confirmed that exon 12 was spliced to the first 30 bp of intron 12 in S14 cells (Figure S6C). To assess whether the nonsense variant in the penultimate exon (exon 25) causes nonsense-mediated mRNA decay (NMD) of *WDHD1* transcripts, we amplified a 470-bp RT-PCR product using primers located in exons 24 and 26 from RNA of S14 and control cells (Figures S6A and S6B). Direct sequencing of the amplicons showed the reference exon 25 in *WDHD1* transcripts of control 1 cells, while S14 cells had nearly equal levels of both the wild-type transcript with r.3157C and the transcript with the r.3157U variant (Figure S6D). These data suggest that *WDHD1* mRNAs containing the nonsense variant r.3157C>U are likely not subject to NMD in S14 fibroblasts.

S17 was compound heterozygous for the *WDHD1* variants c.505−1G>A in intron 6 and c.1342−6T>A in intron 12 (Table 2). We first investigated the effect of the c.505−1G>A change on *WDHD1* pre-mRNA splicing by amplifying an RT-PCR product with primers located in exons 6 and 9 using leukocyte-derived RNA (cDNA) from S17 and two control individuals. In addition to the expected 264-bp amplicon, we generated an RT-PCR product of ∼170 bp in S17 that was absent in control individuals (Figure S7A). Direct sequencing of S17's amplicons revealed two *WDHD1* transcripts, the wild-type mRNA and a transcript lacking exon 7 (Figure S7B). This finding was confirmed by cloning and Sanger sequencing of the RT-PCR products (Figure S7C). Thus, the c.505−1G>A variant leads to skipping of exon 7 in the *WDHD1* mRNA (r.505_600del) that is predicted to cause a loss of 32 aa residues at the protein level (p.Thr169_Lys200del). We finally analyzed the consequence of the c.1342−6T>A variant by amplifying an RT-PCR product spanning exons 12 to 14 in S17 and control individuals. Again, we detected two amplicons in S17: the expected 427-bp amplicon and a smaller product. In control individuals, we observed a single amplicon of the expected size (Figure S7D). Sanger sequencing of S17's RT-PCR products, as well as cloning followed by sequencing, identified skipping of exon 13 in *WDHD1* mRNAs (Figures S7E and S7F). Exon 13 skipping (r.1342_1526del) is predicted to result in a frameshift and a premature termination codon at the protein level (p.Val448Glnfs*8). Based on the relative intensity of the two smaller RT-PCR products corresponding to each skipped exon in S17 (Figures S7A and S7D), we assume that exon 7 skipping is more pronounced than exon 13 skipping in S17's leukocytes. This is consistent with the predicted effects of the c.505−1G>A and c.1342−6T>A variants based on splice site prediction programs (Table 2). We next assessed *WDHD1* mRNA levels in fibroblasts from S5, S6, S13, and S14 and three control subjects using two different primer sets in real-time qPCR. Among the control fibroblasts, *WDHD1* transcript levels ranged from 66% to 136% relative to the mean *WDHD1* transcript level of all control cells (Figure 2A). In fibroblasts from the four subjects, *WDHD1* mRNA levels ranged from 59% to 92%. Compared with control 1 cells, almost all *WDHD1* mRNA levels in subject fibroblasts were significantly reduced (Figure 2A). The data demonstrate considerable variability in *WDHD1* mRNA levels across both subject and control fibroblasts, with no evidence supporting a substantial contribution of NMD in subject cells, especially in fibroblasts from S14.

## Bi-allelic variants in WDHD1 reduce protein levels but do not result in a complete protein loss

*WDHD1* expresses two transcript variants through alternative splicing, encoding a larger isoform of 1,129 aa (GenBank: NP_009017.1) with a molecular weight of ∼126 kDa (corresponding to GenBank: NM_007086.4 in Figure 1B) and a shorter isoform of 1,006 aa (GenBank: NP_001008397.1) with a shortened N terminus and a molecular mass of ∼113 kDa (corresponding to GenBank: NM_001008396.3 in Figure 1B). WDHD1 has a conserved domain structure, with an N-terminal β-propeller domain (aa 1–330), a SepB domain (aa 420–850) in the central region, and a high-mobility group (HMG)-box domain (aa 1017–1076) at the C terminus (Figure 2B). The ring-like β-propeller is formed by seven consecutive WD40 repeats and is required for protein-protein interactions. The SepB domain mediates homotrimerization of WDHD1, and the HMG domain is responsible for WDHD1's interaction with DNA polymerase α-primase.[7,10,48] To investigate whether WDHD1 proteins are produced from mRNAs expressed in fibroblasts from S5, S6, and S12–S14, we performed immunoblotting with an anti-WDHD1 antibody using whole-cell lysates from subject and control cells. The antibody readily detected both WDHD1 isoforms in control fibroblasts, with the higher-molecular-weight isoform being more prominent than the lower-molecular-weight one (Figures 2C and S8). Consistent with *WDHD1* mRNA levels, WDHD1 protein levels in control cells were highly variable, ranging from 55% to 145% (Figure 2D). In fibroblasts from S5, S6, and S12 homozygous for the c.1769−1G>C variant, two very faint bands were detected, most likely corresponding to the larger and shorter WDHD1 variant isoforms lacking threonine 591 and glycine 592 within the SepB domain (Figures 2C, left, and S8). Total WDHD1 protein levels were significantly reduced in fibroblasts from S5, S6,

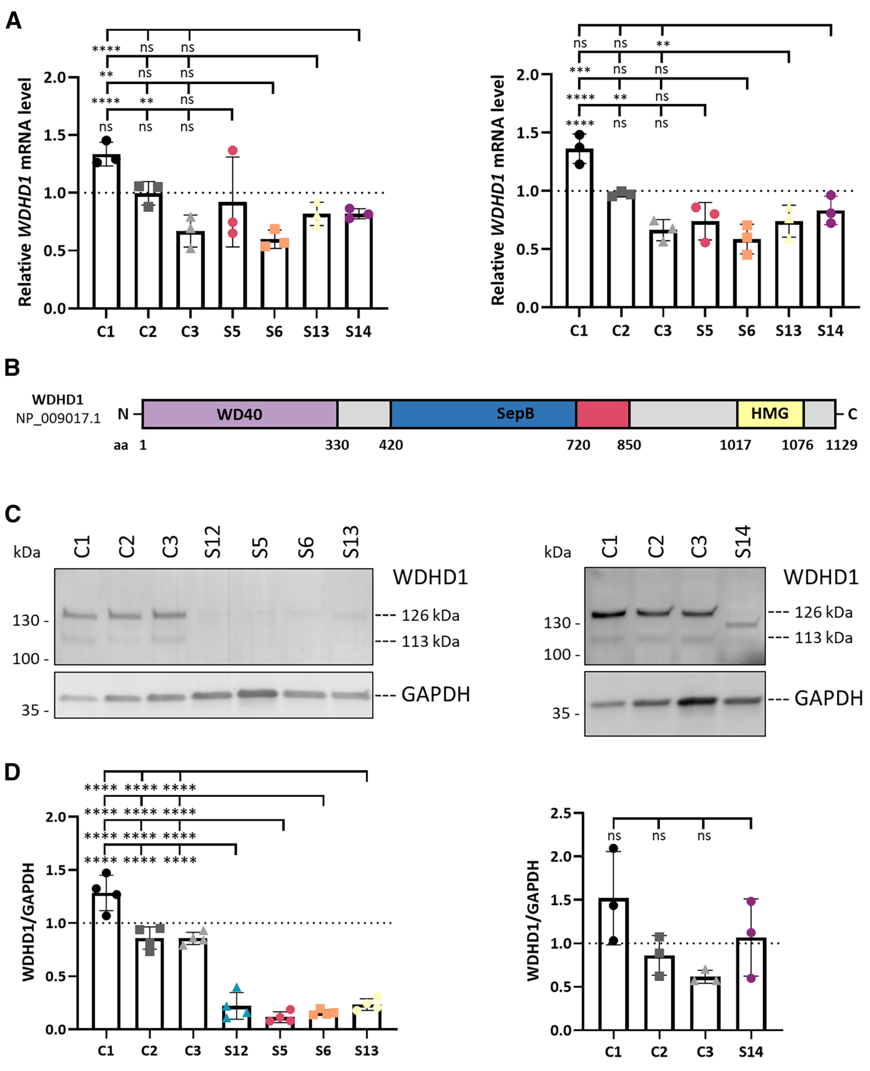


**Figure 2. Unchanged *WDHD1* mRNA levels and reduced WDHD1 protein in subject-derived fibroblasts**

(A) Relative quantification of *WDHD1* transcript levels in fibroblast-derived cDNA (RNA) from S5, S6, S13, S14, and three control individuals. Two primer pairs targeting *WDHD1* exons 13–14 (left) and 15–16 (right) were used. *GAPDH* mRNA served as an internal control, and data are normalized to *GAPDH* levels. The mean ± SD of three independent experiments, each performed in triplicate, is shown.

(B) Schematic representation of the domain architecture of human WDHD1 (GenBank: NP_009017.1). The protein consists of 1,129 aa residues and contains an N-terminal WD40 repeat domain (aa 1–330, WD40: tryptophan-aspartic acid repeats, lilac); a central SepB domain (aa 420–850, SepB: homologous to the bacterial septum site-determining protein B), which consists of a β-propeller subdomain (aa 420–720, blue) and a helical bundle (aa 721–850, red); and a C-terminal high-mobility group (HMG)-box region (aa 1017–1076, yellow).

(C) Representative immunoblot of fibroblast lysates from S12, S5, S6, S13, and three control individuals is shown (left). S14 fibroblasts were analyzed in a separate experiment with their own set of control cells (right). Levels of endogenous WDHD1 isoforms of 126 (GenBank: NP_009017.1) and 113 (GenBank: NP_001008397.1) kDa, as well as the C-terminally truncated proteoform in S14 cells, were assessed using the indicated antibodies, with GAPDH serving as a loading control.

(D) Quantification of the immunoblot data. Fluorescent band intensities were quantified using a ChemiDoc imaging system. Band intensities were normalized to GAPDH and subsequently to the mean of control cells. The mean ± SD of four (left) and three (right) independent experiments is shown.

Statistical significance was determined by one-way ANOVA followed by Dunnett's multiple comparisons test. C1–C3, control fibroblasts; ns, not significant; S5, S6, and S12–S14, subject fibroblasts. *$p \leq 0.05$, **$p \leq 0.01$, ***$p \leq 0.001$, and ****$p \leq 0.0001$.

and S12, ranging from 15% to 20% of the levels observed in control cells (Figures 2D, left, and S8). In S13 fibroblasts with the compound heterozygous variants c.1341+5_1341+6inv and c.3190−1G>A, both WDHD1 isoforms were barely detectable (Figures 2C, left, and S8), with total WDHD1 protein reduced to ~22% of control levels (Figures 2D, left, and S8). As a small proportion of *WDHD1* wild-type transcripts was identified in S13 cells (Figure S5C), the two WDHD1 isoforms may be derived from these reference mRNAs. In S14 fibroblasts with compound heterozygosity of the variants c.1341+5_1341+6inv and c.3157C>T (p.Arg1053*), we observed similarly faint bands corresponding to the large and small WDHD1 isoforms as in other subject cells. However, we detected a prominent third WDHD1 isoform migrating at ~120 kDa (Figures 2C, right, and S8). This isoform was consistent with the predicted C-terminally truncated WDHD1$^{Arg1053*}$ protein (calculated molecular mass of ~117 kDa). Total levels of all three WDHD1 isoforms in S14 fibroblasts were similar to those in control cells (Figure 2D, right).

In conclusion, bi-allelic variants in *WDHD1* markedly reduce WDHD1 protein levels in fibroblasts from four subjects but do not result in a complete absence of the protein. WDHD1$^{Thr591_Gly592del}$ produced from aberrantly spliced *WDHD1* mRNAs (r.1769_1774del) likely shows intrinsic instability. S14 cells had total WDHD1 levels similar to those of control cells and produced an additional stable isoform, most likely corresponding to WDHD1$^{Arg1053*}$, which lacks the C-terminal 77 aa, including 24 residues from the HMG domain.

## Bi-allelic WDHD1 variants impair cell proliferation and delay G1/S transition

As knockdown of *WDHD1* in HeLa cells and chicken DT40 cells slowed cell growth,[15,16] we determined the growth

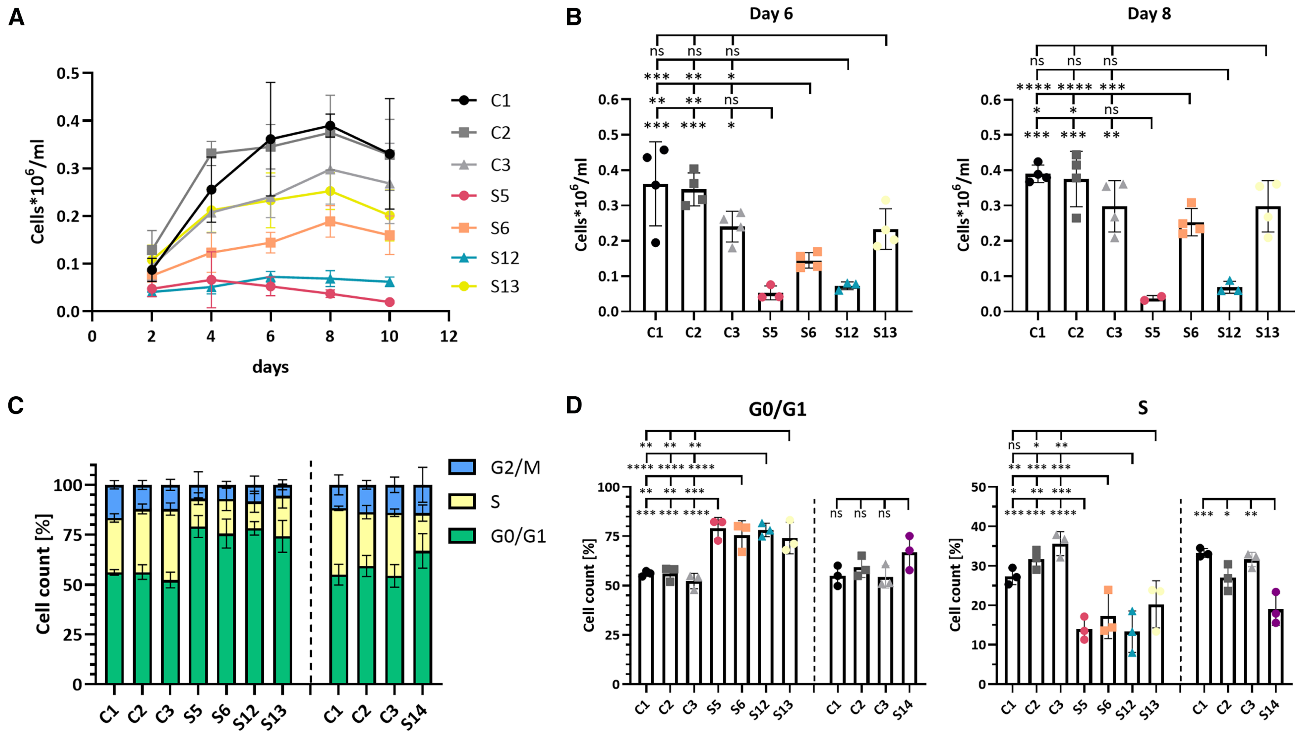


**Figure 3. Reduced proliferation and impaired G1-to-S transition in fibroblasts with bi-allelic *WDHD1* variants**
(A) Proliferation of fibroblasts from S5, S6, S12, S13, and three control individuals. Equal numbers of cells ($1 \times 10^5$) were seeded and counted at 2-day intervals for 10 days. Cell growth curves show the mean ± SD of three independent experiments.
(B) Quantification of the data shown in (A) for days 6 (left) and 8 (right).
(C) Cell cycle distribution derived from DNA (propidium iodide) flow cytometry. The proportions of cells in G0/G1 (green), S (yellow), and G2/M (blue) phases were normalized to 100%, and the percentage of cells in each phase is shown. Data represent the mean ± SD of three independent experiments. S14 fibroblasts were analyzed in a separate experiment with their own set of control cells.
(D) Quantification of the data shown in (C) for cells in the G0/G1 (left) and the S (right) phases.
Statistical significance was determined by one-way ANOVA followed by Dunnett's multiple comparisons test. C1–C3, control fibroblasts; ns, not significant; S5, S6, and S12–S14, subject fibroblasts. *$p \leq 0.05$, **$p \leq 0.01$, ***$p \leq 0.001$, and ****$p \leq 0.0001$.

rate of fibroblasts from S5, S6, S12, S13, and three control subjects (Figure 3A). Fibroblasts from three subjects showed significantly reduced proliferation compared to control cells, with the most pronounced differences observed on days 6 and 8 (Figure 3B). On day 6, fibroblasts from S5, S6, and S12 displayed a 2.2- to 6.1-fold reduction in cell number relative to control cells and, on day 8, a 1.4- to 8.7-fold reduction. In contrast, the slight reduction in cell number in S13 fibroblasts on days 6 and 8 did not reach statistical significance when compared with control fibroblasts (Figure 3B).

Silencing of *WDHD1* in different human cell lines causes cell cycle defects, such as a G1/S phase arrest in WDHD1-depleted HeLa cells.[11,13,15] We therefore performed cell cycle analysis in asynchronous fibroblasts from S5, S6, S12–S14, and three control subjects (Figures 3C and 3D). Fibroblasts from S14 were acquired at a later stage of the study, necessitating three additional experiments to complete the cell cycle analysis (Figure 3C, right). In control fibroblasts, 52%–59% of cells were in the G0/G1 phase, 27%–36% were in the S phase, and 12%–16% were in the G2/M phase (Figures 3C and S9). We found that subject-derived fibroblasts, except for S14 cells, accumulated in G0/G1 (67%–79%) and had significantly fewer cells in S phase (13%–20%) compared with control fibroblasts (Figures 3C and 3D). For cells from S5, S6, S12, and S13, we identified a trend toward fewer cells in the G2/M phase (6%–8%) compared with control cells, whereas S14 fibroblasts had a similar proportion of cells in the G2/M phase (~14%) as control fibroblasts (12%–16%) (Figure S9). Together, our data show that bi-allelic *WDHD1* variants reduce cell proliferation and cause an accumulation of subject-derived fibroblasts in the G0/G1 phase, suggesting that the G1-to-S progression is impaired.

## Bi-allelic WDHD1 variants impair DNA replication processes

WDHD1 plays a central role in DNA replication, and WDHD1 depletion slows replication fork progression.[16] This finding, together with the reduced proliferation rate and lower S-phase proportion in subject fibroblasts, raises the question of how DNA replication is affected by bi-allelic *WDHD1* variants. We therefore analyzed replication processes in fibroblasts from S5, S6, S12–S14, and three control individuals using the DNA fiber assay (Figure 4A). A significant slowing in replication fork progression, relative to the mean track length of

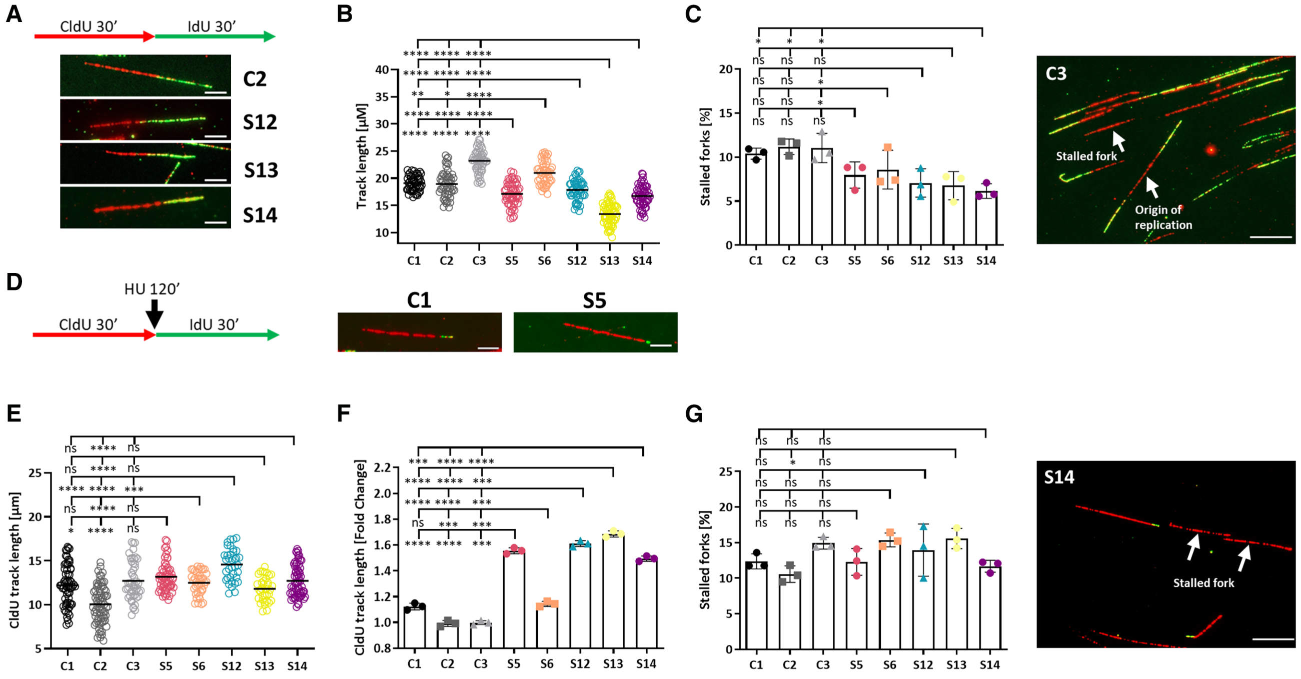


**Figure 4. Perturbation of replication processes in fibroblasts with bi-allelic *WDHD1* variants**
(A) Treatment scheme of the DNA fiber assay with CldU and IdU labeling of cells for 30 min each, followed by immunofluorescence detection (CldU: red, IdU: green). A minimum of 50 DNA replication tracks per experiment were analyzed for each cell line in three independent experiments. Representative images of DNA fibers from fibroblasts of C2 and S12–S14 are shown. Scale bar, 5 μm.
(B) Replication track length of fibroblasts from S5, S6, S12–S14, and three control individuals. Individual data points obtained from three independent experiments are shown, with the mean value indicated by the black line.
(C) Quantification of replication structures was performed, including origins of replication (ORIs) and stalled replication forks, indicated by white arrows in the representative DNA fiber image of C3 cells (right). Scale bar, 10 μm. The proportions of stalled replication forks were quantified relative to actively replicating forks and are presented as percentages. A minimum of 50 structures per experiment was analyzed for each cell line in three independent experiments.
(D) Scheme illustrates CldU and IdU labeling of cells for 30 min each, with a 2-h incubation with hydroxyurea (HU) in between to halt DNA replication (left). At least 50 DNA replication tracks per experiment were analyzed for each cell line in three independent experiments. Representative images of DNA fibers under HU-treated conditions are shown for fibroblasts of C1 (middle) and S5 (right). Scale bar, 5 μm.
(E) CldU replication track length of fibroblasts from S5, S6, S12–S14, and three control individuals under HU-treated conditions. Individual data points obtained from three independent experiments are shown, with the mean value indicated by the black line.
(F) Effect of HU treatment on the length of CldU tracks in fibroblasts from S5, S6, S12–S14, and three control individuals after normalization to the track length of untreated cells. Quantification of individual datasets is shown as fold change relative to the respective untreated cell line.
(G) Quantification of stalled replication forks under HU-treated conditions, as indicated by white arrows in the representative DNA fiber image of S14 cells (right). Scale bar, 10 μm. The proportions of stalled replication forks were quantified relative to actively replicating forks and are presented as percentages (left). A minimum of 50 structures per experiment were analyzed for each cell line in three independent experiments.
The mean ± SD of three independent experiments is shown (B, C, and E–G). Statistical significance was determined by one-way ANOVA followed by Dunnett's multiple comparisons test. C1–C3, control fibroblasts; ns, not significant; S5, S6, and S12–S14, subject fibroblasts. *$p \leq 0.05$, **$p \leq 0.01$, ***$p \leq 0.001$, and ****$p \leq 0.0001$.

the three control fibroblasts, was observed in four of the five subject fibroblasts, with S13 cells showing the most pronounced reduction in replication track length of ~34% compared to control cells (Figure 4B). S6 fibroblasts maintained a replication track length comparable to the mean of control cells (Figure 4B). The percentage of origins of replication was similar in subject and control cells: 6.0%–10.2% and 7.2%–11.5%, respectively (Figure S10A). Similarly, no difference in fork asymmetry was observed between control and subject fibroblasts (Figure S10B). In contrast, a trend toward a lower proportion of stalled replication forks was detected in subject fibroblasts, with 6.1%–8.6%, compared to control cells with 10.4%–11.2% (Figure 4C), suggesting that bi-allelic *WDHD1* variants negatively affect the control of replication fork progression.

To examine the effect of bi-allelic *WDHD1* variants on stalled replication forks, we repeated the DNA fiber assay with HU treatment of fibroblasts between the CldU and IdU pulses. HU depletes deoxynucleoside triphosphates required for DNA replication and induces fork stalling (Figures 4D and S10C).[49,50] To analyze the stability of stalled forks, we measured the CldU signal length. In control cells, we observed no change in track

length after HU treatment (Figure 4E), indicating immediate fork stalling and stabilization of synthesized DNA. In the five HU-treated subject cells, the CldU-labeled track lengths were similar to those of HU-treated control cells (Figure 4E). However, direct comparison of CldU track lengths between HU-treated and untreated subject cells revealed a significant increase in CldU track length following HU treatment in subject cells, except S6 cells, with the strongest effect (~1.7-fold increase) observed in S13 cells (Figure 4F). These data suggest dysregulated DNA replication in fibroblasts with bi-allelic *WDHD1* variants following exogenous replication stress. To determine replication fork restart after mild replication stress, we measured the IdU signal length at recovered replication forks after HU removal. We did not find consistent differences between subject and control cells (Figure S10D). As depletion of WDHD1 in HCT116 cells increased the fraction of forks that failed to recover from HU treatment,[24] we next quantified stalled forks in HU-treated fibroblasts (Figure 4G). A comparable proportion of stalled forks under HU-treated conditions was observed in subject (11.7%–15.6%) and control (10.5%–14.9%) cells (Figure 4G). However, relative to the untreated conditions, we found a compromised fork recovery in all subject cells, with the proportion of stalled forks increasing 1.6- to 2.1-fold under HU-treated conditions. In contrast, control cells showed little or no increase in the proportion of stalled forks, with a fold change of 0.9–1.2 (Figure S10E). Compromised fork recovery after HU treatment in subject cells remained significant even after a prolonged 45-min fork restart period (IdU labeling) (Figure S10F). Taken together, the data demonstrate that bi-allelic *WDHD1* variants impact multiple aspects of DNA replication, thereby emphasizing the critical role of WDHD1 in regulating DNA replication, especially in maintaining replication fork progression and stability.

### Fibroblasts with bi-allelic WDHD1 variants show an increase in DNA damage and abnormal nuclear structure

The replication defects in fibroblasts with bi-allelic *WDHD1* variants may lead to genomic instability and an increase in DNA damage. Consistent with this, *WDHD1* knockdown in HeLa cells resulted in an increase in γH2AX, a marker of DNA double-stranded breaks,[51] which appears as distinct γH2AX foci within the nuclei.[15] Similarly, *WDHD1*-depleted DT40 cells show γH2AX foci accumulation.[16] We next analyzed the presence and number of spontaneous γH2AX foci in fibroblasts from S5, S6, S12, S13, and three control individuals by IF (Figure 5A). While 5%–9% of control cells had γH2AX foci, between 10% and 26% of fibroblasts with bi-allelic *WDHD1* variants exhibited γH2AX foci. Statistical significance was observed for S5 and S12 cells (Figure 5B, left). We next determined the number of foci observed per cell and identified a trend toward an increased number of cells with foci in subject cells compared with control cells. The mean of foci per cell ranged from 0.05 to 0.13 in control cells and from 0.15 to 0.55 in subject cells, reaching statistical significance for S5 and S12 cells (Figure 5B, right). Collectively, the data suggest elevated spontaneous DNA damage in fibroblasts with bi-allelic *WDHD1* variants under basal culture conditions.

During IF analysis, particularly using DAPI staining, we noted aberrant nuclear morphology in fibroblasts with bi-allelic *WDHD1* variants. We investigated this phenotype further and categorized cells as having multilobed nuclei, micronuclei, and nuclei with grossly abnormal morphology (Figure 5A). A higher number of cells with abnormal nuclear structure was observed in fibroblasts from subjects compared with control cells. A trend toward an increased proportion of cells with multilobed nuclei (8%–14%) was found in S5, S6, and S12 cells compared with control cells (1%–2%), reaching statistical significance for S12 cells (Figure 5C, top left). Similarly, more micronuclei were found in S5, S6, and S12 cells (2%–6%) than in control cells (0%–1%), with statistical significance observed for S5 fibroblasts (Figure 5C, top right). For both parameters, S13 fibroblasts showed percentages similar to control cells (Figure 5C, top right and left). The most consistent and statistically significant difference observed was a grossly abnormal nuclear morphology in 28%–73% of subject cells compared to 3%–13% of control cells (Figure 5C, bottom left).

To confirm aberrant nuclear morphology and investigate possible nuclear envelope changes, we stained fibroblasts from two selected subjects (S5 and S14, the latter cells not included in the previous IF analysis) and one control individual for lamin B1, a marker of the nuclear lamina. While lamin B1 was uniformly distributed in nuclei of control cells, we found lamin B1-positive aberrant structures in nuclei of both subject cells. We detected wrinkling and folding of the nuclear lamina and distinct lamin B1-positive foci in the nuclei of fibroblasts with bi-allelic *WDHD1* variants, which were rarely, if at all, observed in control fibroblasts (Figure S11). We next determined the nuclear size and found a 1.4- to 1.5-fold increase in nuclear area in S5, S6, and S12 cells relative to control cells. In S13 cells, we detected a statistically non-significant increase of ~1.2-fold (Figure 5C, bottom right). Finally, we determined the proportion of regular-sized and enlarged nuclei containing γH2AX foci and identified a trend toward a higher number of enlarged nuclei with γH2AX foci in S5 (~48%) and S12 (~54%) fibroblasts compared to control cells (4%–17%) (Figure S12). Altogether, the observed morphological changes in the nuclei are in line with impaired DNA replication, reduced cell proliferation, and cell cycle abnormalities in fibroblasts with bi-allelic *WDHD1* variants.

### Fibroblasts with bi-allelic WDHD1 variants show a significant increase in premature sister chromatid separation

WDHD1 is involved in the establishment of sister chromatid cohesion, as depletion of wdhd1 in *Xenopus* egg

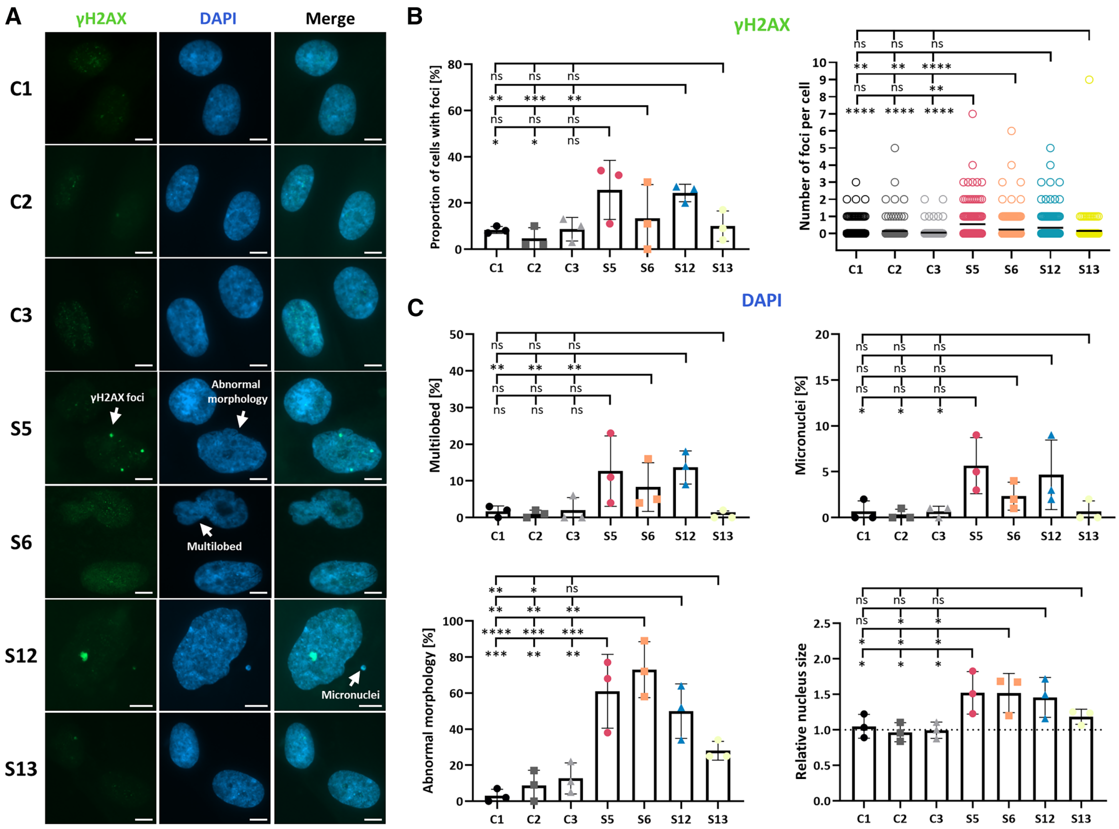


**Figure 5. Increased DNA damage and altered nuclear morphology in fibroblasts with bi-allelic *WDHD1* variants**
(A) Immunofluorescence analysis of fibroblasts from S5, S6, S12, S13, and control individuals. Cells were stained with an anti-γH2AX antibody, followed by an Alexa Fluor 488-conjugated secondary antibody (green), and counterstained with DAPI (blue). Representative images from three independent experiments are shown. Exemplary nuclei showing γH2AX foci, abnormal morphology, multilobed nuclei, and micronuclei are indicated by white arrows. Scale bar, 5 μm.
(B) Quantitative analysis of the proportion of cells with γH2AX foci (left) and numbers of γH2AX foci observed per cell (right) in control and subject fibroblasts.
(C) Quantitative analysis of the nuclear morphology. Cells were scored as having multilobed nuclei (top left), micronuclei (top right), and nuclei with grossly abnormal morphology (bottom left). The nuclear area was measured in control and subject cells and normalized to the mean of control cells (bottom right).
The mean ± SD of three independent experiments in control and subject cells is shown. Statistical significance was determined by one-way ANOVA followed by Dunnett's multiple comparisons test. C1–C3, control fibroblasts; ns, not significant; S5, S6, S12, and S13, subject fibroblasts. *$p \leq 0.05$, **$p \leq 0.01$, ***$p \leq 0.001$, and ****$p \leq 0.0001$.

extracts results in loosening of sister chromatid pairing.[17,18] Similarly, the *S. cerevisiae ctf4Δ* mutant showed a significant increase in premature sister chromatid separation.[26,52] In humans, PCS is a chromosomal configuration characterized by the separation of sister chromatids with split centromeres in a metaphase.[53] To investigate if fibroblasts with bi-allelic *WDHD1* variants show increased PCS, we arrested subject and control fibroblasts in mitosis and analyzed metaphase spreads. Metaphases were classified into three categories: no PCS, mild PCS (more than one chromosome displaying separated sister chromatids with split centromeres), and severe PCS (more than 50% of chromosomes affected) (Figures 6A and S13). In fibroblasts from S5, S6, S12, and S13, the proportion of cells without PCS was reduced to 37%–56%, compared to 65%–85% in control cells. Statistical significance was observed for S5 and S12 cells when compared with control 1 and 3 cells (Figure 6B, left). The proportion of cells with mild PCS was similar in subject (30%–35%) and control (11%–22%) cells (Figure 6B, middle). Severe PCS was found in 3%–13% of control cells and 11%–28% of subject-derived fibroblasts. Statistical significance was reached for S5, S12, and S13 cells when compared with at least one control cell line (Figure 6B, right). Together, fibroblasts with bi-allelic *WDHD1* variants show a trend toward increased PCS, suggestive of impaired sister chromatid cohesion.

## Discussion

Here, we report bi-allelic variants in *WDHD1* in 17 subjects with MPD and additional clinical features of variable severity. All subjects had severe IUGR and microcephaly (Table 1). The phenotype observed in ten subjects homozygous for the c.1769−1G>C variant was

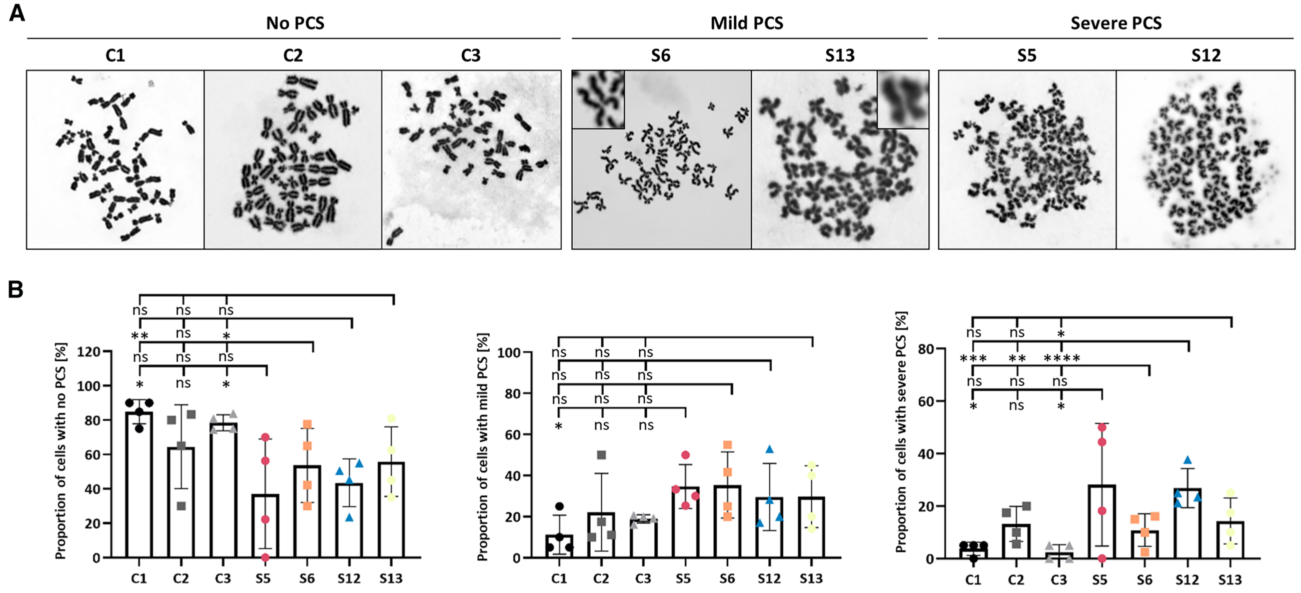


**Figure 6. Increased premature chromatid separation in fibroblasts with bi-allelic *WDHD1* variants**

(A) Fibroblasts were arrested in mitosis, and metaphase spreads were prepared. Each metaphase (i.e., the complement of chromosomes from one cell) was classified as showing no premature chromatid separation (PCS), mild PCS (>1 chromosome showing two separate chromatids with a split centromere), or severe PCS (>50% of chromosomes showing two separate chromatids with a split centromere). Representative images of each category are shown.

(B) Quantification of metaphases categorized as showing no PCS (left), mild PCS (middle), or severe PCS (right) in control fibroblasts and fibroblasts derived from S5, S6, S12, and S13.

The mean ± SD of four independent experiments is shown. Statistical significance was determined by one-way ANOVA followed by Dunnett's multiple comparisons test. C1–C3, control fibroblasts; ns, not significant; S5, S6, S12, and S13, subject fibroblasts. *$p \leq 0.05$, **$p \leq 0.01$, ***$p \leq 0.001$, and ****$p \leq 0.0001$.

broad, ranging from fetal lethality to MPD with mild to moderate developmental delay and additional clinical features. In the four fetuses, including three with the homozygous c.1769−1G>C variant, severe malformations of multiple organs likely contributed to intrauterine death in three of them (group 1 in Table 1). Among the five subjects with the homozygous c.1769−1G>C variant who survived to birth but died as neonates, all had severe hepatic abnormalities, with ALF as the cause of death in four (group 2 in Table 1). These findings implicate bi-allelic *WDHD1* variants as a potential genetic cause of ALF in genetically unsolved cases with IUGR and microcephaly. Interestingly, in primary human hepatocytes and liver samples from individuals with acetaminophen-induced ALF, an increase in DNA damage and cell cycle alterations was identified.[54] Hepatocytes are polyploid, containing nuclei with 2n, 4n, or 8n DNA content. Although both diploid and polyploid hepatocytes contribute to liver growth and regeneration, diploid hepatocytes play an important role in liver homeostasis.[55] Liver samples from acetaminophen-induced ALF showed a reduction in the diploid cell population and an increase in the octoploid cell population compared to healthy liver samples. Moreover, aneuploid cell populations were more prevalent in ALF samples, indicating that hepatocytes from ALF livers fail to complete the cell cycle.[54] Consistent with these findings, oxidative stress triggers a DNA damage response followed by atypical cell cycle progression, leading to pathological polyploidization of hepatocytes.[56] Furthermore, replication stress followed by DNA damage is a hallmark of proliferating hepatocytes in a mouse model of non-alcoholic steatotic liver disease.[57] Collectively, these findings suggest that DNA replication stress and DNA damage response may especially limit the regenerative capacity of the liver, which may have contributed to ALF in the four deceased subjects in this study. In line with this, a high increase in replicative stress was identified in the embryonic (and not adult) liver of a humanized mouse model of Seckel syndrome (MIM: PS210600), a form of MPD, with levels 2- to 3-fold higher in liver tissue than in brain or bone.[58]

Among the seven living subjects (group 3 in Table 1), two were homozygous for the c.1769−1G>C variant, while the remaining subjects were compound heterozygous, with combinations of a loss-of-function allele and an intronic or missense variant or two non-coding variants. The most common clinical features among this group were postnatal growth retardation, feeding difficulties, developmental delay, a small face with a bulbous nose and retrognathia, a high-pitched voice, and hip dislocation (Figure 1A; Table 1). The mildest MPD phenotype was observed in the 16-year-old S16, who carried the intronic variants c.2531+1G>A and c.1341+5G>T in *trans*. Altogether, our data establish *WDHD1* as a gene in which bi-allelic variants cause autosomal recessive MPD, with a phenotypic spectrum

overlapping that of *PRIM1*-, *DNA2*-, *DONSON*-, and *TRAIP*-related disorders.[32–36,38]

In primary fibroblasts or leukocytes from several subjects, we identified aberrant splicing of *WDHD1* pre-mRNAs due to the recurrent intronic variant c.1769−1G>C and the variants c.505−1G>A, c.1341+5_1341+6inv, c.1342−6T>A, and c.3190−1G>A. However, a small proportion of wild-type *WDHD1* transcripts was also detected in S13 cells. While total *WDHD1* mRNA levels were similar in subject- and control-derived fibroblasts, WDHD1 protein levels were markedly reduced in four subject cells. These data suggest that a residual amount of wild-type and/or variant WDHD1 protein is required to sustain limited cell proliferation and cell survival, as evidenced by the hypoproliferative phenotype and delayed cell cycle progression observed in fibroblasts with bi-allelic *WDHD1* variants. A similar delayed cell cycle and increased doubling time were detected in fibroblasts from subjects with bi-allelic variants in *PRIM1*, encoding the catalytic subunit of DNA primase.[38] These data led us to propose that the overall number of cells generated during development is likely profoundly compromised, leading to microcephaly, growth restriction, and other abnormalities in subjects with bi-allelic *WDHD1* variants. Accordingly, hypomorphic variants in *WDHD1* seem compatible with life, consistent with findings for *DONSON*, *PRIM1*, and other genes encoding replisome components.[28,32,34,38] The hypomorphic nature of the homozygous *WDHD1* nonsense variant identified in S10, potentially resulting from altered *WDHD1* pre-mRNA splicing, remains speculative. However, the requirement of Wdhd1 for early mouse embryogenesis[59] and *Drosophila* viability[60] further supports that partial loss of WDHD1 function is compatible with embryonic development, especially in higher eukaryotes.

Fibroblasts with bi-allelic *WDHD1* variants showed reduced DNA replication speed. Notably, the replication speed varied between subject cells, with S6 cells showing the highest speed and those from S13 the lowest. The most obvious explanation of slowed replication is the increased occurrence of endogenous DNA damage encountered by replication forks. *WDHD1*-knockdown cell lines show a comparable fork slowdown.[13,16] In addition, the number of stalled forks was significantly reduced in untreated subject-derived fibroblasts. This finding is in contrast with data from subject-derived fibroblasts with pathogenic variants in *PRIM1* and *GINS1*, which encode other replisome components, as well as *DONSON*-depleted HeLa cells, all of which showed increased spontaneous stalling of replication forks.[34,38,61] However, untreated subject cells with bi-allelic variants in *TRAIP*, encoding a replisome-associated E3 ubiquitin ligase, show no change in fork stalling.[33] These data suggest distinct roles of replisome components in the stabilization and/or restart of stalled replication forks. WDHD1's function in replication fork progression and protection[16] suggests that fibroblasts with bi-allelic *WDHD1* variants fail to efficiently regulate the replication. This may subsequently lead to the synthesis and accumulation of long ssDNA at replication forks, followed by the conversion into DNA double-strand breaks.[16,23,26] We tested this hypothesis by detecting phosphorylated RPA2, a marker of ssDNA formation (alternative name: RPA32),[62] in untreated and HU-treated subject and control fibroblasts and identified similar levels of phosphorylated RPA2 under both conditions (Figure S14). However, we found an increase in spontaneous γH2AX foci in fibroblasts with bi-allelic *WDHD1* variants, mirroring the phenotype of WDHD1-depleted eukaryotic cell lines.[15,16] The function of yeast Ctf4 in protecting arrested forks from breakage and end resection[23] further supports the idea that fibroblasts with bi-allelic *WDHD1* variants may be prone to genome rearrangements and genome instability.

When fibroblasts were treated with HU, subject cells had longer CldU-labeled replication tracks than control cells. Notably, we found an inverse correlation between CldU track length under HU treatment and replication fork speed under unperturbed conditions: the slower the replication speed, the longer the CldU-labeled track during replication stress. Accordingly, S13 fibroblasts, which showed the slowest replication speed, had the strongest increase in CldU-labeled tracks under HU treatment. One potential explanation is that the intracellular pool of deoxyribonucleotide triphosphates is relatively higher in these slow-replicating cells compared to cells with normal replication fork progression. HU treatment of fibroblasts with bi-allelic *WDHD1* variants increased the number of stalled forks, consistent with previous findings indicating that WDHD1 is required to maintain the stability of stalled replication forks following replication stress.[24] Thus, spontaneously elevated abundance of γH2AX foci and increased fork stalling after HU treatment suggest a compromised replication stress response in fibroblasts with bi-allelic *WDHD1* variants. Similarly, impaired DNA replication processes together with spontaneously accumulated DNA damages have been reported in subject-derived fibroblasts with bi-allelic variants in *ATR* (MIM: 601215), which encodes a serine-threonine kinase with a crucial role in the early steps of DNA damage response,[63] as well as in *ATRIP* (MIM: 606605), which encodes a binding partner of ATR.[64–66] Bi-allelic hypomorphic variants in *ATR* or *ATRIP* cause Seckel syndrome.[67,68] The mouse model of Seckel syndrome with a homozygous synonymous *Atr* variant recapitulates the human phenotype and demonstrates that reduced Atr function increases replicative stress and severely impairs embryonic development by activating an apoptotic DNA damage response.[58] This concept of intrauterine programming illustrates how increased replicative stress results in reduced body size and additional phenotypic abnormalities (see Figure 1 in O'Driscoll[69]), which may also apply to WDHD1 deficiency.

An increased nucleus size and altered nuclear morphology, including micro-, multilobed, and irregularly shaped nuclei, were observed in fibroblasts with bi-allelic *WDHD1* variants. In general, cells with abnormal nuclear envelopes are often characterized by an increase in DNA content, such as hyperploidy or polyploidy.[70,71] Thus, nuclear morphology changes are suggestive of DNA damage and genomic instability[70] and were also present in fibroblasts with bi-allelic variants in *GINS1*, which encodes a component of the replicative helicase CMG.[61] Together, the data suggest that defects in DNA replication, potentially resulting in DNA damage, are linked to compromised nuclear envelope integrity. In line with this, micronuclei are formed when cells proceed into mitosis following double-stranded DNA breaks.[72] Recent data showed that DNA damage can trigger nuclear envelope rupture by activation of the ATR kinase, which in turn promotes phosphorylation of lamin A/C. Phosphorylated lamin A/C alters lamina assembly, ultimately leading to nuclear envelope rupture, including micronuclei rupture, to eliminate damaged and aneuploid cells.[73,74] Failure to phosphorylate lamin A/C results in the formation of lamin A/C aggregates, including invaginations and foci, which colocalize with lamin B1.[74] Our observation of similar lamin B1-positive aberrant structures in fibroblasts with bi-allelic *WDHD1* variants may suggest that reduced or absent lamin A/C phosphorylation leads to abnormal nuclear envelope architecture. The increased number of micronuclei in subject-derived fibroblasts may also hint at inefficient ATR and CHK1 activation, possibly resulting in defective micronuclear rupture and clearance and, consequently, genomic instability.

The trend toward an increase in PCS in metaphases of fibroblasts with bi-allelic *WDHD1* variants provides the first evidence of a reduced capability of the cells to form the chromosomal cohesion complex that topologically encircles two duplicated sister DNAs until the onset of anaphase.[19] WDHD1 interacts with the cohesion components SMC1, SMC3, and RAD21/SCC1, and its yeast ortholog is required for cohesion between sister centromeres during yeast meiosis.[15,75] The function of yeast Ctf4 to recruit the helicase Chl1, a cohesion establishment factor, to the replication fork is essential for the establishment of sister chromatid cohesion.[20] Notably, Ctf4 promotes the loading of cohesin during ongoing DNA replication, enabling DNA-bound cohesin to subsequently capture and topologically entrap the two replicated sister chromatids.[76] Together, the data suggest that residual WDHD1 may be insufficient to maintain efficient sister chromatid cohesion in subject-derived fibroblasts.

In conclusion, we describe a distinct form of MPD caused by bi-allelic hypomorphic *WDHD1* variants that preserve residual variant and/or wild-type WDHD1 protein. The phenotypic spectrum ranges from early fetal lethality and ALF in the neonatal period to MPD without developmental delay in the mildest affected individual. Fibroblasts with bi-allelic *WDHD1* variants recapitulate almost all defects previously described in yeast Ctf4 mutants and *WDHD1*-depleted *Xenopus* egg extracts and human cell lines, such as impaired cell proliferation with a delay in G1-to-S transition, reduced replication speed, increased DNA damage and PCS, and abnormal nuclear morphology. This cellular phenotype is reminiscent of that of other DNA replication-associated genes implicated in developmental disorders with reduced organismal growth[28] and highlights the multifaceted roles of WDHD1 in maintaining genome integrity.

## Data and code availability

Data generated or analyzed during this study are included in the published article and the corresponding supplemental information. The raw sequencing data generated in the course of this study are not publicly available due to the protocol and the corresponding consents used, which did not include such information.

## Acknowledgments

We are grateful to the subjects and their families who agreed to participate in this project. We thank Lara Adrian, Sina Ramcke, and Jane Rehberg for skillful technical assistance; Jennifer Kaiser, Dung Ludwig, and Sigrid Fuchs for establishing primary skin fibroblast cultures and for assisting with and supervising the analysis of PCS; and the UKE Microscopy Imaging Facility (UMIF; Zeiss ApoTome microscope), and the Cytometry und Cell Sorting Core Facility at the University Medical Center Hamburg-Eppendorf for technical support. For subject 10, the Australian Undiagnosed Diseases Network (UDN-Aus) acknowledges financial support from the Australian Government's Medical Research Future Fund (MRFF; 2007567), Australian Genomics, and the Centre for Population Genomics (Garvan Institute of Medical Research and Murdoch Children's Research Institute), funded in part by an MRFF Genomics Health Futures Mission grant (2008820). A.A.L.J. was supported by the São Paulo Research Foundation (FAPESP; 2022/10107-6) and the National Council for Scientific and Technological Development (CNPq; 303294/2020-5). H.P. was supported by the BMFTR (German Federal Ministry of Research, Technology and Space) through the German Center for Child and Adolescent Health (DZKJ, 01GL2406B) and EJP RD project GENOMIT (01GM1920A and 01GM2404A), cofounded by the European Union, and the BMFTR through grants to the German Network for Mitochondrial Disorders (mitoNET, 01GM1906B). M.R.V. was supported by the BMFTR (02NUK104). K.B. and K.K. were supported by the Deutsche Forschungsgemeinschaft (467414153 to K.B. and KU 1240/16-1 to K.K.). The graphical abstract was created in BioRender (https://BioRender.com/kur3l4k).

## Author contributions

D.T. and K.K. contributed to the study conception and design. D.T., M.R.V., T.H., and L.D.S. performed the laboratory experiments and analyzed the experimental data. M.S., C.B., F.D., L.A., A. Kuechler, E.L., E.-D.P., A. Sabbagh, E.E.P., M.C., Y.K.,

UDN-Aus, D.P., D. Bartholdi, O.M., E.S.-G., A.T., A.A.L.J., H.G., L.D., and L.L. recruited and clinically assessed subjects and/or performed sample collection. WES, WGS, bioinformatics, and/or data analysis were performed by F.K., A. Knaus, P.K., A. Stalke, S.v.H., B.A., B.R., A.M.B.-A., S.A., R.R., UDN-Aus, D. Braun, A.D., A.R.J., A.T., N.Z., M.I.A., and A.A.L.J. K.B., H.P., and K.K. provided supervision. D.T., M.R.V., K.B., and K.K. wrote the first draft of the manuscript. A.A.L.J., H.P., M.R.V., K.B., and K.K. acquired funding. All authors approved the manuscript.

## Declaration of interests

M.S. is an employee of Eurofins GmbH. A.M.B.-A. and S.A. are employees of CENTOGENE GmbH. A.D. is an employee of CeGaT GmbH.

## Declaration of generative AI and AI-assisted technologies in the writing process

During the preparation of this manuscript, the authors used ChatGPT (OpenAI) to assist with improving the grammar and clarity of some sentences. The authors subsequently reviewed and edited all content to ensure accuracy and take full responsibility for the final manuscript.

## Supplemental information

Supplemental information can be found online at https://doi.org/10.1016/j.ajhg.2026.03.010.

## Web resources

GenBank, https://www.ncbi.nlm.nih.gov/genbank/
OMIM, https://www.omim.org

Received: December 18, 2025
Accepted: March 16, 2026
Published: April 9, 2026

## References

1. Limas, J.C., and Cook, J.G. (2019). Preparation for DNA replication: the key to a successful S phase. FEBS Lett. *593*, 2853–2867. https://doi.org/10.1002/1873-3468.13619.
2. Yao, N.Y., and O'Donnell, M.E. (2021). The DNA Replication Machine: Structure and Dynamic Function. Subcell. Biochem. *96*, 233–258. https://doi.org/10.1007/978-3-030-58971-4_5.
3. Ilves, I., Petojevic, T., Pesavento, J.J., and Botchan, M.R. (2010). Activation of the MCM2-7 helicase by association with Cdc45 and GINS proteins. Mol. Cell *37*, 247–258. https://doi.org/10.1016/j.molcel.2009.12.030.
4. Moyer, S.E., Lewis, P.W., and Botchan, M.R. (2006). Isolation of the Cdc45/Mcm2-7/GINS (CMG) complex, a candidate for the eukaryotic DNA replication fork helicase. Proc. Natl. Acad. Sci. USA *103*, 10236–10241. https://doi.org/10.1073/pnas.0602400103.
5. Lujan, S.A., Williams, J.S., and Kunkel, T.A. (2016). DNA Polymerases Divide the Labor of Genome Replication. Trends Cell Biol. *26*, 640–654. https://doi.org/10.1016/j.tcb.2016.04.012.
6. Branzei, D., Bene, S., Gangwani, L., and Szakal, B. (2024). The multifaceted roles of the Ctf4 replisome hub in the maintenance of genome integrity. DNA Repair *142*, 103742. https://doi.org/10.1016/j.dnarep.2024.103742.
7. Guan, C., Li, J., Sun, D., Liu, Y., and Liang, H. (2017). The structure and polymerase-recognition mechanism of the crucial adaptor protein AND-1 in the human replisome. J. Biol. Chem. *292*, 9627–9636. https://doi.org/10.1074/jbc.M116.758524.
8. Im, J.S., Ki, S.H., Farina, A., Jung, D.S., Hurwitz, J., and Lee, J.K. (2009). Assembly of the Cdc45-Mcm2-7-GINS complex in human cells requires the Ctf4/And-1, RecQL4, and Mcm10 proteins. Proc. Natl. Acad. Sci. USA *106*, 15628–15632. https://doi.org/10.1073/pnas.0908039106.
9. Jones, M.L., Baris, Y., Taylor, M.R.G., and Yeeles, J.T.P. (2021). Structure of a human replisome shows the organisation and interactions of a DNA replication machine. EMBO J. *40*, e108819. https://doi.org/10.15252/embj.2021108819.
10. Kilkenny, M.L., Simon, A.C., Mainwaring, J., Wirthensohn, D., Holzer, S., and Pellegrini, L. (2017). The human CTF4-orthologue AND-1 interacts with DNA polymerase alpha/primase via its unique C-terminal HMG box. Open Biol. *7*, 170217. https://doi.org/10.1098/rsob.170217.
11. Zhu, W., Ukomadu, C., Jha, S., Senga, T., Dhar, S.K., Wohlschlegel, J.A., Nutt, L.K., Kornbluth, S., and Dutta, A. (2007). Mcm10 and And-1/CTF4 recruit DNA polymerase alpha to chromatin for initiation of DNA replication. Genes Dev. *21*, 2288–2299. https://doi.org/10.1101/gad.1585607.
12. Baris, Y., Taylor, M.R.G., Aria, V., and Yeeles, J.T.P. (2022). Fast and efficient DNA replication with purified human proteins. Nature *606*, 204–210. https://doi.org/10.1038/s41586-022-04759-1.
13. Bermudez, V.P., Farina, A., Tappin, I., and Hurwitz, J. (2010). Influence of the human cohesion establishment factor Ctf4/AND-1 on DNA replication. J. Biol. Chem. *285*, 9493–9505. https://doi.org/10.1074/jbc.M109.093609.
14. Chen, Y., Liu, H., Zhang, H., Sun, C., Hu, Z., Tian, Q., Peng, C., Jiang, P., Hua, H., Li, X., and Pei, H. (2017). And-1 coordinates with CtIP for efficient homologous recombination and DNA damage checkpoint maintenance. Nucleic Acids Res. *45*, 2516–2530. https://doi.org/10.1093/nar/gkw1212.
15. Yoshizawa-Sugata, N., and Masai, H. (2009). Roles of human AND-1 in chromosome transactions in S phase. J. Biol. Chem. *284*, 20718–20728. https://doi.org/10.1074/jbc.M806711200.
16. Abe, T., Kawasumi, R., Giannattasio, M., Dusi, S., Yoshimoto, Y., Miyata, K., Umemura, K., Hirota, K., and Branzei, D. (2018). AND-1 fork protection function prevents fork resection and is essential for proliferation. Nat. Commun. *9*, 3091. https://doi.org/10.1038/s41467-018-05586-7.
17. Errico, A., Cosentino, C., Rivera, T., Losada, A., Schwob, E., Hunt, T., and Costanzo, V. (2009). Tipin/Tim1/And1 protein complex promotes Pol alpha chromatin binding and sister chromatid cohesion. EMBO J. *28*, 3681–3692. https://doi.org/10.1038/emboj.2009.304.
18. Tanaka, H., Kubota, Y., Tsujimura, T., Kumano, M., Masai, H., and Takisawa, H. (2009). Replisome progression complex links DNA replication to sister chromatid cohesion in Xenopus egg extracts. Genes Cells *14*, 949–963. https://doi.org/10.1111/j.1365-2443.2009.01322.x.

19. Uhlmann, F. (2016). SMC complexes: from DNA to chromosomes. Nat. Rev. Mol. Cell Biol. *17*, 399–412. https://doi.org/10.1038/nrm.2016.30.
20. Samora, C.P., Saksouk, J., Goswami, P., Wade, B.O., Singleton, M.R., Bates, P.A., Lengronne, A., Costa, A., and Uhlmann, F. (2016). Ctf4 Links DNA Replication with Sister Chromatid Cohesion Establishment by Recruiting the Chl1 Helicase to the Replisome. Mol. Cell *63*, 371–384. https://doi.org/10.1016/j.molcel.2016.05.036.
21. Técher, H., Koundrioukoff, S., Nicolas, A., and Debatisse, M. (2017). The impact of replication stress on replication dynamics and DNA damage in vertebrate cells. Nat. Rev. Genet. *18*, 535–550. https://doi.org/10.1038/nrg.2017.46.
22. Yates, L.A., Zhang, X., and Burgers, P.M. (2025). DNA Damage and Replication Stress Checkpoints. Annu. Rev. Biochem. *94*, 195–221. https://doi.org/10.1146/annurev-biochem-072324-031915.
23. Sasaki, M., and Kobayashi, T. (2017). Ctf4 Prevents Genome Rearrangements by Suppressing DNA Double-Strand Break Formation and Its End Resection at Arrested Replication Forks. Mol. Cell *66*, 533–545.e5. https://doi.org/10.1016/j.molcel.2017.04.020.
24. Hao, J., de Renty, C., Li, Y., Xiao, H., Kemp, M.G., Han, Z., DePamphilis, M.L., and Zhu, W. (2015). And-1 coordinates with Claspin for efficient Chk1 activation in response to replication stress. EMBO J. *34*, 2096–2110. https://doi.org/10.15252/embj.201488016.
25. Boavida, A., Napolitano, L.M., Santos, D., Cortone, G., Jegadesan, N.K., Onesti, S., Branzei, D., and Pisani, F.M. (2024). FANCJ DNA helicase is recruited to the replisome by AND-1 to ensure genome stability. EMBO Rep. *25*, 876–901. https://doi.org/10.1038/s44319-023-00044-y.
26. Fumasoni, M., Zwicky, K., Vanoli, F., Lopes, M., and Branzei, D. (2015). Error-free DNA damage tolerance and sister chromatid proximity during DNA replication rely on the Polalpha/Primase/Ctf4 Complex. Mol. Cell *57*, 812–823. https://doi.org/10.1016/j.molcel.2014.12.038.
27. Wang, N., Xu, S., and Egli, D. (2023). Replication stress in mammalian embryo development, differentiation, and reprogramming. Trends Cell Biol. *33*, 872–886. https://doi.org/10.1016/j.tcb.2023.03.015.
28. Bellelli, R., and Boulton, S.J. (2021). Spotlight on the Replisome: Aetiology of DNA Replication-Associated Genetic Diseases. Trends Genet. *37*, 317–336. https://doi.org/10.1016/j.tig.2020.09.008.
29. Alkuraya, F.S. (2015). Primordial dwarfism: an update. Curr. Opin. Endocrinol. Diabetes Obes. *22*, 55–64. https://doi.org/10.1097/MED.0000000000000121.
30. Klingseisen, A., and Jackson, A.P. (2011). Mechanisms and pathways of growth failure in primordial dwarfism. Genes Dev. *25*, 2011–2024. https://doi.org/10.1101/gad.169037.
31. Nielsen-Dandoroff, E., Ruegg, M.S.G., and Bicknell, L.S. (2023). The expanding genetic and clinical landscape associated with Meier-Gorlin syndrome. Eur. J. Hum. Genet. *31*, 859–868. https://doi.org/10.1038/s41431-023-01359-z.
32. Evrony, G.D., Cordero, D.R., Shen, J., Partlow, J.N., Yu, T.W., Rodin, R.E., Hill, R.S., Coulter, M.E., Lam, A.T.N., Jayaraman, D., et al. (2017). Integrated genome and transcriptome sequencing identifies a noncoding mutation in the genome replication factor DONSON as the cause of microcephaly-micromelia syndrome. Genome Res. *27*, 1323–1335. https://doi.org/10.1101/gr.219899.116.
33. Harley, M.E., Murina, O., Leitch, A., Higgs, M.R., Bicknell, L.S., Yigit, G., Blackford, A.N., Zlatanou, A., Mackenzie, K.J., Reddy, K., et al. (2016). TRAIP promotes DNA damage response during genome replication and is mutated in primordial dwarfism. Nat. Genet. *48*, 36–43. https://doi.org/10.1038/ng.3451.
34. Reynolds, J.J., Bicknell, L.S., Carroll, P., Higgs, M.R., Shaheen, R., Murray, J.E., Papadopoulos, D.K., Leitch, A., Murina, O., Tarnauskaitė, Ž., et al. (2017). Mutations in DONSON disrupt replication fork stability and cause microcephalic dwarfism. Nat. Genet. *49*, 537–549. https://doi.org/10.1038/ng.3790.
35. Shaheen, R., Faqeih, E., Ansari, S., Abdel-Salam, G., Al-Hassnan, Z.N., Al-Shidi, T., Alomar, R., Sogaty, S., and Alkuraya, F.S. (2014). Genomic analysis of primordial dwarfism reveals novel disease genes. Genome Res. *24*, 291–299. https://doi.org/10.1101/gr.160572.113.
36. Tarnauskaitė, Ž., Bicknell, L.S., Marsh, J.A., Murray, J.E., Parry, D.A., Logan, C.V., Bober, M.B., de Silva, D.C., Duker, A.L., Sillence, D., et al. (2019). Biallelic variants in DNA2 cause microcephalic primordial dwarfism. Hum. Mutat. *40*, 1063–1070. https://doi.org/10.1002/humu.23776.
37. Yamaguchi, M., and Cotterill, S. (2023). Association of Mutations in Replicative DNA Polymerase Genes with Human Disease: Possible Application of Drosophila Models for Studies. Int. J. Mol. Sci. *24*, 8078. https://doi.org/10.3390/ijms24098078.
38. Parry, D.A., Tamayo-Orrego, L., Carroll, P., Marsh, J.A., Greene, P., Murina, O., Uggenti, C., Leitch, A., Scottish Genomes Partnership, and Káposzta, R., et al. (2020). PRIM1 deficiency causes a distinctive primordial dwarfism syndrome. Genes Dev. *34*, 1520–1533. https://doi.org/10.1101/gad.340190.120.
39. Nikkilä, J., Parplys, A.C., Pylkäs, K., Bose, M., Huo, Y., Borgmann, K., Rapakko, K., Nieminen, P., Xia, B., Pospiech, H., and Winqvist, R. (2013). Heterozygous mutations in PALB2 cause DNA replication and damage response defects. Nat. Commun. *4*, 2578. https://doi.org/10.1038/ncomms3578.
40. Parplys, A.C., Petermann, E., Petersen, C., Dikomey, E., and Borgmann, K. (2012). DNA damage by X-rays and their impact on replication processes. Radiother. Oncol. *102*, 466–471. https://doi.org/10.1016/j.radonc.2012.01.005.
41. Sobreira, N., Schiettecatte, F., Valle, D., and Hamosh, A. (2015). GeneMatcher: a matching tool for connecting investigators with an interest in the same gene. Hum. Mutat. *36*, 928–930. https://doi.org/10.1002/humu.22844.
42. Chen, S., Francioli, L.C., Goodrich, J.K., Collins, R.L., Kanai, M., Wang, Q., Alföldi, J., Watts, N.A., Vittal, C., Gauthier, L.D., et al. (2024). A genomic mutational constraint map using variation in 76,156 human genomes. Nature *625*, 92–100. https://doi.org/10.1038/s41586-023-06045-0.
43. Rentzsch, P., Schubach, M., Shendure, J., and Kircher, M. (2021). CADD-Splice-improving genome-wide variant effect prediction using deep learning-derived splice scores. Genome Med. *13*, 31. https://doi.org/10.1186/s13073-021-00835-9.
44. Zhang, M.Q. (1998). Statistical features of human exons and their flanking regions. Hum. Mol. Genet. *7*, 919–932. https://doi.org/10.1093/hmg/7.5.919.
45. Shapiro, M.B., and Senapathy, P. (1987). RNA splice junctions of different classes of eukaryotes: sequence statistics and functional implications in gene expression. Nucleic Acids Res. *15*, 7155–7174. https://doi.org/10.1093/nar/15.17.7155.

46. Reese, M.G., Eeckman, F.H., Kulp, D., and Haussler, D. (1997). Improved splice site detection in Genie. J. Comput. Biol. *4*, 311–323. https://doi.org/10.1089/cmb.1997.4.311.
47. Yeo, G., and Burge, C.B. (2004). Maximum entropy modeling of short sequence motifs with applications to RNA splicing signals. J. Comput. Biol. *11*, 377–394. https://doi.org/10.1089/1066527041410418.
48. Köhler, A., Schmidt-Zachmann, M.S., and Franke, W.W. (1997). AND-1, a natural chimeric DNA-binding protein, combines an HMG-box with regulatory WD-repeats. J. Cell Sci. *110*, 1051–1062. https://doi.org/10.1242/jcs.110.9.1051.
49. Bianchi, V., Pontis, E., and Reichard, P. (1986). Changes of deoxyribonucleoside triphosphate pools induced by hydroxyurea and their relation to DNA synthesis. J. Biol. Chem. *261*, 16037–16042.
50. Petermann, E., Orta, M.L., Issaeva, N., Schultz, N., and Helleday, T. (2010). Hydroxyurea-stalled replication forks become progressively inactivated and require two different RAD51-mediated pathways for restart and repair. Mol. Cell *37*, 492–502. https://doi.org/10.1016/j.molcel.2010.01.021.
51. Rogakou, E.P., Pilch, D.R., Orr, A.H., Ivanova, V.S., and Bonner, W.M. (1998). DNA double-stranded breaks induce histone H2AX phosphorylation on serine 139. J. Biol. Chem. *273*, 5858–5868. https://doi.org/10.1074/jbc.273.10.5858.
52. Xu, H., Boone, C., and Brown, G.W. (2007). Genetic dissection of parallel sister-chromatid cohesion pathways. Genetics *176*, 1417–1429. https://doi.org/10.1534/genetics.107.072876.
53. Kajii, T., and Ikeuchi, T. (2004). Premature chromatid separation (PCS) vs. premature centromere division (PCD). Am. J. Med. Genet. *126A*, 433–434. https://doi.org/10.1002/ajmg.a.20612.
54. Viswanathan, P., Sharma, Y., Gupta, P., and Gupta, S. (2018). Replicative stress and alterations in cell cycle checkpoint controls following acetaminophen hepatotoxicity restrict liver regeneration. Cell Prolif. *51*, e12445. https://doi.org/10.1111/cpr.12445.
55. Wilson, S.R., and Duncan, A.W. (2023). The Ploidy State as a Determinant of Hepatocyte Proliferation. Semin. Liver Dis. *43*, 460–471. https://doi.org/10.1055/a-2211-2144.
56. Gentric, G., Maillet, V., Paradis, V., Couton, D., L'Hermitte, A., Panasyuk, G., Fromenty, B., Celton-Morizur, S., and Desdouets, C. (2015). Oxidative stress promotes pathologic polyploidization in nonalcoholic fatty liver disease. J. Clin. Investig. *125*, 981–992. https://doi.org/10.1172/JCI73957.
57. Donne, R., Saroul-Ainama, M., Cordier, P., Hammoutene, A., Kabore, C., Stadler, M., Nemazanyy, I., Galy-Fauroux, I., Herrag, M., Riedl, T., et al. (2022). Replication stress triggered by nucleotide pool imbalance drives DNA damage and cGAS-STING pathway activation in NAFLD. Dev. Cell *57*, 1728–1741.e6. https://doi.org/10.1016/j.devcel.2022.06.003.
58. Murga, M., Bunting, S., Montaña, M.F., Soria, R., Mulero, F., Cañamero, M., Lee, Y., McKinnon, P.J., Nussenzweig, A., and Fernandez-Capetillo, O. (2009). A mouse model of ATR-Seckel shows embryonic replicative stress and accelerated aging. Nat. Genet. *41*, 891–898. https://doi.org/10.1038/ng.420.
59. Fan, H.H., Lee, K.H., Chen, Y.T., Lin, L.J., Yang, T.L., Lin, S.W., and Yu, I.S. (2021). Wdhd1 is essential for early mouse embryogenesis. Biochim. Biophys. Acta. Mol. Cell Res. *1868*, 119011. https://doi.org/10.1016/j.bbamcr.2021.119011.
60. Gosnell, J.A., and Christensen, T.W. (2011). Drosophila Ctf4 is essential for efficient DNA replication and normal cell cycle progression. BMC Mol. Biol. *12*, 13. https://doi.org/10.1186/1471-2199-12-13.
61. Cottineau, J., Kottemann, M.C., Lach, F.P., Kang, Y.H., Vély, F., Deenick, E.K., Lazarov, T., Gineau, L., Wang, Y., Farina, A., et al. (2017). Inherited GINS1 deficiency underlies growth retardation along with neutropenia and NK cell deficiency. J. Clin. Investig. *127*, 1991–2006. https://doi.org/10.1172/JCI90727.
62. Choi, J.H., Lindsey-Boltz, L.A., Kemp, M., Mason, A.C., Wold, M.S., and Sancar, A. (2010). Reconstitution of RPA-covered single-stranded DNA-activated ATR-Chk1 signaling. Proc. Natl. Acad. Sci. USA *107*, 13660–13665. https://doi.org/10.1073/pnas.1007856107.
63. Blackford, A.N., and Jackson, S.P. (2017). ATM, ATR, and DNA-PK: The Trinity at the Heart of the DNA Damage Response. Mol. Cell *66*, 801–817. https://doi.org/10.1016/j.molcel.2017.05.015.
64. Cortez, D., Guntuku, S., Qin, J., and Elledge, S.J. (2001). ATR and ATRIP: partners in checkpoint signaling. Science *294*, 1713–1716. https://doi.org/10.1126/science.1065521.
65. Duthoo, E., Beyls, E., Backers, L., Gudjónsson, T., Huang, P., Jonckheere, L., Riemann, S., Parton, B., Du, L., Debacker, V., et al. (2025). Replication stress, microcephalic primordial dwarfism, and compromised immunity in ATRIP deficient patients. J. Exp. Med. *222*, e20241432. https://doi.org/10.1084/jem.20241432.
66. Mokrani-Benhelli, H., Gaillard, L., Biasutto, P., Le Guen, T., Touzot, F., Vasquez, N., Komatsu, J., Conseiller, E., Pïcard, C., Gluckman, E., et al. (2013). Primary microcephaly, impaired DNA replication, and genomic instability caused by compound heterozygous ATR mutations. Hum. Mutat. *34*, 374–384. https://doi.org/10.1002/humu.22245.
67. O'Driscoll, M., Ruiz-Perez, V.L., Woods, C.G., Jeggo, P.A., and Goodship, J.A. (2003). A splicing mutation affecting expression of ataxia-telangiectasia and Rad3-related protein (ATR) results in Seckel syndrome. Nat. Genet. *33*, 497–501. https://doi.org/10.1038/ng1129.
68. Ogi, T., Walker, S., Stiff, T., Hobson, E., Limsirichaikul, S., Carpenter, G., Prescott, K., Suri, M., Byrd, P.J., Matsuse, M., et al. (2012). Identification of the first ATRIP-deficient patient and novel mutations in ATR define a clinical spectrum for ATR-ATRIP Seckel Syndrome. PLoS Genet. *8*, e1002945. https://doi.org/10.1371/journal.pgen.1002945.
69. O'Driscoll, M. (2009). Life can be stressful without ATR. Nat. Genet. *41*, 866–868. https://doi.org/10.1038/ng0809-866.
70. Gauthier, B.R., and Comaills, V. (2021). Nuclear Envelope Integrity in Health and Disease: Consequences on Genome Instability and Inflammation. Int. J. Mol. Sci. *22*, 7281. https://doi.org/10.3390/ijms22147281.
71. Maciejowski, J., and Hatch, E.M. (2020). Nuclear Membrane Rupture and Its Consequences. Annu. Rev. Cell Dev. Biol. *36*, 85–114. https://doi.org/10.1146/annurev-cellbio-020520-120627.
72. Harding, S.M., Benci, J.L., Irianto, J., Discher, D.E., Minn, A.J., and Greenberg, R.A. (2017). Mitotic progression following DNA damage enables pattern recognition within micronuclei. Nature *548*, 466–470. https://doi.org/10.1038/nature23470.
73. Joo, Y.K., Black, E.M., Trier, I., Haakma, W., Zou, L., and Kabeche, L. (2023). ATR promotes clearance of damaged DNA and damaged cells by rupturing micronuclei. Mol. Cell *83*,

3642–3658.e4. https://doi.org/10.1016/j.molcel.2023.09.003.

74. Kovacs, M.T., Vallette, M., Wiertsema, P., Dingli, F., Loew, D., Nader, G.P.d.F., Piel, M., and Ceccaldi, R. (2023). DNA damage induces nuclear envelope rupture through ATR-mediated phosphorylation of lamin A/C. Mol. Cell *83*, 3659–3668.e10. https://doi.org/10.1016/j.molcel.2023.09.023.

75. Petronczki, M., Chwalla, B., Siomos, M.F., Yokobayashi, S., Helmhart, W., Deutschbauer, A.M., Davis, R.W., Watanabe, Y., and Nasmyth, K. (2004). Sister-chromatid cohesion mediated by the alternative RF-CCtf18/Dcc1/Ctf8, the helicase Chl1 and the polymerase-alpha-associated protein Ctf4 is essential for chromatid disjunction during meiosis II. J. Cell Sci. *117*, 3547–3559. https://doi.org/10.1242/jcs.01231.

76. Murayama, Y., Endo, S., Kurokawa, Y., Kurita, A., Iwasaki, S., and Araki, H. (2024). Coordination of cohesin and DNA replication observed with purified proteins. Nature *626*, 653–660. https://doi.org/10.1038/s41586-023-07003-6.

The American Journal of Human Genetics, Volume *113*

## Supplemental information

# Bi-allelic *WDHD1* variants

# cause microcephalic primordial dwarfism

**Debora Tibbe, Marie Ronja Vogt, Tess Holling, Lea Dewi Schlieben, Fanny Kortüm, Moneef Shoukier, Christoph Bagowski, Felix Distelmaier, Luisa Averdunk, Alexej Knaus, Peter Krawitz, Alma Kuechler, Elke Lainka, Amelie Stalke, Sandra von Hardenberg, Bernd Auber, Eva-Doreen Pfister, Bruno Reversade, Anthony Sabbagh, Aida M. Bertoli-Avella, Salem Alawbathani, Elizabeth E. Palmer, Manisha Chauhan, Rocio Rius, Yoonji Kim, Australian Undiagnosed Diseases Network (UDN-Aus), Dzhoy Papingi, Deborah Bartholdi, Dominique Braun, Oliver Maier, April Dinwiddie, Elisabeth Steichen-Gersdorf, Andreas R. Janecke, Anatoly Tiulpakov, Nikolay Zernov, Maria Izabel Arismendi, Alexander A.L. Jorge, Himanshu Goel, Lauren Dreyer, Lily Loughman, Holger Prokisch, Kerstin Borgmann, and Kerstin Kutsche**

## Supplemental Information

### Supplemental figures and legends

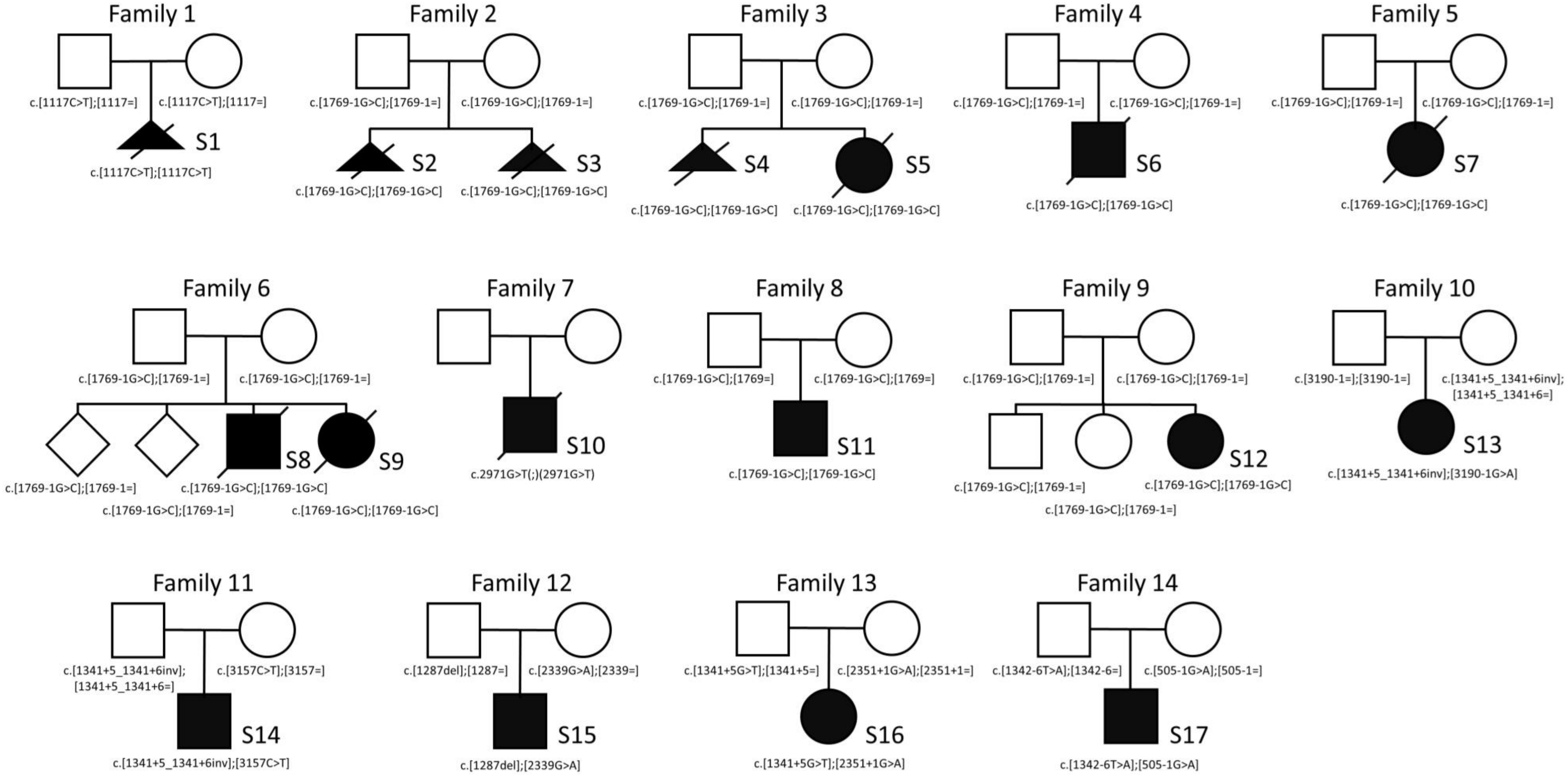


**Figure S1.** Pedigrees of 17 subjects with biallelic *WDHD1* variants.

Males are represented by squares and females by circles. Triangles indicate pregnancies not carried to term. Filled symbols denote individuals affected by the disease, and a diagonal slash indicates a deceased individual. The genotype, if known, is shown below each symbol.

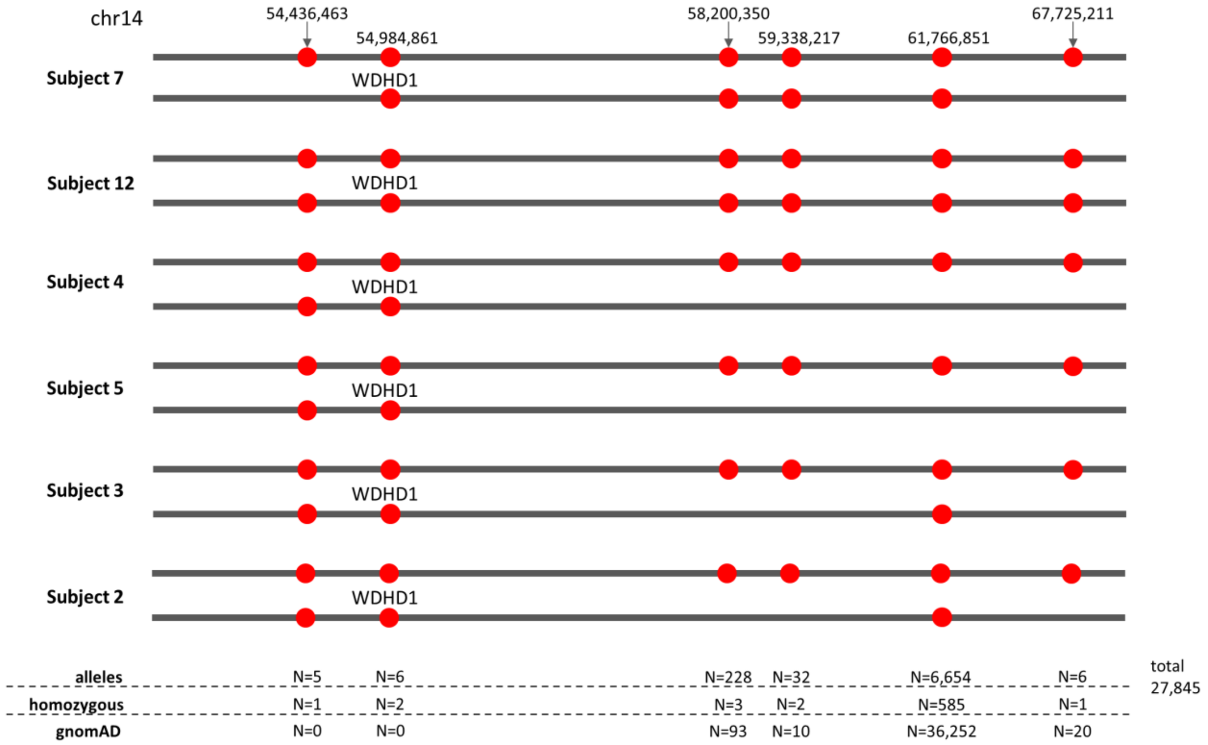


**Figure S2.** A specific haplotype is shared among subjects with the *WDHD1* variant c.1769-1G>C.

Analysis of the haplotype based on genotypes of rare single nucleotide variants on chromosome 14 in an in-house database with 27,845 WES cases and in gnomAD for subjects 2-5, 7, and 12 carrying the homozygous splice site variant c.1769-1G>C in *WDHD1*. Schematic visualization of selected rare variants (red dots) on chromosome 14 (grey line) in proximity to *WDHD1* and the shared homozygous *WDHD1* variant c.1769-1G>C. Chromosome positions are indicated according to GRCh38. Allele count and number of homozygous individuals in the in-house database as well as the allele frequency in the gnomAD database (v2.1.1) are given below the haplotypes.

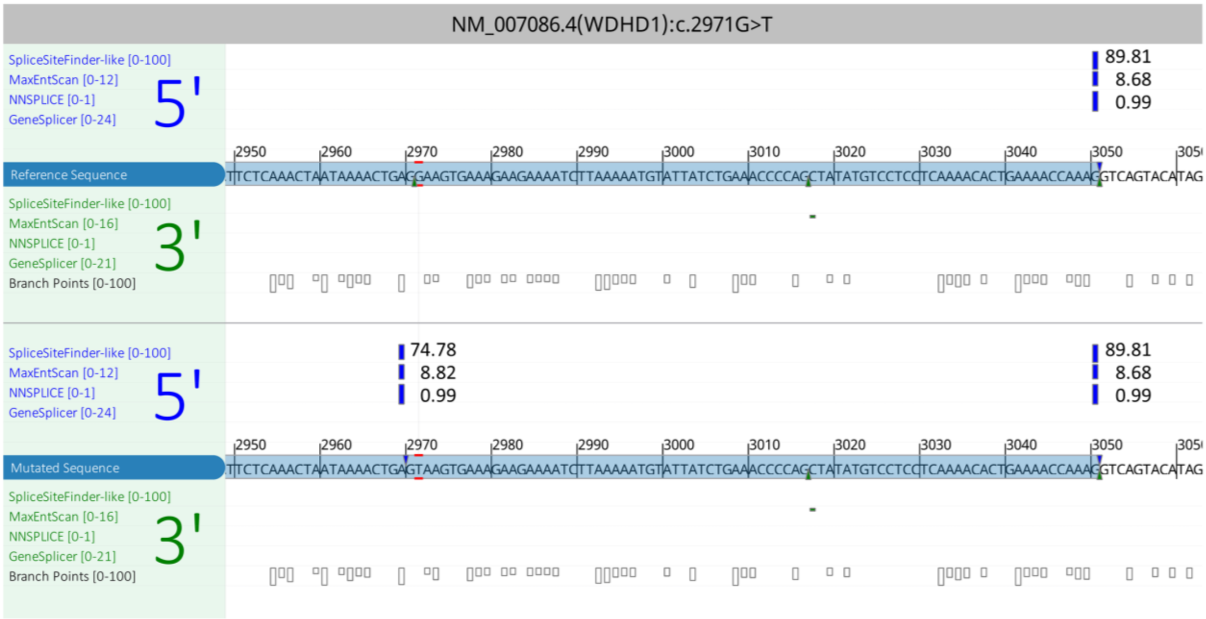


**Figure S3**. Splice site predictions for the homozygous *WDHD1* nonsense variant c.2971G>T identified in subject 10.

Screenshots from the splicing module of Alamut Visual Plus (v1.4; SOPHIA GENETICS) show partial sequences of the exon 24-intron 24 boundary of *WDHD1* (NM_007086.4). The reference sequence is displayed in the upper panel and the sequence with the variant c.2971G>T in the lower panel. The exonic sequence is highlighted in blue. Splice sites were predicted using the programs SpliceSiteFinder-like, NNSPLICE 0.9 version, MaxEntScan, and GeneSplicer. Predicted splice donor sites are marked by blue rectangles within the sequence. Corresponding scores are provided (see Table 2) and are displayed on the right of the blue boxes shown above the sequence.

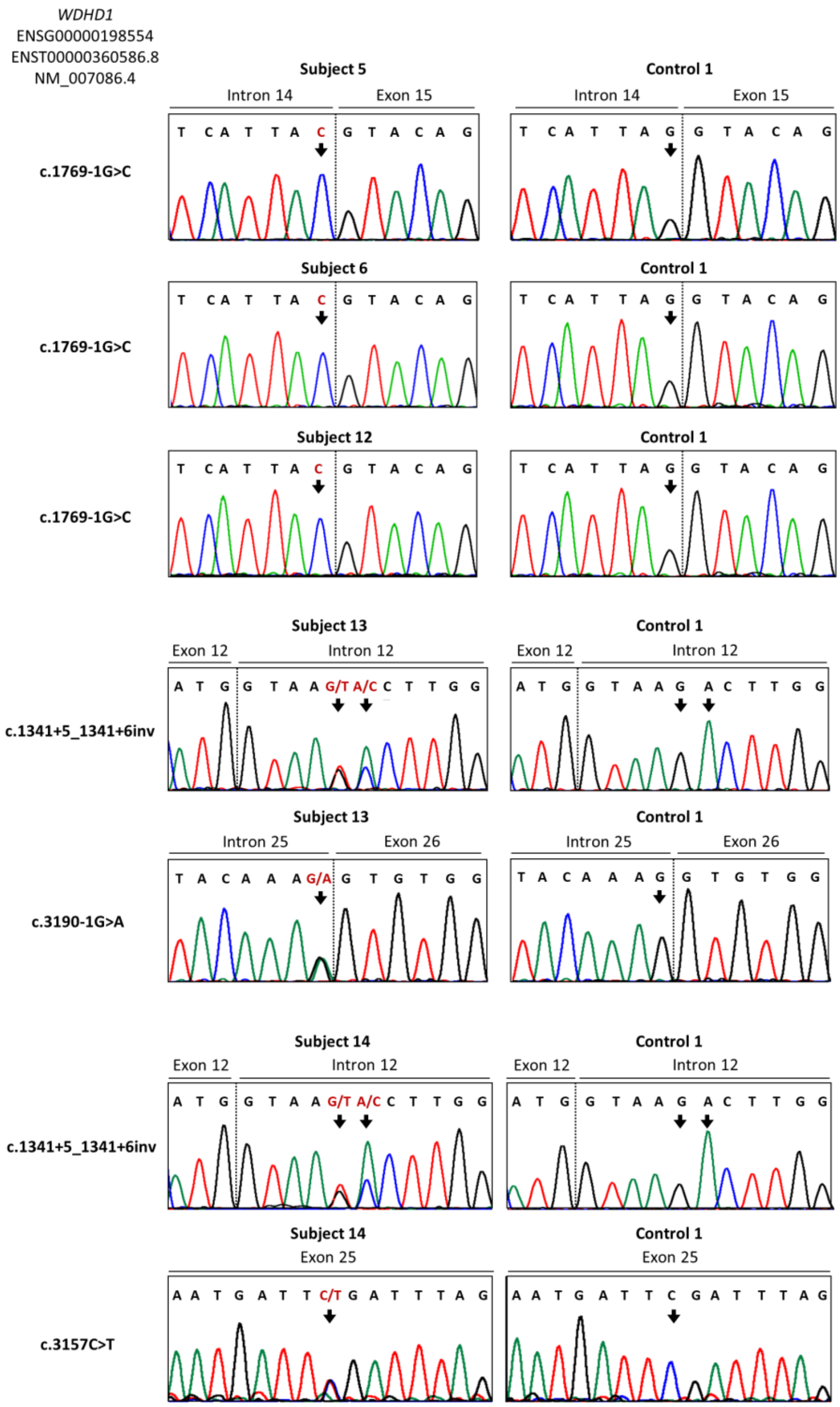


**Figure S4.** Validation of biallelic *WDHD1* variants in subject-derived fibroblasts.

Partial sequence electropherograms show the *WDHD1* splice site variant c.1769-1G>C in the homozygous state in DNA of fibroblasts derived from subjects 5, 6, and 12, compared to the reference sequence (control 1). Sanger sequencing confirmed the presence of the heterozygous variant c.1341+5_1241+6inv in fibroblasts of subjects 13 and 14, the heterozygous variant c.3190-1G>A in subject 13 cells, and the heterozygous variant c.3157C>T in subject 14 cells. The respective reference sequence is shown on the right (control 1). Arrows indicate the positions of the variants (highlighted in red) in the sequence derived from subject fibroblasts, as well as the corresponding positions in the reference sequence (control 1). Exon-intron boundaries are marked with dotted lines.

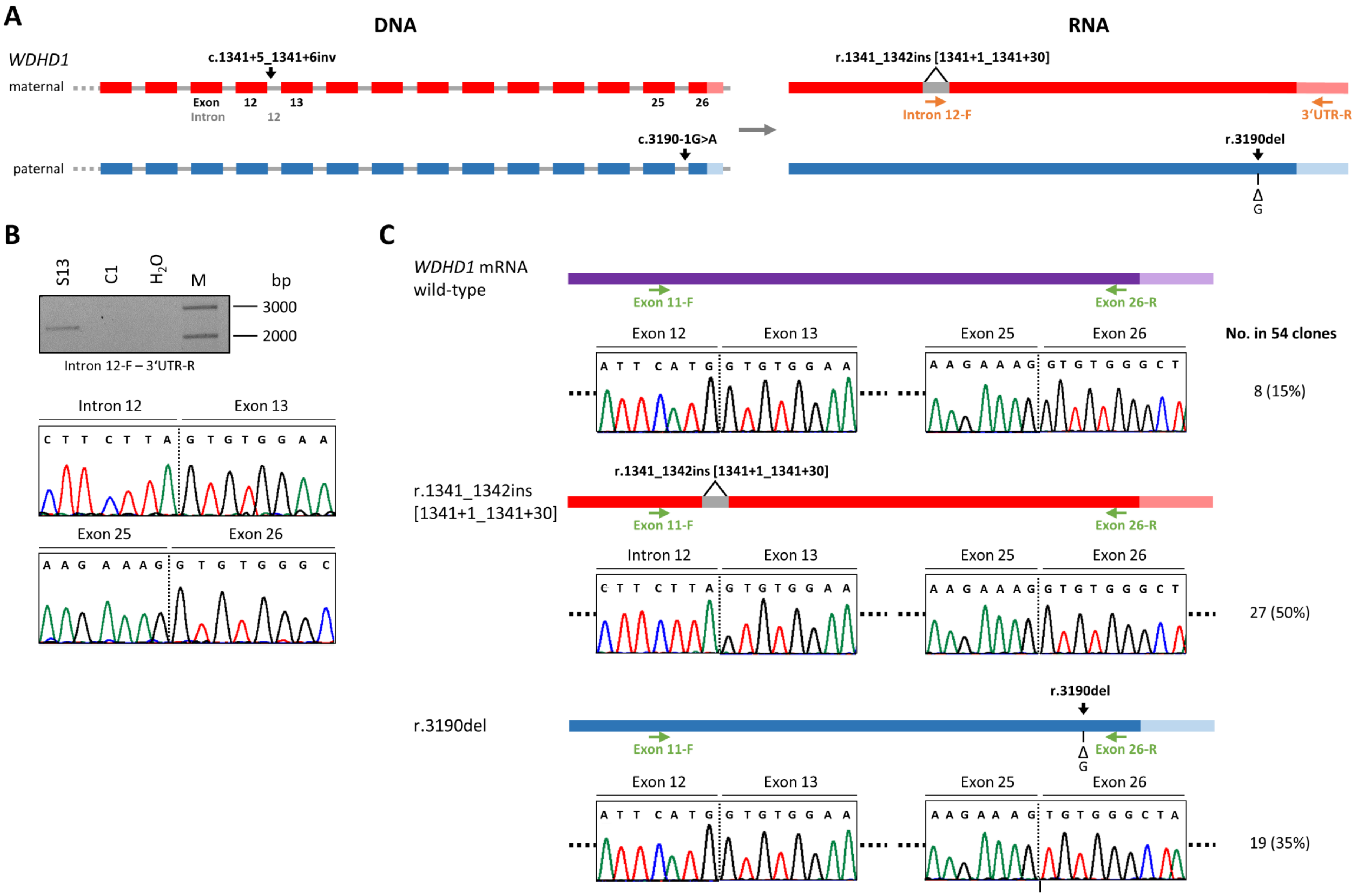
A
DNA
RNA
WDHD1
maternal
paternal
c.1341+5_1341+6inv
Exon 12 13
Intron 12
25 26
c.3190-1G>A
r.1341_1342ins [1341+1_1341+30]
Intron 12-F
3'UTR-R
r.3190del
Δ G
B
S13
C1
H2O
M
bp
3000
2000
Intron 12-F – 3'UTR-R
Intron 12
Exon 13
C T T C T T A G T G T G G A A
Exon 25
Exon 26
A A G A A A G G T G T G G G C
C
WDHD1 mRNA wild-type
Exon 11-F
Exon 26-R
Exon 12
Exon 13
Exon 25
Exon 26
No. in 54 clones
A T T C A T G G T G T G G A A
A A G A A A G G T G T G G G C T
8 (15%)
r.1341_1342ins [1341+1_1341+30]
Intron 12
C T T C T T A G T G T G G A A
27 (50%)
r.3190del
A A G A A A G T G T G G G C T A
19 (35%)

**Figure S5.** Determination of the allelic phase of the two *WDHD1* variants in subject 13.

(A) Schematic representation of the partial *WDHD1* gene (DNA, left) and *WDHD1* mRNA (right) derived from the maternal (red) and paternal (blue) alleles. Coding regions are shown as red or blue boxes and intronic regions as grey lines. 3' untranslated regions (3'UTRs) are shown in light red or light blue. Arrows indicate the positions of the variants. Black arrows and lines indicate the results of aberrant splicing events on the *WDHD1* transcripts (right). The localization of the primers used for specific amplification of a *WDHD1* transcript with the 30 bp intronic insertion is shown in orange. ΔG, deletion of a guanine. (B) Agarose gel showing an RT-PCR amplicon (2286 bp) specifically generated from leukocyte-derived RNA (cDNA) of subject 13 (S13). The used primers are shown in (A, right), spanning the first 30 bp of intron 12 to the 3'UTR. Sanger sequencing of this RT-PCR product revealed the intron 12-exon 13 junction together with canonically spliced exons 25 and 26. (C) Schematic representation of *WDHD1* transcript structure and RT-PCR strategy used to assess the allelic phase of the c.1341+5_1341+6inv and the c.3190-1G>A variants. The positions of aberrant splicing effects (black) and the primer pair used (green) are indicated. RT-PCR products amplified with primers spanning exon 11 to exon 26 were cloned, and inserts from individual clones were subsequently Sanger sequenced. Representative Sanger traces show the exon 12-exon 13 and exon 25-exon 26 junctions within the same representative cloned insert. The proportions of the different *WDHD1* transcripts, including the wild-type (purple), transcripts with r.1341_1342ins[1341+1_1341+30] (red), and transcripts with r.3190del (blue), among the 54 analyzed clones are shown as percentages to the right of the Sanger sequencing traces.

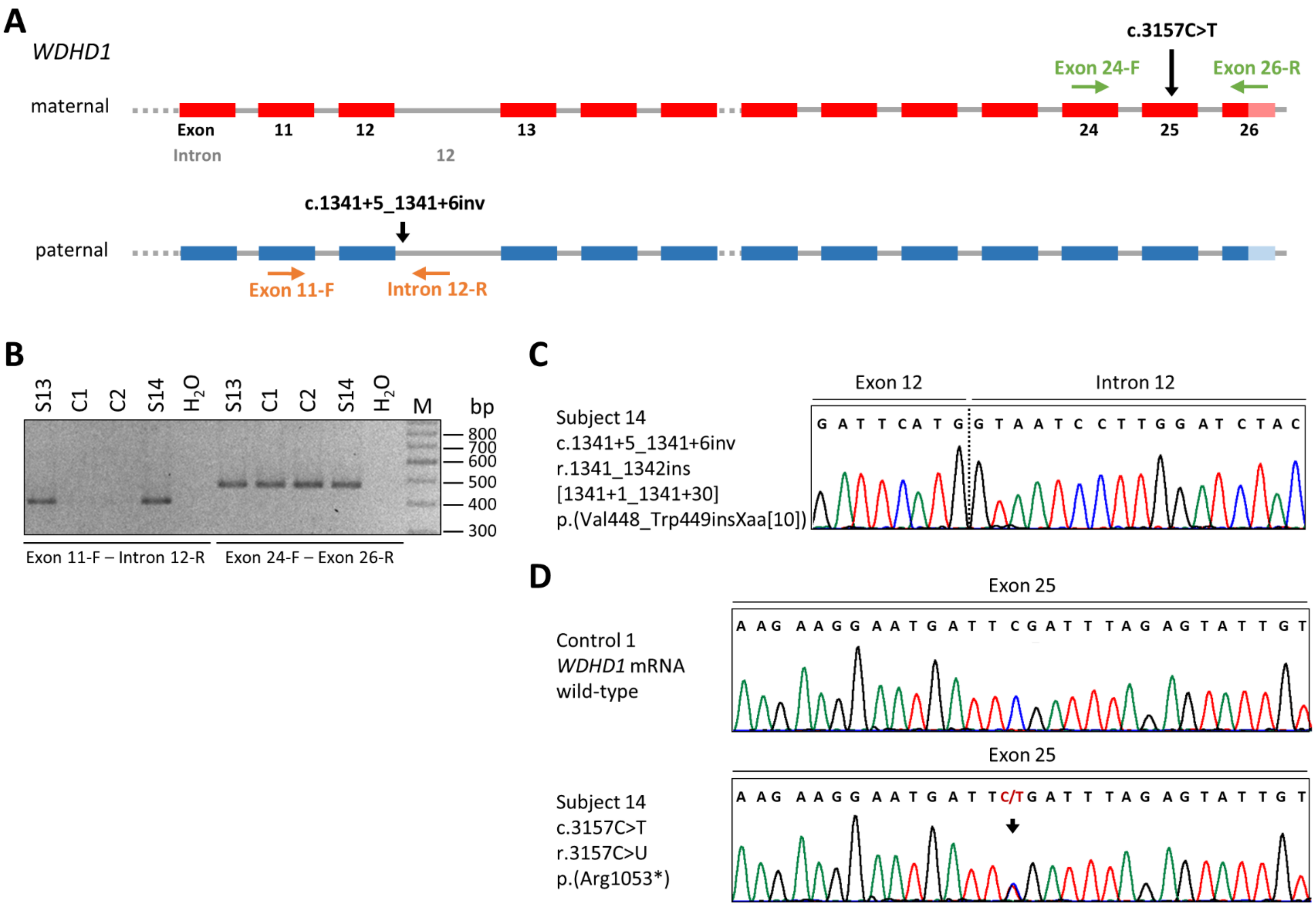
A
WDHD1
maternal
c.3157C>T
Exon 24-F
Exon 26-R
Exon
11
12
13
24
25
26
Intron
12
paternal
c.1341+5_1341+6inv
Exon 11-F
Intron 12-R
B
S13
C1
C2
S14
$H_2O$
M
bp
800
700
600
500
400
300
Exon 11-F – Intron 12-R
Exon 24-F – Exon 26-R
C
Exon 12
Intron 12
Subject 14
c.1341+5_1341+6inv
r.1341_1342ins
[1341+1_1341+30]
p.(Val448_Trp449insXaa[10])
G A T T C A T G G T A A T C C T T G G A T C T A C
D
Exon 25
Control 1
WDHD1 mRNA
wild-type
A A G A A G G A A T G A T T C G A T T T A G A G T A T T G T
Subject 14
c.3157C>T
r.3157C>U
p.(Arg1053*)
A A G A A G G A A T G A T T C/T G A T T T A G A G T A T T G T

**Figure S6.** Analysis of *WDHD1* transcripts in fibroblasts of subject 14 harboring the variants c.1341+5_1341+6inv and c.3157C>T.

(A) Schematic representation of partial *WDHD1* gene and the RT-PCR primers used to detect the insertion of the first 30 bp of intron 12 and to assess the effect of the nonsense variant c.3157C>T. Coding regions are shown as red or blue boxes, intronic regions as grey lines, and 3' untranslated regions as light red and light blue boxes. The positions of primers used for amplification of the exon 24-exon 26 RT-PCR product (green) and the PCR product containing the intron 12 insertion (orange) are indicated. Black arrows mark the positions of the variants. (B) Agarose gel showing RT-PCR amplicons generated from fibroblast-derived RNA (cDNA) of fibroblasts from subjects 13 and 14 (S13, S14) and two controls (C1, C2). Primers spanning exon 11 to intron 12 produced the expected 406-bp product in subject 13 and 14 cells but not in control cells. The primer combination spanning exon 24 to exon 26 yielded a PCR product of 470 bp in subject and control cells. (C) Partial Sanger trace of the exon 11-intron 12 RT-PCR product obtained from subject 14 cells (shown in B). Sequencing confirmed splicing of exon 12 to the first 30 bp of intron 12 in subject 14 cells. (D) Partial Sanger traces of the exon 24-exon 26 RT-PCR product obtained from control 1 and subject 14 cells (shown in B). Sequencing of the 470-bp amplicons revealed wild-type *WDHD1* transcripts in control 1 cells, whereas fibroblasts from subject 14 expressed both the wild-type *WDHD1* transcript (r.3157C) and the variant transcript (r.3157U; indicated by a black arrow) in approximately equal amounts.

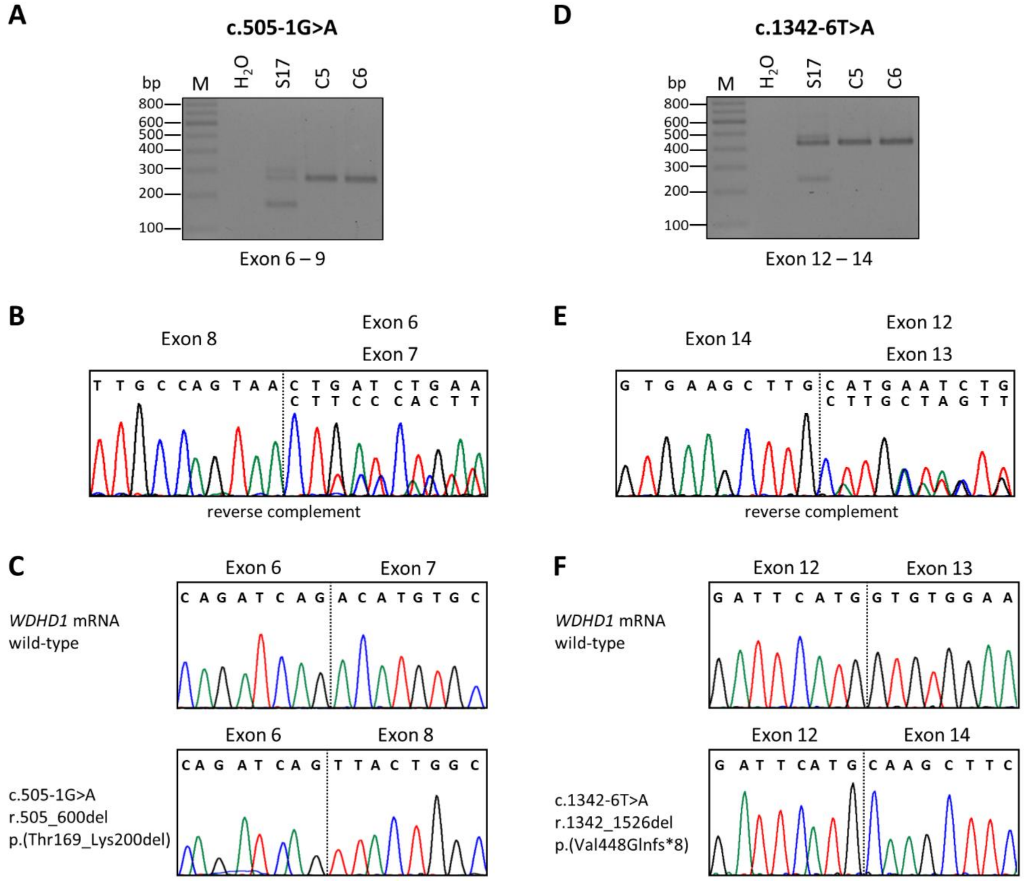


**Figure S7:** Analysis of *WDHD1* transcripts in leukocytes of S17 harboring the variants c.505-1G>A and c.1342-6T>A.

(A) Agarose gel showing RT-PCR amplicons generated from leukocyte-derived RNA (cDNA) of S17 with the heterozygous c.505-1G>A variant and two controls (C5, C6) using a primer pair targeting exons 6-9 (expected size: 264 bp). (B) Partial sequence electropherogram (reverse complement sequence shown) of directly sequenced RT-PCR products from S17 amplified with exon 6-9 primer pair. The reverse complement sequence spans the exon 8-7 junction and shows two overlapping sequences corresponding to exon 6 (upper sequence) and exon 7 (lower sequence). (C) Partial sequence electropherograms of cloned RT-PCR products. The upper panel shows the exon 6-7 junction of the *WDHD1* wild-type transcript, whereas the lower panel demonstrates skipping of exon 7 in *WDHD1* mRNAs of S17's leukocytes [r.505_600del; p.(Thr169_Lys200del)]. (D) Agarose gel showing RT-PCR amplicons generated from leukocyte-derived RNA (cDNA) of S17 with the heterozygous c.1342-6T>A variant and two controls (C5, C6) using a primer pair targeting exons 12-14 (expected size: 427 bp). The amplicons that appear slightly larger than the wild-type bands in S17 (see A and D) most likely represent heteroduplexes formed by two non-complementary strands, such as one strand of the wild-type (larger) amplicon and one strand of the smaller amplicon. (E) Partial sequence electropherogram (reverse complement sequence shown) of directly sequenced RT-PCR products from S17 amplified with exon 12-14 primer pair. The reverse complement sequence spans the exon 14-13 junction and shows two overlapping sequences corresponding to exon 12 (upper sequence) and exon 13 (lower sequence). (F) Partial sequence electropherograms of cloned RT-PCR products. The upper panel shows the exon 12-13 junction of the *WDHD1* wild-type transcript, whereas the lower panel demonstrates skipping of exon 13 in *WDHD1* mRNAs of S17's leukocytes [r.1342_1526del; p.(Val448Glnfs*8)].

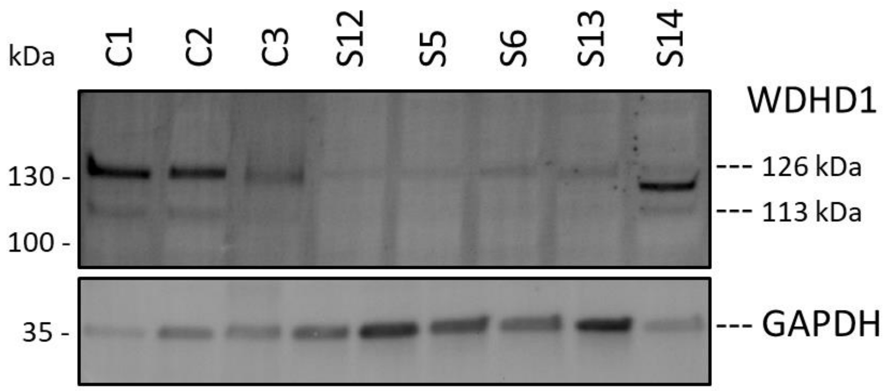


**Figure S8.** Long-exposure immunoblot showing WDHD1 protein levels in control- and subject-derived fibroblasts.

Representative immunoblot of fibroblast lysates from S12, S5, S6, S13, and S14 and three controls is shown. The blot was exposed for a longer duration to enhance visualization of faint bands. Levels of endogenous WDHD1 isoforms of 126 kDa and 113 kDa, as well as the C-terminally truncated proteoform in S14 cells, were assessed using the indicated antibodies, with GAPDH serving as a loading control.

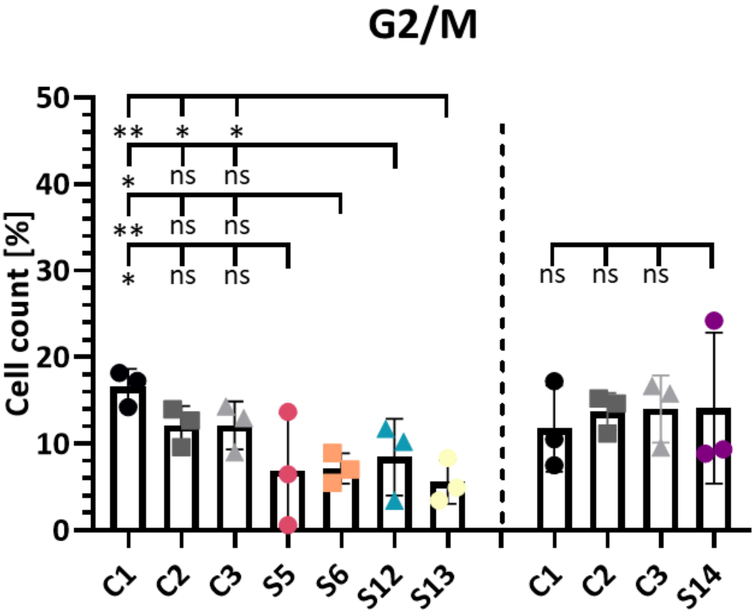


**Figure S9.** Quantification of control and subject cells in the G2/M phase.

Quantification of control and subject cells in the G2/M phase, derived from data shown in figure 3C. The percentage of cells in the G2/M phase is shown as the mean ± SD of three independent experiments. S14 fibroblasts were analyzed in a separate experiment with their own set of control cells. Statistical significance was determined by one-way ANOVA followed by Dunnett's multiple comparisons test. C1–C3, control fibroblasts; ns, not significant; S5, S6, S12-S14, subject fibroblasts. *$P \leq 0.05$; **$P \leq 0.01$.

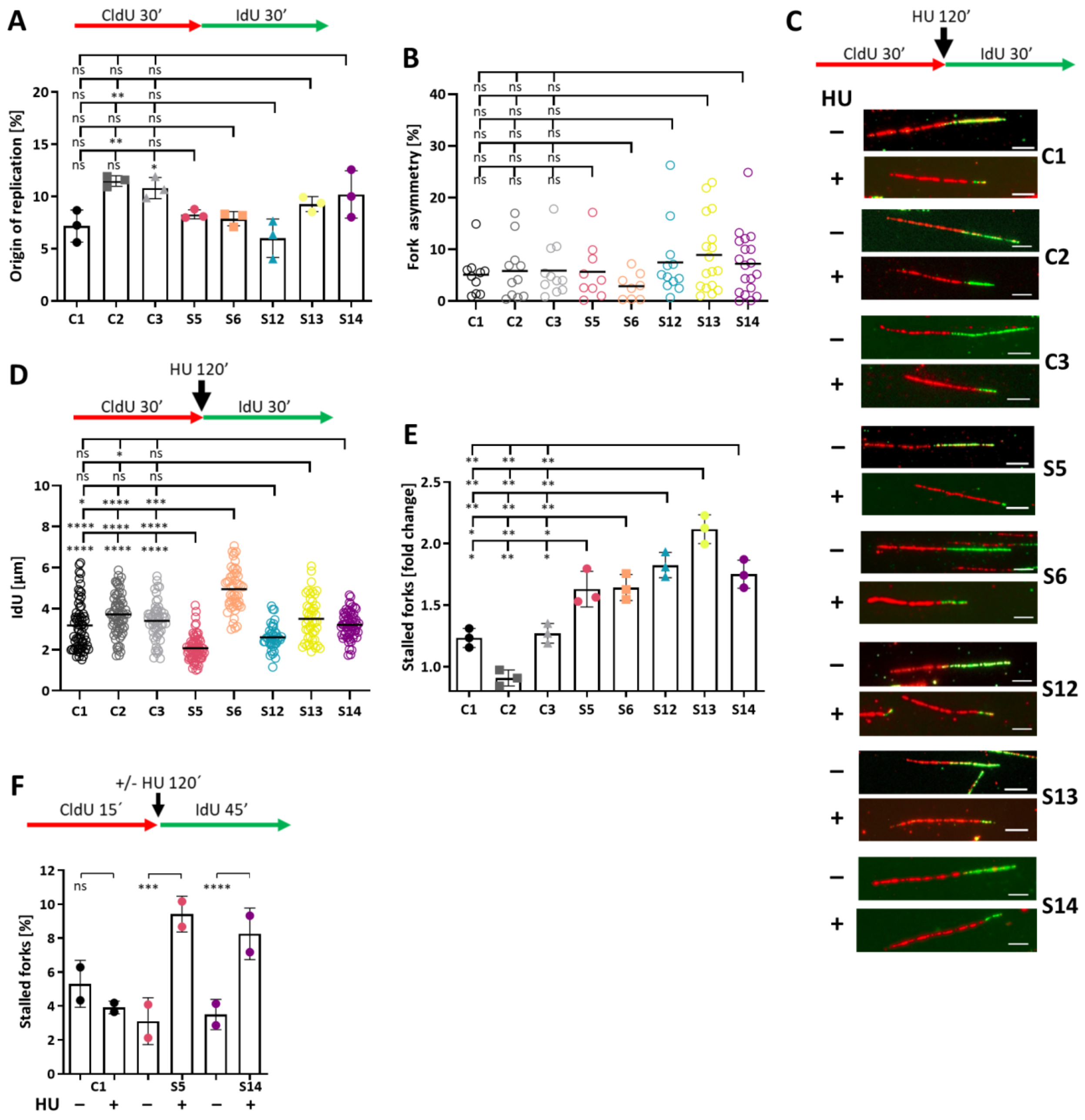


**Figure S10.** Analysis of origin of replication (ORI) and fork asymmetry under untreated conditions and of CldU length and stalled forks under hydroxyurea (HU)-treated conditions in subject and control fibroblasts.

(A) Treatment scheme of the DNA fiber assay with CldU and IdU labelling of cells for 30 minutes each, followed by immunofluorescence detection (CldU – red, IdU – green, top). Quantification of ORI in fibroblasts from S5, S6, and S12-S14, and three controls (bottom). The proportions of ORI were quantified relative to actively replicating forks and are presented as percentages. A minimum of 50 structures per experiment were analyzed for each cell line. The mean ± SD of three independent experiments is shown. (B) Analysis of replication fork symmetry was performed in fibroblasts from S5, S6, S12-S14, and three controls. Lengths of the bidirectional second tracks originating from an ORI were measured. The difference in length between each pair of second tracks was normalized to the total fiber length. In fibroblasts from C1-C3, the mean difference was 5.5% and determined as threshold for fork symmetry. Each data point represents the percentage of asymmetric replication forks in a single

image with >20 replication structures from three independent experiments. The black line indicates the mean. (C) Representative DNA fiber images of fibroblasts from C1-C3, S5, S6, and S12-S14 with (+) and without (-) HU treatment. Scale bar, 5 µm. (D) Scheme illustrates CldU and IdU labelling of cells for 30 minutes each, with a 2-hour incubation with HU in between to halt DNA replication (top). Effect of HU treatment on the length of IdU tracks in fibroblasts from S5, S6, S12-S14, and three controls (bottom). Individual data points obtained from three independent experiments are shown, with the mean indicated by the black line. (E) Effect of HU treatment on fork stalling in fibroblasts from S5, S6, S12-S14, and three controls after normalization to the percentage of stalled forks in untreated cells. Quantification of individual data sets, expressed as fold change relative to the respective untreated cell line. (F) Scheme illustrates CldU and IdU labelling of cells for 15 and 45 minutes, respectively, with a 2-hour incubation with (+) or without (-) HU in between to halt DNA replication (top). The proportions of stalled replication forks were quantified in fibroblasts from C1, S5, and S14 relative to actively replicating forks and are presented as percentages (bottom). A minimum of 50 structures per experiment was analyzed for each cell line in two independent experiments. Each data point represents the mean of one experiment. Statistical significance was determined by one-way ANOVA followed by Dunnett's multiple comparisons test (A-D) or student's t-test (F). C1-C3, control fibroblasts; ns, not significant; S5, S6, S12-S14, subject fibroblasts. *$P \leq 0.05$; **$P \leq 0.01$; ***$P \leq 0.001$; ****$P \leq 0.0001$.

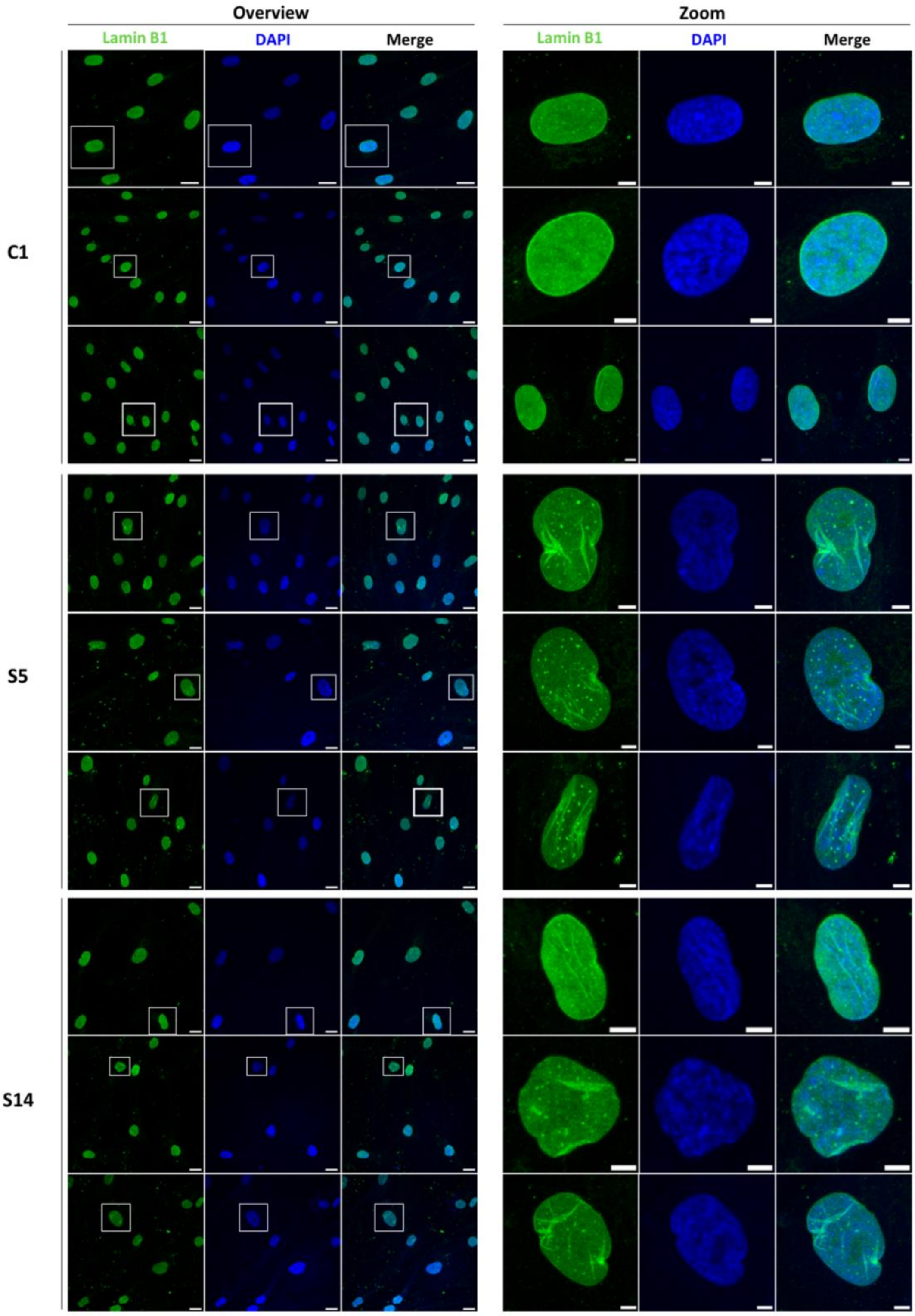


**Figure S11.** Altered lamina morphology in fibroblasts with biallelic *WDHD1* variants.

Immunofluorescence analysis of fibroblasts from control 1 (C1), S5, and S14. Cells were stained with an anti-Lamin B1 antibody, followed by an Alexa Fluor 488-conjugated secondary antibody (green) and DAPI (blue). Representative overview images are shown on the left. Scale bar, 20 µm. Exemplary zoom images on the right show aberrant lamina structures and distinct lamin B1-positive foci in cells from S5 and S14. Scale bar, 5 µm. S5, S14, subject fibroblasts.

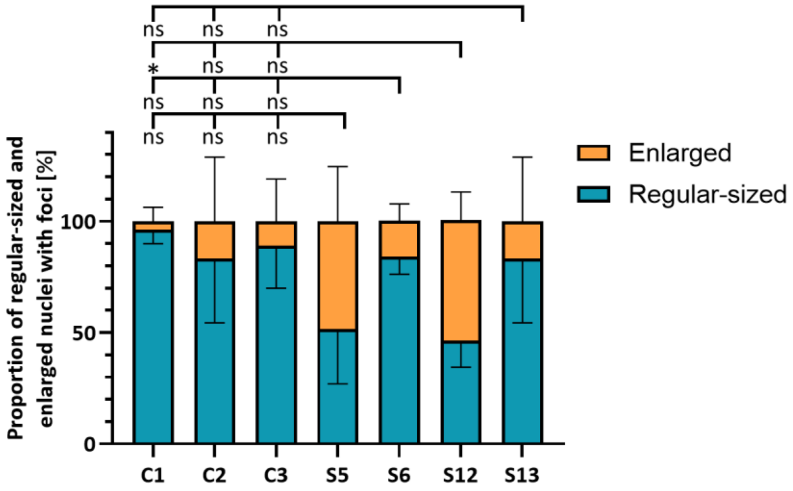


**Figure S12.** Proportion of regular-sized and enlarged nuclei containing ɣH2AX foci in control and subject cells.

Nuclei positive for γH2AX foci were categorized as regular-sized or enlarged, and the relative proportions of cells in each category were determined. The mean ± SD of three independent experiments in control and subject cells is shown. Statistical significance was determined by one-way ANOVA followed by Dunnett's multiple comparisons test. C1-C3, control fibroblasts; ns, not significant; S5, S6, S12, S13, subject fibroblasts. *$P \leq 0.05$.

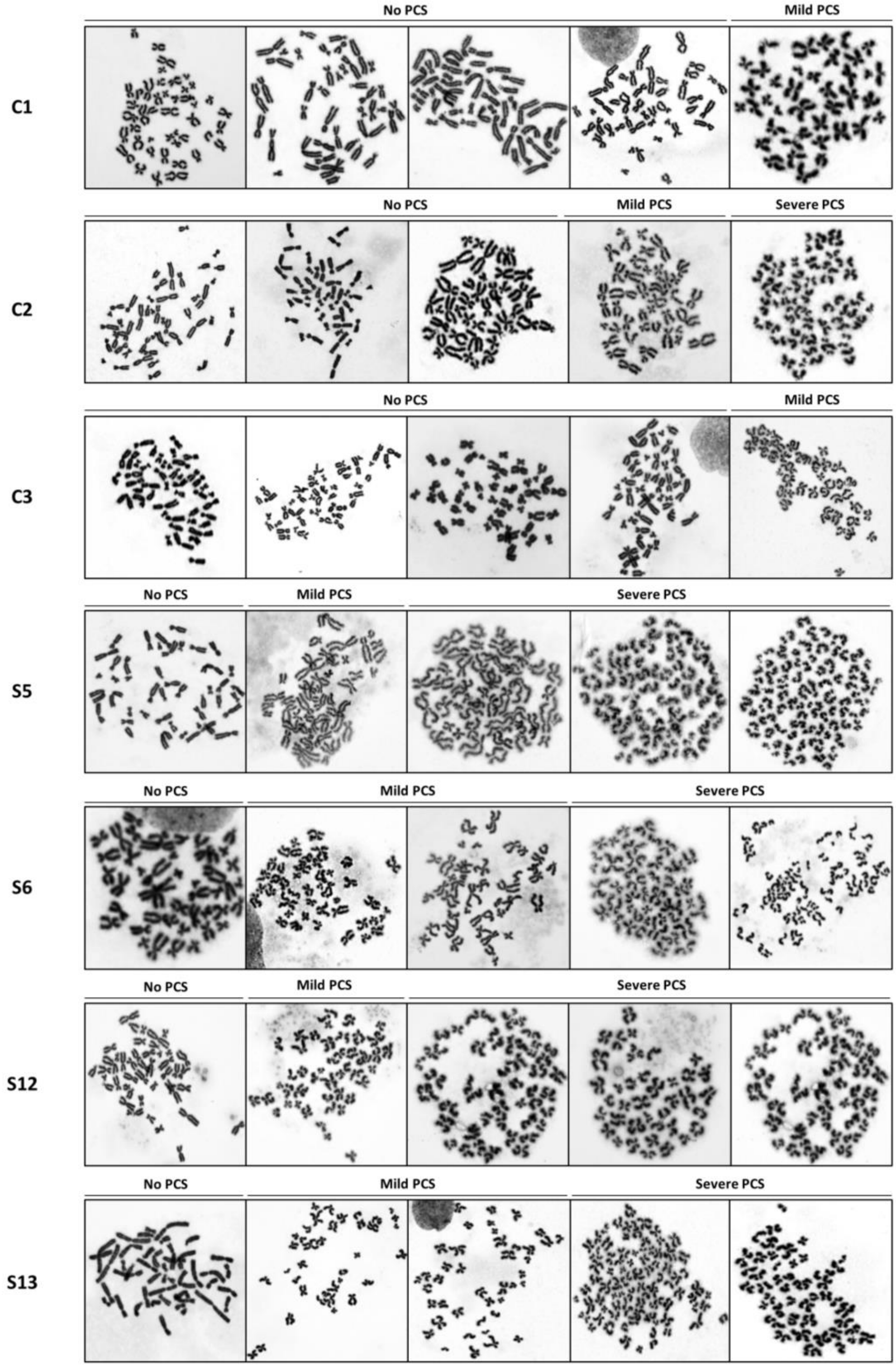


**Figure S13.** Analysis of premature chromatid separation (PCS) in fibroblasts with biallelic *WDHD1* variants.

Fibroblasts were arrested in mitosis, and metaphase spreads were prepared. Each metaphase (i.e. the complement of chromosomes from one cell) was classified as showing no PCS, mild PCS (>1 chromosome showing two separate chromatids with split centromere), or severe PCS (>50% of chromosomes showing two separate chromatids with split centromere). Representative images of each category are shown. C1-C3, control fibroblasts; S5, S6, S12, S13, subject fibroblasts.

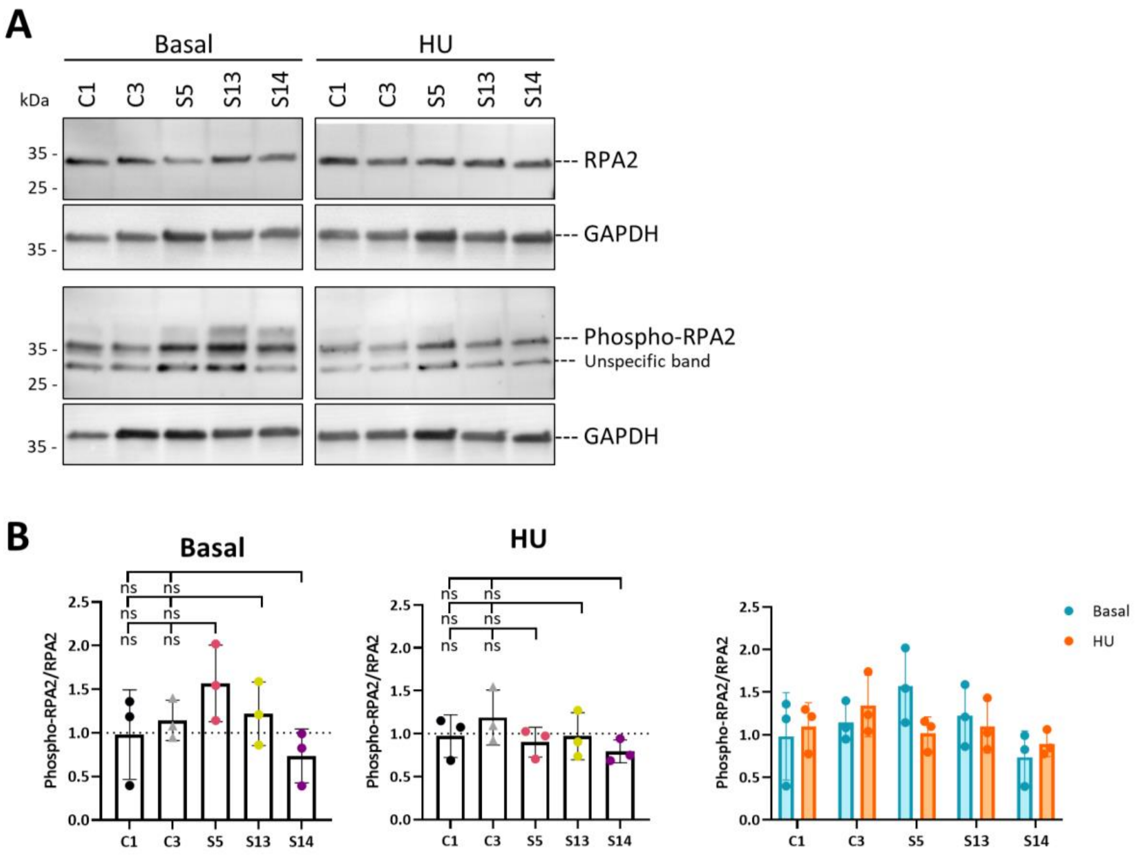


**Figure S14.** Determination of RPA2 and phospho-RPA2 levels in control and subject cells under basal and hydroxyurea-treated conditions.

(A) Representative immunoblots of fibroblast lysates from S5, S13, and S14 and controls under basal conditions (left) and following treatment with 2 mM hydroxyurea (HU) for 4 h (right). RPA2 and phospho-Ser4/8-RPA2 were detected using the indicated antibodies. An unspecific band is visible below the phospho-RPA2 band. GAPDH served as loading control for each blot. (B) Quantification of phospho-RPA2 relative to total RPA2. Band intensities of phospho-RPA2 and total RPA2 were first normalized to the corresponding GAPDH signal on each blot. The GAPDH-normalized phospho-RPA2 to RPA2 ratio is shown for subject and control cells cultivated under basal (left) and HU-treated (middle) conditions and is expressed relative to the mean of control cells. In the grouped analysis (right), values from HU-treated cells were normalized to the mean of control cells cultivated under basal conditions. The mean ± SD of three independent experiments is shown. Statistical significance was determined by one-way ANOVA followed by Dunnett's multiple comparisons test. C1, C3, control fibroblasts; ns, not significant; S5, S13, S14, subject fibroblasts.

## Supplemental tables:

## **Table S1.** Clinical characteristics of subjects with biallelic *WDHD1* variants

| | Family 1 | Family 2 | | Family 3 | | Family 4 | Family 5 | Family 6 | | Family 7 |
|---|---|---|---|---|---|---|---|---|---|---|
| | **Subject 1** | **Subject 2** | **Subject 3** | **Subject 4** | **Subject 5** | **Subject 6** | **Subject 7** | **Subject 8** | **Subject 9** | **Subject 10** |
| **Sex** | Male | Male | Female | Male | Female | Male | Female | Male | Female | Male |
| **Age at last examination** | 24 wk 5 d (gestation) | 22 wk 5 d (gestation) | 26 wk 3 d (gestation) | 27 wk 3 d (gestation) | 25 d | 9 d | 6 wk | 7 wk | 15 wk | 2 d |
| **Alive/Dead** | Termination of pregnancy | Prenatal death | Prenatal death | Prenatal death | Died at 35 d | Died at 9 d | Died at 6 wk | Died at 8 wk | Died at 15 wk | Died at 2 d |
| **Consanguinity** | Yes | + | + | Unknown | Unknown | + | + | + | + | + |
| **Genotype** | | | | | | | | | | |
| ***WDHD1* variant** (NM_007086.4/ NP_009017.1) | c.1117C>T; p.(Arg373*) homozygous | c.1769-1G>C homozygous | c.1769-1G>C homozygous | c.1769-1G>C homozygous | c.1769-1G>C homozygous | c.1769-1G>C homozygous | c.1769-1G>C homozygous | c.1769-1G>C homozygous | c.1769-1G>C homozygous | c.2971G>T; p.(Glu991*) homozygous |
| **Chromosome position hg38** | Chr14-54995639 | Chr14-54984861 | Chr14-54984861 | Chr14-54984861 | Chr14-54984861 | Chr14-54984861 | Chr14-54984861 | Chr14-54984861 | Chr14-54984861 | Chr14-54955640 |
| **Phenotype** | | | | | | | | | | |
| **Prenatal & neonatal features** | **Subject 1** | **Subject 2** | **Subject 3** | **Subject 4** | **Subject 5** | **Subject 6** | **Subject 7** | **Subject 8** | **Subject 9** | **Subject 10** |
| **Abnormalities in pregnancy** | IUGR, decreased fetal movements, borderline oligohydramnios | IUGR, oligohydramnios | IUGR | IUGR, oligohydramnios, abnormal umbilical artery doppler waveform during pregnancy | IUGR, oligohydramnios, abnormal umbilical artery doppler waveform during pregnancy | IUGR | IUGR | IUGR | IUGR | IUGR, oligohydramnios, decreased fetal movement |
| **Abnormal fetal morphology** | Microcephaly | Microcephaly | Microcephaly, cloverleaf skull | Microcephaly | – | Thickened nuchal skin fold | – | Microcephaly | Microcephaly | Retrognathia, bilateral dilatation of the renal pelvis, hyperechogenic kidneys |
| **Gestational age at birth** | – | – | – | – | 33 wk 5 d | 39 wk 3 d | 37 wk 4 d | 38 wk | 35 wk 1 d | 38 wk 2 d |
| **Length at birth** | Not reported | 33 cm (– 0.99 z) | Not reported | Not reported | 33 cm (– 4.00 z) | 42 cm (– 4.42 z) | 39 cm (– 4.71 z) | 46 cm (– 2.22 z) | 42 cm (– 1.94 z) | Not reported |
| **Weight at birth** | 510 g (– 3.0 z) | 530 g (– 0.78 z) | Not reported | 542 g (– 2.10 z) | 970 g (– 2.85 z) | 1475 g (– 4.74 z) | 1450 g (– 3.83 z) | 2235 g (– 2.47 z) | 1665 g (– 1.98 z) | 1100 g (– 5.19 z) |
| **Occipitofrontal circumference (OFC) at birth** | 18.5 cm (– 3.0 z) | Not reported | Not reported | 21 cm (– 4.30 z) | 24 cm (– 4.20 z) | 28.5 cm (– 5.33 z) | 28.5 cm (– 4.20 z) | 29.5 cm (– 3.86 z) | 28 cm (– 2.97 z) | Not reported |
| **Height at last examination** | Not applicable | Not applicable | Not applicable | Not applicable | 39.5 cm (– 5.10 z) | 43 cm (– 3.87 z) | 40 cm (– 6.17 z) | 48 cm (– 3.68 z) | 52 cm (– 3.19 z) | Not reported |
| **Weight at last examination** | Not applicable | Not applicable | Not applicable | Not applicable | 2.4 kg (– 1.90 z) | 2.2 kg (– 2.57 z) | 1.7 kg (– 4.41 z) | 2.8 kg (– 4.20 z) | 4.3 kg (– 1.90 z) | Not reported |
| **OFC at last examination** | Not applicable | Not applicable | Not applicable | Not applicable | 28.1 cm (– 7.80 z) | 29.5 cm (– 5.70 z) | 28 cm (– 12.12 z) | 30 cm (– 8.14 z) | 35 cm (– 5.41 z) | Not reported |

| Development | Subject 1 | Subject 2 | Subject 3 | Subject 4 | Subject 5 | Subject 6 | Subject 7 | Subject 8 | Subject 9 | Subject 10 |
|---|---|---|---|---|---|---|---|---|---|---|
| **Motor delay** | Not applicable | Not applicable | Not applicable | Not applicable | Not applicable | Not applicable | + | Not applicable | Not applicable | Not applicable |
| **Age at walking** | Not applicable | Not applicable | Not applicable | Not applicable | Not applicable | Not applicable | Not applicable | Not applicable | Not applicable | Not applicable |
| **Delayed speech and language development** | Not applicable | Not applicable | Not applicable | Not applicable | Not applicable | Not applicable | Not applicable | Not applicable | Not applicable | Not applicable |
| **Age at first words** | Not applicable | Not applicable | Not applicable | Not applicable | Not applicable | Not applicable | Not applicable | Not applicable | Not applicable | Not applicable |
| **Current number of words** | Not applicable | Not applicable | Not applicable | Not applicable | Not applicable | Not applicable | Not applicable | Not applicable | Not applicable | Not applicable |
| **Global developmental delay/intellectual disability** | Not applicable | Not applicable | Not applicable | Not applicable | Not applicable | Not applicable | Not applicable | Not applicable | Not applicable | Not applicable |
| **EEG abnormality** | Not applicable | Not applicable | Not applicable | Not applicable | Not done | Not done | Intermittent flattening of the brain wave pattern | Seizure | Seizure | Not done |
| **Neurological features** | **Subject 1** | **Subject 2** | **Subject 3** | **Subject 4** | **Subject 5** | **Subject 6** | **Subject 7** | **Subject 8** | **Subject 9** | **Subject 10** |
| **Abnormal muscle tone** | Not applicable | Not applicable | Not applicable | Not applicable | Generalized hypotonia | Neonatal hypotonia | Generalized hypotonia | Generalized hypotonia | Generalized hypotonia | Neonatal hypotonia |
| **Brain abnormalities** | Prenatal ultrasound: no brain abnormality found | Cavum septum pellucidum, cerebellar hypoplasia, dysplastic corpus callosum, enlarged fetal cisterna magna, small cerebral cortex | Abnormal lateral ventricle morphology, cerebral hypoplasia, lissencephaly | Prenatal MRI: Hypoplasia of the corpus callosum | Ultrasound: Hypoplasia of the corpus callosum, frontal pachygyria | Immature gyration, lateral ventricular asymmetry | Relatively immature gyration, narrow symmetrical ventricular system | Ultrasound normal | MRI: abnormal brain morphology, abnormal myelination, cerebral edema, abnormal cortical gyration (reduced) | Abnormal brain morphology |
| **Abnormality of the face** | **Subject 1** | **Subject 2** | **Subject 3** | **Subject 4** | **Subject 5** | **Subject 6** | **Subject 7** | **Subject 8** | **Subject 9** | **Subject 10** |
| **Face** | Not reported | Not reported | Not reported | Not reported | + | Not reported | + | Small face | Small face | Not reported |
| **Forehead** | Not reported | Not reported | Not reported | Not reported | Not reported | Not reported | Not reported | – | – | Not reported |
| **Ear** | Not reported | Not reported | Not reported | Not opened | Low-set ears, pointed ear | Low-set ears | – | – | – | Not reported |
| **Eye** | – | Microphthalmia | Not reported | Microphthalmia, downslanted palpebral fissures, short palpebral fissure, hypertelorism, epicanthus | Microphthalmia, down-slanted palpebral fissures, short palpebral fissure, hypertelorism, epicanthus | Deeply set eyes | Not reported | Microphthalmia | Microphthalmia | Not reported |
| **Nose** | Not reported | Not reported | Wide nose | Not reported | Bulbous nose, wide nose | Not reported | Not reported | – | – | Not reported |
| **Mouth** | – | Not reported | Not reported | Cleft palate, furrowed tongue | Narrow mouth | Not reported | Not reported | – | – | Cleft palate |
| **Chin** | Not reported | Not reported | Not reported | Mild retrognathia | Mild retrognathia | Short chin | Not reported | Mild retrognathia | – | Retrognathia |
| **Miscellaneous** | **Subject 1** | **Subject 2** | **Subject 3** | **Subject 4** | **Subject 5** | **Subject 6** | **Subject 7** | **Subject 8** | **Subject 9** | **Subject 10** |
| **Abnormal hair morphology** | Not applicable | Not applicable | Not applicable | Not applicable | – | Not reported | Not reported | Not reported | Not reported | – |
| **Hearing impairment** | Not applicable | Not applicable | Not applicable | Not applicable | Not reported | Not reported | Not reported | Not reported | Not reported | Not reported |
| **Visual impairment** | Not applicable | Not applicable | Not applicable | Not applicable | Cataract (bilateral), abnormal retinal vascular morphology, microcornea | Not reported | Optic atrophy | Cataract, retinal dystrophy, iris malposition | Retinal dystrophy | Not reported |

| | | | | | | | | | | |
|---|---|---|---|---|---|---|---|---|---|---|
| **Abnormal heart morphology** | Hypoplastic left heart, abnormal heart morphology | Ventricular septal defect | Small apical ventricular septal defect | Hypoplastic heart, abnormal anatomic location of the heart (left rotated), left ventricular dilatation | Patent foramen ovale | Patent ductus arteriosus | Minimal pericardial effusion | – | – | Abnormal heart morphology |
| **Abnormality of the respiratory system** | Not applicable | Not applicable | Not applicable | Not applicable | Respiratory insufficiency, highflow support | Respiratory insufficiency, highflow support | Neonatal respiratory distress,<br>respiratory distress | Respiratory insufficiency (mech. ventilated) | Respiratory insufficiency (mech. ventilated) | Not reported |
| **Abnormality of the endocrine system** | – | Not reported | Not reported | Hypoplasia of the thymus | Hyperinsulinemia, congenital hypothyroidism | – | Hyperinsulinemia | – | – | Not reported |
| **Abnormality of the immune system** | Not reported | Not reported | Not reported | Hypoplastic spleen | Recurrent infections | Not reported | Anti-herpes simplex antibody positivity, mild leukopenia | – | – | Not reported |
| **Abnormality of the digestive system** | Not reported | – | Abnormality of the gastrointestinal tract | Abnormal intestine morphology (malrotated) | Abnormal intestine morphology (pathological thickening of the mucosa),<br>mild ascites,<br>rectal prolapse | – | Ascites | Feeding difficulties, tube feeding | Feeding difficulties, tube feeding | Not reported |
| **Abnormality of the liver** | Not reported | Not reported | Not reported | Hepatic fibrosis, hepatic cysts | Decreased liver function, abnormal liver morphology (inhomogeneous), hepatic arterioportal fistulas, hypoplastic, intrahepatic gallbladder | Acute hepatic failure, cirrhosis, elevated hepatic iron concentration, giant cell hepatitis, cholestasis, suspected biliary atresia | Cirrhosis, acute hepatic failure, elevated hepatic iron concentration, giant cell hepatitis,<br>Hepatosplenomegaly | Cirrhosis (biopsy), acute hepatic failure, elevated hepatic iron concentration | Cirrhosis (biopsy at 6 wks), acute hepatic failure, elevated hepatic iron concentration | – |
| **Abnormality of metabolism/ homeostasis** | Not applicable | Not applicable | Not applicable | Not<br>applicable | Increased circulating lactate concentration, hypoglycemia | Not reported | Hypoglycemia, abnormality of amino acid metabolism, hyperammonemia | Hypoglycemia | Hypoglycemia | Not reported |
| **Abnormality of the genitourinary system** | Ambiguous genitalia, male | Not reported | Hyperechogenic right kidney | Enlarged kidneys | Vesicoureteral reflux (right), right-sided hydroureter, aplasia of the uterus | – | – | – | – | Ambiguous genitalia, male |
| **Abnormal skeletal morphology** | Not reported | Not reported | Dolichocephaly | Sandal gap | Hip dislocation (bilateral) | – | Skeletal dysplasia | – | – | Short long bones |
| **Abnormality of limbs** | Not reported | Not reported | Not reported | 2-3 toe syndactyly (right foot) | 2-4 toe syndactyly | Not reported | Abnormal foot morphology (hook feet) | 2-3 toe syndactyly (left foot) | – | Malposition of feet with external rotation |
| **Other abnormalities** | Not reported | Not reported | Not reported | – | Hypoesthesia | Not reported | Mild thrombocytopenia,<br>short stature | – | – | Not reported |

Table continues on next pages.

| | Family 8 | Family 9 | Family 10 | Family 11 | Family 12 | Family 13 | Family 14 |
|---|---|---|---|---|---|---|---|
| | **Subject 11** | **Subject 12** | **Subject 13** | **Subject 14** | **Subject 15** | **Subject 16** | **Subject 17** |
| **Sex** | Male | Female | Female | Male | Male | Female | Male |
| **Age at last examination** | 5 y 11 m | 8 y 10 m | 10 y 7 m | 3 y 1 m | 11 y 9 m | 16 y 9 m | 9 y |
| **Alive/Dead** | Alive | Alive | Alive | Alive | Alive | Alive | Alive |
| **Consanguinity** | – | – | – | – | Not reported | – | – |
| **Genotype** | | | | | | | |
| ***WDHD1* variant** (NM_007086.4/NP_009017.1) | c.1769-1G>C homozygous | c.1769-1G>C homozygous | c.1341+5_1341+6inv, c.3190-1G>A compound heterozygous | c.1341+5_1341+6inv, c.3157C>T; p.(Arg1053*) compound heterozygous | c.1287del; p.(Gln430Lysfs*37), c.2339G>A; p.(Arg780His) compound heterozygous | c.1341+5G>T, c.2531+1G>A compound heterozygous | c.505-1G>A, c.1342-6T>A compound heterozygous |
| **Chromosome position hg38** | Chr14-54984861 | Chr14-54984861 | Chr14-54991207-54991208, chr14-54941691 | Chr14-54991207-54991208, chr14-54944364 | Chr14-54991266, chr14-54963144 | Chr14-54962951 Chr14-54991208 | Chr14-55007376 Chr14-54989218 |
| **Phenotype** | | | | | | | |
| **Prenatal & neonatal features** | **Subject 11** | **Subject 12** | **Subject 13** | **Subject 14** | **Subject 15** | **Subject 16** | **Subject 17** |
| **Abnormalities in pregnancy** | Likely IUGR, oligohydramnios | IUGR, oligohydramnios | IUGR, oligohydramnios, small placenta | IUGR, oligohydramnios, small placenta (281 g), abnormal umbilical cord blood vessel morphology | IUGR | IUGR | IUGR |
| **Abnormal fetal morphology** | Limited antenatal care | Not reported | Microcephaly, webbed neck, wide intermamillary distance, aplasia/hypoplasia of the patella | Microcephaly | Microcephaly | Microcephaly | Small for gestational age |
| **Gestational age at birth** | 34 wk | 27 wk 5 d | 31 wk | 34 wk 1 d | 39 wk | 37 wk | 33 wk 6 d |
| **Length at birth** | Not reported | 35 cm (– 0.36 z) | 33 cm (– 2.30 z) | 43.5 cm (– 1.03 z) | 44 cm (– 3.20 z) | 40 cm (– 3.96 z) | 39 cm (– 2.20 z) |
| **Weight at birth** | 1015 g (– 3.12 z) | 1160 g (+ 0.59 z) | 935 g (– 1.75 z) | 1490 g (– 2.09 z) | 2810 g (– 1.40 z) | 1950 g (– 2.35 z) | 1380 g (– 2.03 z) |
| **Occipitofrontal circumference (OFC) at birth** | Not reported | 26 cm (+ 0.03 z) | 25 cm (– 1.95 z) | 28 cm (– 2.57 z) | Not reported | Not reported | 29 cm (– 1.34 z) |
| **Height at last examination** | 88.9 cm (– 5.71 z) | 93 cm (– 6.65 z) | 109 cm (– 4.10 z) | 73.5 cm (– 5.7 z) | 123.5 cm (– 3.30 z) | 129.8 cm (– 5.69 z) | 115.8 cm (– 3.11 z) |
| **Weight at last examination** | 8.9 kg (– 9.39 z) | 10.6 kg (– 8.82 z) | 15.9 kg (– 4.10 z) | 7.45 kg (– 5.34 z) | 23 kg (– 3.39 z) | 27.2 kg (– 6.82 z) | 20.5 kg (– 2.65 z) |
| **OFC at last examination** | 41.6 cm (– 8.13 z) | 42 cm (– 10.72 z) | 48.2 cm (– 3.58 z) | 45.6 cm (– 3.2 z) | 46.5 cm (– 4.60 z) | 50 cm (– 4.55 z) | 47 cm (– 2.9 z) |
| **Development** | **Subject 11** | **Subject 12** | **Subject 13** | **Subject 14** | **Subject 15** | **Subject 16** | **Subject 17** |
| **Motor delay** | + Mild gross motor delay, fine motor delay | + Mild gross motor delay | + | + | + | – | + Fine and gross motor delay |
| **Age at walking** | 20 m | 22 m | 3 y | 18 m | Not reported | 14 m | 22 m |

| | | | | | | | |
|---|---|---|---|---|---|---|---|
| **Delayed speech and language development** | +<br>Mild to moderate receptive and expressive speech delay, articulation difficulties | +<br>Mild to moderate receptive and expressive speech delay | +<br>Mild | +<br>Mild | + | – | Articulation difficulties |
| **Age at first words** | 3 y | 2 y 5 m<br>Single words | 2 y | 2 y | 3 y | 10 m | 15 m |
| **Current number of words** | Speaks in short sentences, both in Kurdish and German | 8 y<br>Speaks in short sentences, both in Kurdish and German | Normal number of words | Speaks words, no sentences | Speaks in short sentences | Completed basic education | Speaks in sentences, in mainstream education |
| **Global developmental delay/intellectual disability** | +<br>Mild to moderate | +<br>Degree not tested | +<br>Mild | +<br>Mild speech receptive, moderate expressive, short attention span | +<br>Mild | – | +<br>Developmental quotient 1st percentile |
| **EEG abnormality** | Not done<br>(no clinical concern for seizures) | – | Not done | – | Not done | Not done | Not done |
| **Neurological features** | **Subject 11** | **Subject 12** | **Subject 13** | **Subject 14** | **Subject 15** | **Subject 16** | **Subject 17** |
| **Abnormal muscle tone** | – | – | Generalized hypotonia | – | Neonatal hypotonia | Not reported | Generalized hypotonia |
| **Brain abnormalities** | Choroid fissure cysts | Lateral ventricular asymmetry, aqueductal stenosis | MRI normal (shortly after birth, not repeated) | Ultrasound normal (after birth) | MRI: left temporal lobe cyst | MRI: pituitary hypoplasia without other abnormalities | MRI: Supratentorial white matter signal abnormality most prominently involving the frontal lobes and temporal poles |
| **Abnormality of the face** | **Subject 11** | **Subject 12** | **Subject 13** | **Subject 14** | **Subject 15** | **Subject 16** | **Subject 17** |
| **Face** | Small face, low posterior hairline | Small face | Small face | Small face | Narrow face | Small face | – |
| **Forehead** | – | Small forehead | High forehead | High forehead | High forehead | High forehead | High Forehead |
| **Ear** | – | – | Low-set ears | – | Not reported | – | – |
| **Eye** | Short palpebral fissure, long eyelashes | Microphthalmia (left eye), small upslanted palpebral fissures, synophrys, hypertelorism | Hypertelorism, downslanted palpebral fissures | Short palpebral fissures | Microphthalmia, deeply set eyes, narrow palpebral fissure, microcornea,<br>strabismus | Puffy eyes, myopia | Hypertelorism, blepharophimosis, strabismus, epicanthus |
| **Nose** | Wide nasal bridge | Bulbous nose | Bulbous nose | Bulbous tip of the nose | – | Bulbous nose | Broad nose |
| **Mouth** | Thin vermilion border, carious teeth | Delayed eruption of primary teeth | High palate | Normal palate, thin upper lip, late dentition (first teeth 16 months) | – | Thin vermilion border,<br>high palate | Thin vermillion border, carious teeth |
| **Chin** | Pointed chin | Pointed chin | Mild retrognathia, pointed chin | Mild retroganthia | Pointed chin | Retrognathia | Pointed chin |
| **Miscellaneous** | **Subject 11** | **Subject 12** | **Subject 13** | **Subject 14** | **Subject 15** | **Subject 16** | **Subject 17** |
| **Abnormal hair morphology** | Hypertrichosis | – | – | – | – | – | Sparse hair |
| **Hearing impairment** | Not reported | – | – | – | Stenosis of the external auditory canal | – | – |
| **Visual impairment** | Myopic astigmatism, visual acuity appropriate for age, requires prescription glasses | Exotropia, microcornea, secondary cataract with synechiae, exudative retinal detachment (right) | Optic atrophy, glaucoma, abnormality of the fovea | Optic atrophy | Optic atrophy | – | Myopia |

| | | | | | | | |
|---|---|---|---|---|---|---|---|
| **Abnormal heart morphology** | Functionally bicuspid aortic valve, mildly dilated ascending aorta | – | Patent foramen ovale | – | Not reported | – | – |
| **Abnormality of the respiratory system** | Neonatal respiratory distress | – | Laryngotracheal stenosis | Neonatal respiratory distress (surfactant, extubation after 5h, CPAP 3 days) | Neonatal respiratory distress | – | Laryngotracheomalacia |
| **Abnormality of the endocrine system** | – | Congenital hypothyroidism | Anterior hypopituitarism, hypogonadotropic hypogonadism, growth hormone deficiency, treated with growth hormone since the age of 4 months | Low IGF1, treated with growth hormone from 21 month | Central hypothyroidism, central adrenal insufficiency, growth hormone deficiency, treated with growth hormone since the age of 3 y | Congenital hypothyroidism, treated with rhGH from 2 to 9 months without effective response (normal GH secretion) | – |
| **Abnormality of the immune system** | Recurrent infections | Recurrent infections of the gastrointestinal tract | Not reported | Not reported | – | – | – |
| **Abnormality of the digestive system** | Feeding difficulties, tube feeding, right inguinal hernia | Feeding difficulties, tube feeding, recurrent vomiting, percutaneous endoscopic jejunostomy (PEJ) | Feeding difficulties | Feeding difficulties | – | – | – |
| **Abnormality of the liver** | Abnormal liver morphology, suggested focal hepatic focal nodular hyperplasia, slightly raised liver enzymes, NB resolved on latest scan and blood aged 6 years | Intermittently elevated liver enzymes | Not reported | – | – | – | – |
| **Abnormality of metabolism/ homeostasis** | Very slightly raised ammonia | Not reported | Neonatal hypoglycemia | Neonatal hypoglycemia | Hypoglycemia | – | – |
| **Abnormality of the genitourinary system** | Cryptorchidism | – | – | Small genitalia | Bilateral cryptorchidism | – | Glandular hypospadias, bilateral cryptorchidism, small scrotum, chordee |
| **Abnormal skeletal morphology** | Slender long bones, moderate thoracic kyphosis with bullet shaped appearance to thoracic vertebrae but normal interpedicular distances | Hip dislocation (bilateral), marked delay in bone age, vertebral fusion (partial C5-C7) | Congenital hip dislocation, abnormality of skeletal maturation, epiphyseal dysplasia | Congenital dislocation of right hip, abnormality of skeletal maturation | Hip dislocation (bilateral) | Hip dislocation (bilateral) | Hip dislocation (bilateral), slender long bones, delayed bone age, possible epiphyseal dysplasia |
| **Abnormality of limbs** | Clinodactyly of the 5$^{th}$ finger | – | Aplasia/hypoplasia of the patella | Small patella | – | – | Lower limb asymmetry |
| **Other abnormalities** | High pitched voice, dental caries, demineralised teeth | High pitched voice | High pitched voice | High pitched voice | High pitched voice, microdontia | High pitched voice | High pitched voice |

+, present; –, absent; d, days; EEG, electroencephalography; IUGR, intrauterine growth retardation; m, months; MRI, magnetic resonance imaging; wk, weeks; y, year(s).

**Table S2.** Sequence of oligonucleotides used in this work.

| *WDHD1* primer sequences for variant validation and/or segregation analysis | | | |
|---|---|---|---|
| **Template** | **Exon/ Intron** | **Direction** | **Sequence (5´ → 3´)** |
| Genomic DNA (subject 5) | Intron 14 | Forward | CCTCAGTTGGATGGGTTATTCA |
| | Exon 15 | Reverse | CCTTGTAAGAGGAAGAGGGTCA |
| Genomic DNA | Exon 12 | Forward | GTCAAGAAGGCAGCATTCAC |
| Genomic DNA | Intron 12 (2) | Reverse | GCTCCAGGCTACTTAATCAAAG |
| Genomic DNA | Intron 25 | Forward | GTGTAAATCTTTAATGTGCTCTGC |
| Genomic DNA | Exon 26 | Reverse | CCTGCTTAAATGCAAAAGCTG |
| Genomic DNA | Intron 24 | Forward | GAATTAACAAGTGTAACTGTGTCTTCC |
| Genomic DNA | Intron 25 | Reverse | ACCATACCCAGCCAAAAGG |
| **Primer sequences for *WDHD1* transcript analysis** | | | |
| **Template** | **Exon/ Intron** | **Direction** | **Sequence (5´ → 3´)** |
| Complementary DNA (cDNA) | Exon 13 | Forward | TCCATGATACCTCCATACACCA |
| cDNA | Exon 15 | Reverse | CAGCTGAAAACCCAATCCAT |
| cDNA | Exon 16 | Reverse | ATGGATACCAACCACCCAGTAG |
| cDNA | Exon 25 | Forward | AAGACCGGGTTCCAGATGTG |
| cDNA | Exon 26 | Reverse | TCCTGCTTAAATGCAAAAGCTG |
| cDNA | Exon 11 | Forward | GTCACATCCTAGAAGATGATG |
| cDNA | Exon 13 | Reverse | GCAGCGAATAATTCCAATAGAGTTCC |
| cDNA | Intron 12 | Forward | GTAATCCTTGGATCTACACTTAAC |
| cDNA | 3´UTR | Reverse | TGCATTTGGAGGCAGAGTAATC |
| cDNA | Intron 12-Exon 13 | Reverse | CCACACTAAGAAGTTAAGTGTAGATCC |
| cDNA | Exon 24 | Forward | GCATCTGCAGCATCCTATTTCC |
| cDNA | Exon 6 | Forward | GATGGATCTGTCAGAGTGTGG |
| cDNA | Exon 9 | Reverse | ATATTGCCCACAGGGAGACC |
| cDNA | Exon 12 | Forward | CAAAAGCCATTTCAGTCAGG |
| cDNA | Exon 14 | Reverse | GTCCAGCAAGGCTGAATACC |
| **Primer sequences for colony PCR** | | | |
| **Template** | **Region** | **Direction** | **Sequence (5´ → 3´)** |
| pCR2.1 TOPO vector | T7 | Forward | TAATACGACTCACTATAGGG |
| pCR2.1 TOPO vector | M13 | Reverse | CAGGAAACAGCTATGAC |

Table continues on next page.

| **Primer sequences for quantitative *WDHD1* transcript analysis** | | | |
|---|---|---|---|
| **Template** | **Exon** | **Direction** | **Sequence (5´ → 3´)** |
| cDNA | 13 | Forward | TGATACCTCCATACACCATGCAAC |
| cDNA | 14 | Reverse | CACTCTTTGCTTGAATCCCAAGAAC |
| cDNA | 15 | Forward | GGTGACCCTCTTCCTCTTACAAG |
| cDNA | 16 | Reverse | CCAACCACCCAGTAGTGATCAG |

**Table S3:** Number of cells analyzed for quantification of γH2AX foci and nuclear morphology in three independent experiments.

| | **C1** | **C2** | **C3** | **S5** | **S6** | **S12** | **S13** |
|---|---|---|---|---|---|---|---|
| **n = 1** | 63 | 52 | 45 | 47 | 37 | 73 | 55 |
| **n = 2** | 78 | 41 | 44 | 56 | 73 | 43 | 51 |
| **n = 3** | 61 | 71 | 103 | 23 | 90 | 33 | 47 |
| **Total** | 202 | 164 | 192 | 126[a] | 200 | 149 | 153 |

[a]As S5 fibroblasts show a severe proliferation defect, the target number of at least 150 nuclei could not be reached for the analysis.

**Supplemental acknowledgements**

Regarding subject 10: In some cases, clinical genomic sequencing data was generated by a NATA-accredited laboratory and transferred to the UDN-Aus study for research analysis. This was made possible by NSW Health Pathology, Genomics Laboratory, Prince of Wales Hospital, Randwick, NSW, Australia and the Sydney Children's Hospitals Network and Rare Diseases NSW undiagnosed disease program GeneAdd.

**Supplemental material and methods**

*Genetic analyses*

Subject 1

Trio whole-exome sequencing (WES) was performed on DNA extracted from amniotic fluid of subject 1 and parents' peripheral blood using standard protocols. Libraries preparation was performed using a TWIST BioScience Library EF KIT (TWIST- Alliance) kit, with libraries analyzed on an Illumina NovaSeq 6000 Sequencing System (Illumina San Diego, CA) in paired-end mode. Reads were aligned to Human Genome Reference Sequence GRCh38, and single nucleotide and short insertion/deletion variants were identified using the DRAGEN Enrichment pipeline (v4.4.6) on the Illumina BaseSpace DRAGEN Server. Variant filtering, prioritisation, and reporting were performed using the Genomics Annotation and Interpretation Application (GAIA) in-house pipeline. Variants were filtered based on inheritance pattern, impact, frequency, zygosity and in silico pathogenicity scores. The data analysis pipeline is based on Gemini (v18) with annotation from VEP and dbNSFP. Copy number variants (CNV) were identified by three CNV callers: CoNIFER, DECoN, and XHMM.[1-3] CNVs were then filtered according to morbid gene content relevant to the referral phenotype and the number of callers identifying a potential CNV. Each variant was evaluated based on the available information from the following: databases (typically including, but not limited to, the latest public version of ClinVar, LSDBs and gnomAD), published literature, clinical correlation, segregation analysis, functional studies, and predicted functional or splicing impact using evolutionary conservation analysis and computational

tools. Variants were classified following the joint consensus recommendations of the American College of Medical Genetics and Genomics and the Association for Molecular Pathology for the interpretation of sequence variants[4] as well as integrating guidance from the best practice guidelines for variant interpretation published by the Association for Clinical Genomic Science (https://www.acgs.uk.com/quality/best-practice-guidelines/). Variants were reported according to HGVS nomenclature. The possibility of significant maternal cell contamination was excluded by comparative genotyping at seven informative, unlinked STR loci using the Promega PowerPlex 16HS Kit.

Subjects 2 and 3

Genomic DNA extraction from EDTA blood and amniocytes was carried out by using the QIAamp DNA Blood Mini Kit (Qiagen, Hilden, Germany) according to the manufacturer's protocol. DNA concentration was measured with the Qubit system (dsDNA Assay Kit; Thermo Fisher Scientific, Waltham, MA, USA). WES on DNA of subjects 2 and 3 and parents was performed by using an xGen Exome Research Panel v2, targeting 19,433 genes (Integrated DNA Technology, Coralville, IA, USA) according to the manufacturer's protocol. Genomic DNA was processed via hybrid capture, with target regions enriched and amplified by PCR using the Lotus DNA Library Prep Kit (Integrated DNA Technology). Massively parallel sequencing was performed on the NextSeq 2000 Sequencing System (Illumina, San Diego, CA, USA). WES quality control criteria required a minimum coverage of 25× across 100% of the targeted regions. Further details on the protocols used are available upon request.

Subjects 4 and 5

DNA was isolated from EDTA blood samples of subjects 4 and 5 and parents, fragmented to 350-400 bp, and quality-checked. Libraries were prepared with the Human Core Exome Kit (Twist Bioscience) and sequenced on an Illumina NovaSeq 6000 (2 × 100 bp paired-end), achieving >20× coverage for >95% of the RefSeq target region. Raw reads were demultiplexed and aligned to GRCh37 using BWA-Mem v0.7.11 (https://ieeexplore.ieee.org/document/8820962),[5] ensuring >95% of targets reached

≥20× coverage. Variants (SNVs and indels) were called with HaplotypeCaller v3.8, and joint genotyping applied via GLnexus (preset GATK).[6] Quad variant analysis was conducted in VarFish.[7]

Subject 6

DNA was extracted from whole blood samples of subject 6 and parents. For all samples WES was performed. DNA enrichment and library preparation were carried out using xGen DNA Lib Prep EZ UNI (Integrated DNA Technologies, Inc., Coralville). Sequencing was conducted on an Illumina NovaSeq X Plus sequencer (Illumina San Diego, CA). Reads were aligned to the human reference genome GRCh38 using megSAP (version megSAP-2022_08-152-gdd675f86; https://github.com/imgag/megSAP). Variant prioritization and visualization were performed with GSvar (version ngs-bits-2024_08-35-g5b45dc95; https://github.com/imgag/ngs-bits), Integrative Genomics Viewer (IGV; version 2.16.0)[8] and Alamut® visual Plus (version 1.8.1; Interactive Biosoftware, Rouen, France).

Subjects 7 and 12

DNA was isolated from EDTA blood samples of subjects 7, 12, and their parents. WES was performed on genomic DNA from subjects 6 and 11 and parents, as previously described.[9] Analysis of the WES data was performed using an in-house pipeline (Exome Variant Annotation Database; https://github.com/mri-ihg/EVAdb). The Burrows-Wheeler Aligner (v.0.7.5a) was used to align sequencing reads to the human genome assembly GRCh37/hg19 (UCSC Genome Browser).[5] Single nucleotide variants, as well as small insertions and deletions, were detected using the Genome Analysis Toolkit.[10] ExomeDepth was used to detect copy number variants.[11] Mitochondrial DNA variants were detected from exonic data as previously reported outlined.[12]

Subjects 8 and 9

DNA was extracted from whole blood samples of subjects 8 and 9, their parents, and two siblings. For all samples WES was performed. DNA enrichment and library preparation were carried out using the

xGen Exome Research Panel (v1 or v2; Integrated DNA Technologies, Inc., Coralville) or TruSeq Exome (Illumina, San Diego, CA). Sequencing was conducted on a NextSeq 500 or on a NovaSeq6000 sequencer. Reads were aligned to the human reference genome (GRCh38) using megSAP (version megSAP-2022_08-152-gdd675f86; https://github.com/imgag/megSAP).

Variant prioritization and visualization were performed with GSvar (version ngs-bits-2024_08-35-g5b45dc95; https://github.com/imgag/ngs-bits), Integrative Genomics Viewer (IGV; v2.16.0)[8] and Alamut® visual Plus (v1.8.1; Interactive Biosoftware, Rouen, France).

Subject 10

Genomic DNA was isolated from amniotic fluid obtained from the mother of subject 10. Double stranded DNA capture baits against approximately 36.5 Mb of the human coding exome (targeting >98% of the coding RefSeq from the human genome build GRCh37/hg19) were used to enrich target regions from fragmented genomic DNA with the Twist Human Core Exome Plus kit. The generated library was sequenced on an Illumina platform to obtain at least 20x coverage depth for >98% of the targeted bases. An in-house bioinformatics pipeline, including read alignment to GRCh37/hg19 genome assembly, variant calling (single nucleotide and small deletion/insertion variants), annotation and comprehensive variant filtering was applied. All variants with minor allele frequency (MAF) of less than 1% in the gnomAD database, and disease-causing variants reported in HGMD®, in ClinVar or in CentoMD® were considered. The investigation for relevant variants was focused on coding exons and flanking +/-20 intronic nucleotides of genes. Variants with low quality and/or unclear zygosity were confirmed by orthogonal methods. Consequently, a specificity of >99.9% for all reported variants was warranted.

To exclude maternal cell contamination, 15 STR autosomal markers plus amelogenin were analyzed using the PowerPlex 16HS multiplex kit (Promega Corporation, Madison, USA).[13]

Subject 11

DNA was isolated from EDTA blood samples of subject 11 and parents and analyzed using WES at the Victorian Clinical Genetics Service (VCGS). As no diagnosis was reached the patient and their parents were recruited into the Undiagnosed Disease Network Australia Research Program, and the Sydney Children's Hospitals Network Undiagnosed Disease Program GeneAdd. Reanalysis of the existing trio whole-genome sequencing (WGS) was performed in a research setting at the Centre for Population Genomics following the DRAGEN GATK best practices pipeline. Reads were aligned to the hg38 reference genome using Dragmap (v1.3.0). Cohort-wide joint calling of single nucleotide variants and small insertion/deletion variants was performed using GATK HaplotypeCaller (v4.1.4.1) with "dragen-mode" enabled. Variants were annotated using VEP v105 and loaded into the web-based variant filtration platform, seqr.[14] Sample sex and relatedness quality checks were performed using Somalier (v0.2.15).[15] Variants were initially curated utilising the transcript predicted to be the most deleterious to the protein and were reported in accordance with HGVS nomenclature. Where no causative variants were identified within the prioritised gene lists, curation was expanded to "off-Mendeliome" genes covered by the original clinical test. Analysis of Short Tandem Repeats was performed using STRipy v2.2 pipeline.[16] Structural variant (SV) calling from short-read WGS data was performed using GATK-SV.[17] The resulting callset was then loaded into CPG instance of seqr for analysis. The GATK mitochondrial variant calling pipeline was used to call homoplasmic and heteroplasmic variants in mtDNA from WGS data using mitochondria mode of GATK MuTect2[18] and a MitoReport (https://github.com/bioinfomethods/mitoreport) was generated for analysis.

Subject 13

WES of isolated DNA from subject 13 and parents was performed at the CeGaT GmbH laboratory (Tübingen, Germany). The coding and flanking intronic regions were enriched using the SureSelect™ target enrichment system (Agilent Technologies, Santa Clara, CA) and were sequenced using the Illumina HiSeq/NovaSeq system. Illumina bcl2fastq2 was used for base calling and demultiplexing. High quality reads were aligned to the human reference genome (hg19) using the Burrows-Wheeler

Aligner (https://bio-bwa.sourceforge.net/). Variants were annotated based on several internal and external databases. Variant classification was performed according to ACMG guidelines for pathogenicity assessment.

Subject 14

Genomic DNA was extracted from peripheral blood samples of subject 14 and parents using standard procedures. Structural genomic variants in the proband were analyzed using a high-resolution single nucleotide polymorphism (SNP) array (Infinium Global Screening Array-24+ v3.0 Kit, GSA-Cyto, Illumina), which includes approximately 700,000 markers with an average inter-marker distance of 4 kb according to the manufacturer's protocol. The resulting karyotype was arr(X,Y)x1,(1-22)x2.
WES was performed using genomic DNA from subject 14, as previously described.[19] Briefly, human protein-coding genes (36.8 Mb in total) were captured from genomic DNA with the Twist Comprehensive Exome Panel and with the Mitochondrial Panel (Twist Bioscience, San Francisco, CA); reagents from the same kits were used to prepare DNA libraries, which were sequenced on a HiSeq platform (Illumina, San Diego, CA) with 150 bp read length in paired-end sequencing mode. The obtained sequencing reads were aligned to the human reference genome (GRCh38) (University of California Santa Clara, Santa Clara, CA) and single nucleotide variants and small indels were called with the Genome Analysis Toolkit (GATK) version 4.0 (https://github.com/broadinstitute/gatk). Sequencing reads were also aligned to the human reference genome (GRCh37) with SeqNext v5.0 (JSI, Kippenheim, Germany). The GRCh37 pipeline includes single nucleotide variants and small indels calling and the detection of single and multiple exon deletions and duplications.

Called variants were filtered for autosomal recessive mode of inheritance (including homozygous variants and two heterozygous variants in the same gene), for X-linked and autosomal dominant inheritance in the proband. Variants were filtered based on their predicted impact on splicing and protein function (missense, nonsense, intronic variants at exon-intron boundaries ranging from -15

to +15, in-frame indels, and frameshift). Only variants with an allele frequency of <0.01 in the gnomAD database (https://gnomad.broadinstitute.org/) were retained. Variants were evaluated *in silico* for pathogenicity by CADD (http://cadd.gs.washington.edu/score); missense variants were evaluated by PolyPhen-2 (http://genetics.bwh.harvard.edu/pph2) and SIFT; and splice site variants were evaluated using SpliceAI lookup (https://spliceailookup.broadinstitute.org/). *WDHD1* variants were validated in subject 13 and segregated in parents by Sanger sequencing using the BigDye terminator v3.1 chemistry on an ABI PRISM 3100 Genetic Analyzer (Applied Biosystems, Fisher Scientific). Sanger traces were evaluated with the Sequencher software.

<u>Subject 15</u>

WGS was performed within the framework of the National Genetic Initiative "100 000+Me". Genomic DNA from subject 15 and parents was sequenced on the DNBSEQ-T7 platform using paired-end sequencing (PE150). Library preparation was carried out with a PCR-free protocol involving enzymatic fragmentation (MGI). Variant nomenclature followed the guidelines of the Human Genome Variation Society (HGVS; http://varnomen.hgvs.org/recommendations/DNA (v20.05). The search for the genetic cause was conducted using the NGS-DATA software (https://ngs-data-ccu.epigenetic.ru/main/; accessed on May 24, 2025). Filtered variants were annotated according to HGVS nomenclature and referenced against the MANE Select transcript for the *WDHD1* gene. Variant classification was performed according to ACMG guidelines for pathogenicity assessment.

<u>Subject 16</u>

DNA was extracted from peripheral blood leukocytes of subject 16 and parents using standard protocols. WES on subject 16's DNA was performed as previously described.[20,21] Libraries were prepared with the Twist Exome 2.0 kit (Twist Bioscience, South San Francisco, CA, USA) following the manufacturer's instructions. Sequencing was performed on an Illumina NovaSeq 6000 (Illumina San Diego, CA) platform in paired-end mode. Reads were aligned to the GRCh37/hg19 human genome assembly

using the Burrows-Wheeler Aligner, and variants were called with Freebayes and GATK. CNVs were analyzed from WES data using clinCNV. Variants were analyzed using the Franklin by Genoox platform (franklin.genoox.com). Variants with a minor allele frequency <0.1% in population databases (gnomAD and ABraOM)[22,23] and located in coding regions or canonical splice sites were retained. Variants were categorized as high-impact (rare, protein-truncating variants: nonsense, canonical splice-site, frameshift) or moderate-impact (rare missense variants predicted deleterious [REVEL ≥0.64] or splice-altering [dbscSNV ≥0.70, SpliceAI ≥0.22]). Final candidate variants were prioritized based on zygosity, inheritance, gene function, protein expression, relevant phenotypes in humans or animal models, and literature or database evidence. *WDHD1* variants were validated and segregated in parents by Sanger sequencing using the BigDye terminator on an ABI PRISM 3100 Genetic Analyzer (Applied Biosystems, Fisher Scientific).

<u>Subject 17</u>

Reanalysis of the existing trio WGS was performed in a research setting at the Centre for Population Genomics following the DRAGEN GATK best practices pipeline. Reads were aligned to the hg38 reference genome using Dragmap (v1.3.0). Cohort-wide joint calling of single nucleotide variants and small insertion/deletion variants was performed using GATK HaplotypeCaller (v4.1.4.1) with "dragen-mode" enabled. Variants were annotated using VEP v105 and loaded into the web-based variant filtration platform, seqr.[14] Sample sex and relatedness quality checks were performed using Somalier (v0.2.15).[15] Variants were initially curated utilising the transcript predicted to be the most deleterious to the protein and were reported in accordance with HGVS nomenclature. Where no causative variants were identified within the prioritised gene lists, curation was expanded to "off-Mendeliome" genes covered by the original clinical test. Analysis of Short Tandem Repeats was performed using STRipy v2.2 pipeline.[16] Structural variant (SV) calling from short-read WGS data was performed using GATK-SV.[17] The resulting callset was then loaded into CPG instance of seqr for analysis. The GATK mitochondrial variant calling pipeline was used to call homoplasmic and heteroplasmic variants in

mtDNA from WGS data using mitochondria mode of GATK MuTect2[18] and a MitoReport (https://github.com/bioinfomethods/mitoreport) was generated for analysis. Variant(s) have been classified according to the American College of Medical Genetics and Genomics and the Association for Molecular Pathology joint consensus recommendations for the interpretation of sequence variants[4] with ClinGen recommendations.

*Variant validation by Sanger sequencing*

The biallelic *WDHD1* variants (NM_007086.4) or the reference sequence were validated and/or segregated in fibroblast-derived DNA from the subjects and in fibroblasts from healthy donors using Sanger sequencing. Primer sequences are described in **Supplementary Table 2**. PCR amplicons were sequenced using the ABI BigDye Terminator Sequencing kit (Applied Biosystems) and an automated capillary sequencer (ABI 3500, Applied Biosystems). Sequence electropherograms were analyzed using Chromas v2.6.6 (Technelysium Pty Ltd).

**Supplemental references**


1. Fowler, A., Mahamdallie, S., Ruark, E., Seal, S., Ramsay, E., Clarke, M., Uddin, I., Wylie, H., Strydom, A., Lunter, G., and Rahman, N. (2016). Accurate clinical detection of exon copy number variants in a targeted NGS panel using DECoN. Wellcome Open Res *1*, 20. 10.12688/wellcomeopenres.10069.1.
2. Fromer, M., Moran, J.L., Chambert, K., Banks, E., Bergen, S.E., Ruderfer, D.M., Handsaker, R.E., McCarroll, S.A., O'Donovan, M.C., Owen, M.J., et al. (2012). Discovery and statistical genotyping of copy-number variation from whole-exome sequencing depth. Am J Hum Genet *91*, 597-607. 10.1016/j.ajhg.2012.08.005.
3. Krumm, N., Sudmant, P.H., Ko, A., O'Roak, B.J., Malig, M., Coe, B.P., Project, N.E.S., Quinlan, A.R., Nickerson, D.A., and Eichler, E.E. (2012). Copy number variation detection and genotyping from exome sequence data. Genome Res *22*, 1525-1532. 10.1101/gr.138115.112.
4. Richards, S., Aziz, N., Bale, S., Bick, D., Das, S., Gastier-Foster, J., Grody, W.W., Hegde, M., Lyon, E., Spector, E., et al. (2015). Standards and guidelines for the interpretation of sequence variants: a joint consensus recommendation of the American College of Medical Genetics and Genomics and the Association for Molecular Pathology. Genet Med *17*, 405-424. 10.1038/gim.2015.30.
5. Li, H., and Durbin, R. (2009). Fast and accurate short read alignment with Burrows-Wheeler transform. Bioinformatics *25*, 1754-1760. 10.1093/bioinformatics/btp324.
6. Yun, T., Li, H., Chang, P.C., Lin, M.F., Carroll, A., and McLean, C.Y. (2021). Accurate, scalable cohort variant calls using DeepVariant and GLnexus. Bioinformatics *36*, 5582-5589. 10.1093/bioinformatics/btaa1081.
7. Holtgrewe, M., Stolpe, O., Nieminen, M., Mundlos, S., Knaus, A., Kornak, U., Seelow, D., Segebrecht, L., Spielmann, M., Fischer-Zirnsak, B., et al. (2020). VarFish: comprehensive DNA variant analysis for diagnostics and research. Nucleic Acids Res *48*, W162-W169. 10.1093/nar/gkaa241.
8. Robinson, J.T., Thorvaldsdottir, H., Winckler, W., Guttman, M., Lander, E.S., Getz, G., and Mesirov, J.P. (2011). Integrative genomics viewer. Nat Biotechnol *29*, 24-26. 10.1038/nbt.1754.
9. Zech, M., Jech, R., Boesch, S., Skorvanek, M., Weber, S., Wagner, M., Zhao, C., Jochim, A., Necpal, J., Dincer, Y., et al. (2020). Monogenic variants in dystonia: an exome-wide sequencing study. Lancet Neurol *19*, 908-918. 10.1016/S1474-4422(20)30312-4.
10. Van der Auwera, G.A., Carneiro, M.O., Hartl, C., Poplin, R., Del Angel, G., Levy-Moonshine, A., Jordan, T., Shakir, K., Roazen, D., Thibault, J., et al. (2013). From FastQ data to high confidence variant calls: the Genome Analysis Toolkit best practices pipeline. Curr Protoc Bioinformatics *43*, 11 10 11-11 10 33. 10.1002/0471250953.bi1110s43.
11. Plagnol, V., Curtis, J., Epstein, M., Mok, K.Y., Stebbings, E., Grigoriadou, S., Wood, N.W., Hambleton, S., Burns, S.O., Thrasher, A.J., et al. (2012). A robust model for read count data in exome sequencing experiments and implications for copy number variant calling. Bioinformatics *28*, 2747-2754. 10.1093/bioinformatics/bts526.
12. Wagner, M., Berutti, R., Lorenz-Depiereux, B., Graf, E., Eckstein, G., Mayr, J.A., Meitinger, T., Ahting, U., Prokisch, H., Strom, T.M., and Wortmann, S.B. (2019). Mitochondrial DNA mutation analysis from exome sequencing-A more holistic approach in diagnostics of suspected mitochondrial disease. J Inherit Metab Dis *42*, 909-917. 10.1002/jimd.12109.
13. Trujillano, D., Bertoli-Avella, A.M., Kumar Kandaswamy, K., Weiss, M.E., Koster, J., Marais, A., Paknia, O., Schroder, R., Garcia-Aznar, J.M., Werber, M., et al. (2017). Clinical exome sequencing: results from 2819 samples reflecting 1000 families. Eur J Hum Genet *25*, 176-182. 10.1038/ejhg.2016.146.
14. Pais, L.S., Snow, H., Weisburd, B., Zhang, S., Baxter, S.M., DiTroia, S., O'Heir, E., England, E., Chao, K.R., Lemire, G., et al. (2022). seqr: A web-based analysis and collaboration tool for rare disease genomics. Hum Mutat *43*, 698-707. 10.1002/humu.24366.

15. Pedersen, B.S., Bhetariya, P.J., Brown, J., Kravitz, S.N., Marth, G., Jensen, R.L., Bronner, M.P., Underhill, H.R., and Quinlan, A.R. (2020). Somalier: rapid relatedness estimation for cancer and germline studies using efficient genome sketches. Genome Med *12*, 62. 10.1186/s13073-020-00761-2.
16. Halman, A., Dolzhenko, E., and Oshlack, A. (2022). STRipy: A graphical application for enhanced genotyping of pathogenic short tandem repeats in sequencing data. Hum Mutat *43*, 859-868. 10.1002/humu.24382.
17. Collins, R.L., Brand, H., Karczewski, K.J., Zhao, X., Alfoldi, J., Francioli, L.C., Khera, A.V., Lowther, C., Gauthier, L.D., Wang, H., et al. (2020). A structural variation reference for medical and population genetics. Nature *581*, 444-451. 10.1038/s41586-020-2287-8.
18. Laricchia, K.M., Lake, N.J., Watts, N.A., Shand, M., Haessly, A., Gauthier, L., Benjamin, D., Banks, E., Soto, J., Garimella, K., et al. (2022). Mitochondrial DNA variation across 56,434 individuals in gnomAD. Genome Res *32*, 569-582. 10.1101/gr.276013.121.
19. Hackl, L., Haberlandt, E., Muller, T., Piribauer, S., Garczarczyk-Asim, D., Zoggeler, T., Karall, D., Zschocke, J., and Janecke, A.R. (2025). Homozygous DHCR7 p.Val330Met Variant Associated with Mild Non-Syndromic Intellectual Disability and Elevated Serum 7-Dehydrocholesterol Levels in Two Siblings. Genes (Basel) *16*. 10.3390/genes16070838.
20. Kim, G.J., Vasco de Albuquerque Albuquerque, E., Rezende, R.C., De Polli Cellin, L., Santillan Vasconez, A.M., Krepischi, A.C.V., Santana, L., Lerario, A.M., de Souza, V., Scalco, R., and Jorge, A.A.L. (2025). Exome sequencing of patients with syndromic tall stature reveals four novel candidate genes. Endocr Connect *14*. 10.1530/EC-25-0137.
21. Rezende, R.C., Menezes de Andrade, N.L., Branco Dantas, N.C., de Polli Cellin, L., Victorino Krepischi, A.C., Lerario, A.M., and de Lima Jorge, A.A. (2024). Exome Sequencing Identifies Multiple Genetic Diagnoses in Children with Syndromic Growth Disorders. J Pediatr *265*, 113841. 10.1016/j.jpeds.2023.113841.
22. Chen, S., Francioli, L.C., Goodrich, J.K., Collins, R.L., Kanai, M., Wang, Q., Alfoldi, J., Watts, N.A., Vittal, C., Gauthier, L.D., et al. (2024). A genomic mutational constraint map using variation in 76,156 human genomes. Nature *625*, 92-100. 10.1038/s41586-023-06045-0.
23. Naslavsky, M.S., Scliar, M.O., Yamamoto, G.L., Wang, J.Y.T., Zverinova, S., Karp, T., Nunes, K., Ceroni, J.R.M., de Carvalho, D.L., da Silva Simoes, C.E., et al. (2022). Whole-genome sequencing of 1,171 elderly admixed individuals from Sao Paulo, Brazil. Nat Commun *13*, 1004. 10.1038/s41467-022-28648-3.
